# Single-step genome-wide association study of milk somatic cell scores across multi-cattle breeds in Ethiopia

**DOI:** 10.1080/10495398.2025.2586262

**Published:** 2025-11-18

**Authors:** Tegegn Fantahun Chernet, Getinet Mekuriaw Tarekegn, Okeyo Mwai Ally, Selam Meseret, Raphael Mrode, Enyew Negussie, Zewdu Edea Bedada, Gebregziabher Gebreyohanes, Chinyere Ekine-Dzivenu, Asrat Tera, Tesfaye Sisay Tessema

**Affiliations:** a Biotechnology Department, Institute of Biotechnology, Addis Ababa University, Addis Ababa, Ethiopia; b Animal Biotechnology Directorate, Bio and Emerging Technology Institute, Addis Ababa, Ethiopia; c School of Veterinary Medicine and Biosciences (SVMB), Scotland’s Rural College (SRUC), Edinburgh, UK; d Livestock Genetics Department, ILRI, Nairobi, Kenya; e Livestock Genetics Department, International Livestock Research Institute (ILRI), Addis Ababa, Ethiopia; f Animal Genetics Department, Natural Resources Institute (Luke), Helsinki, Finland; g Bioinformatics and Genomics Directorate, Bio and Emerging Technology Institute, Addis Ababa, Ethiopia; h Animal Genetic Improvement and Research Department, Livestock Development Institute, Addis Ababa, Ethiopia; i Health Biotechnology Directorate, Institute of Biotechnology, Addis Ababa University, Addis Ababa, Ethiopia

**Keywords:** Mastitis, ssGWAS, somatic cell score, dairy cattle, genetic variance

## Abstract

Mastitis, an inflammation of the bovine mammary gland, reduces dairy productivity and poses significant health risks to Ethiopian dairy cattle. This study aimed to identify genomic regions associated with milk somatic cell score (SCS) and estimate its genetic parameters. The dataset included 1647 phenotypic cows, 6964 genotyped animals, and 39,976 single nucleotide polymorphism (SNP) markers. A single-step genome-wide association study (ssGWAS) was conducted, accounting for fixed effects of parity, genomic breed composition, altitude, and lactation stage, and random effects of herd-year-calving-season and permanent environment. Genetic variance using 20 adjacent SNPs sliding windows explaining ≥1% of the total genetic variance were used for candidate genes and quantitative trait loci (QTL) identifications. The estimated heritability of SCS was 0.11 ± 0.06. Genomic regions on BTA 15, 19, and 26 were identified associated with SCS, encompassing 116 genes, including MPP7, MPP8, MMP13, BIRC2, BIRC3, BTRC, SRSF1, and MPO, which are involved in immunity, inflammation, apoptosis, antimicrobial defense, and tissue remodeling. Gene enrichment analysis revealed that the genes are involved collagen catabolic process and IL-17 signaling pathway. These findings provide insights into genomic regions that could be targeted in genomic selection to improve mastitis resistance in Ethiopian dairy cattle.

## Introduction

Dairy production in Ethiopia is predominantly a subsistence-based smallholder system, with a limited number of small to medium-sized commercial farms.^1^ The sector plays a significant role in the economy, contributing to poverty reduction and food security for millions of households.^2^ Various breeding strategies, particularly crossbreeding, have been implemented to improve milk production by introducing superior genetics into the dairy system. Despite these efforts, mastitis, an inflammation-driven disease of the bovine mammary gland, remains a major challenge, negatively affecting animal health, and welfare while causing substantial economic losses.^3^^,^^4^ In Ethiopia, its impact is even more critical because majority of milk is produced under smallholder and peri-urban systems, where poor milking hygiene, limited access to veterinary services, high treatment costs, and lack of awareness about udder health management hinder effective control. Studies have reported a consistently high prevalence of the disease in Ethiopian dairy cattle,^5^^,^^6^ underscoring its significance as a constraint to dairy productivity in the country.

Genome-wide association studies (GWASs) are widely recognized as key tools for identifying quantitative trait loci (QTL), genomic regions, genes, and variants associated with complex and polygenic traits.^7^^,^^8^ Advances in genome sequencing and high-throughput genotyping have enabled GWAS to investigate complex traits in humans and livestock.^9^^,^^10^ In Ethiopian indigenous and crossbred dairy populations, previous genomic studies have primally focused on genetic diversity, admixture, ancestry, and selection signature analyses.^11–13^ However, to date, no genome-wide association studies (GWASs) have been investigated genomic regions and QTL associated with mastitis indicator traits, such as the somatic cell count (SCC), in Ethiopian dairy cattle. Importantly, the SCC is a well-established indicator for mastitis and milk quality,^14^^,^^15^ making it a suitable target trait for applying single-step GWAS (ssGWAS) in dairy cattle.

The ssGWAS methods integrates pedigree, genomic, and phenotypic information into a single framework, reducing additional genotyping costs and providing more accurate genomic breeding value estimates compared to traditional GWAS or Bayesian methods.^16–18^ This approach has become widely used in livestock genetics research for identifying candidate genes and enhancing selection accuracy globally.^7^^,^^19–21^ In African smallholder livestock production systems, the limited availability of phenotypic and pedigree records makes genomic studies of multi-breed and crossbred cattle particularly challenging. Advances in genomic technologies now provide a practical alternatives, with genomic selection already showing promising results in crossbred small-holder cattle populations in East Africa.^22^

Genomic regions and genes associated with mastitis resistance, SCC, and mammary structure have been studied in dairy cattle. The effects of genetic variants on SCC vary depending on breed, population, physiological state, genotyping methods, and GWAS approaches.^7^^,^^19–21^^,^^23^ Although previous studies have identified genes influencing somatic cell score (SCS) and mastitis resistance, these traits are polygenic and generally low heritability (0.04–0.19),^24–27^ which limiting genetic progress through conventional selection. Incorporating molecular marker information can enhance selection for mastitis resistance while also improve milk quality, feed efficiency, fertility, and longevity in dairy cows.^28^^,^^29^ Identifying candidate genes and variants associated with SCC is therefore critical for marker-assisted selection, offering a practical strategy to reduce the burden of this costly disease. Consequently, modern breeding objectives in dairy cattle are increasingly shifting toward a balance between production, health, and longevity traits.^30^

The objective of this study was to identify genomic regions and candidate genes associated with SCS and to estimate genetic parameters across multi-cattle breeds in Ethiopia. By applying the ssGWAS approach, this study aimed to improve the accuracy of genomic prediction for SCS traits in Ethiopia dairy cattle, provides insights into the genetic architecture of SCS, identified potential biomarkers for use in genetic selection programs, and serve as references for future research on mastitis related traits in low-input production systems.

## Materials and methods

### Animals and phenotypes

The phenotypic records for milk somatic cell count (SCC) were collected from cattle of various genetic backgrounds, including Holsteins crosses with local cattle breeds at different admixture levels, Jersey cattle, and indigenous breeds, such as Sheko and Fogera cattle. The milk samples were analyzed via the Lactoscan SCC module of the Lactoscan Combo Machine according to the protocol of the Bulgaria manufacturer,^31^ as described in the detailed sampling and laboratory procedures.^32^ Pedigree information and farm coordinates were retrieved from Ethiopia’s national dairy cattle database under the Africa Asia Dairy Genetic Gains (AADGG) program (https://portal.adgg.ilri.org/; Accessed on 19 June 2023). The dataset comprised 3340 milk SCC records from 1647 cows representing four genetic groups were included in the study. Among them, 33 were of the indigenous breed, 1495 were Holstein Friesians, 84 were 50% crossbreds of Boran and Holstein Friesians, and 35 were Jerseys.

Phenotypic data were quality controlled by removing extreme outliers identified using inter quartile range (IQR) method, where values outside the range Q_3_ + 3*IQR and Q_1_ − 3*IQR were exclude. The non-normally distributed value of SCC were log_e_ transformed to somatic cell score (SCS) using the equation SCS = log_e_ (SCC), following Negussie et al.^33^^,^^34^ and Mrode et al.^35^ Although the conventional SCS formula SCS = log_2_ (SCC/100,000) + 3 proposed by Ali and Shook,^36^ and later modified to SCS = log_2_ (SCC/100) + 3 Wiggans and Shook,^37^ is widely used, the log_e_ transformation provides a comparable normalization effect and effectively stabilizes variance in SCC data.^38^ Normality of the log_e_ transformed SCC was assessed using descriptive statistics (skewness = −0.48 and kurtosis = 3.00), histogram, density plots, and Q–Q plots (as shown in Supplementary Fig. S1), indicated an approximately normal distribution suitable for downstream GWAS analysis. The mean somatic cell count (SCC) was 658,487.3 ± 103,050, corresponding to a mean SCS of 12.38 ± 1.62. SCC values ranged from 1387 to 9,865,244, while the corresponding SCS values ranged from 7.23 to 16.1. In the study herds, dairy cattle were managed under typical smallholder conditions as indicated in Chernet et al.^32^ and disease control relies on traditional practice and occasional veterinary interventions. Summary of the dataset, including the number of phenotyped, genotyped, and pedigree animals, are presented in Table 1.

**Table 1.** Summary of the animals and records used in this study.

Item	Number
Animals with records of SCC	1647
SCC records	3340
Animals with genotypes	6964
Animal with both genotypes and records	642
Animals in pedigree	140,020
Number of sires	1904
Number of dams	19,954
SNP information	39,976
Number of contemporary groups	531

### Genomic data imputation and quality control

A total of 6724 and 2822 animals were initially genotyped using Illumina SNP chips with 54,609 and 95,256 markers, respectively. The raw data were converted to PLINK format via the SNP Suit^39^ and Linux commands, followed by merging and quality control (QC) using the PLINK v1.9.^40^ The raw genotype data were obtained from Ethiopia’s national dairy cattle database under the AADGG project (https://portal.adgg.ilri.org/; Accessed on 19 June 2023). SNPs that did not meet the QC thresholds were filtered out, including those with a minor allele frequency (MAF) ≤5%, a call rate ≤0.95, or individuals with more than 5% missing genotypes. Moreover, SNPs with unspecified physical positions, duplicates, or those located on non-autosomal chromosomes were excluded. After QC and merging, 6964 animals and 39,976 SNPs were retained for the final analysis, this number is reported in Table 1.

### Variance component and genetic parameter estimation

Variance components and genetic parameter were estimated using the single-step genomic best linear unbiased predictions (ssGBLUP) approach implemented in the BLUPF90 software suite.^41^ Given the relatively limited sizes of the dataset, genomic information was incorporated into the estimation of (co)variance components following the method of Dikmen et al.,^42^ which jointly utilizes both pedigree and genomic data. The log_e_ transformed SCC data, pedigrees records, and genotype information were renumbered using the RENUMF90 program from the BLUPF90 family.^41^ Pedigree records were traced back seven generations to ensure accurate genetic relationship estimation. Repeated SCC records per cow were explicitly modeled by including a permanent environmental effect, thereby accounting for correlation among repeated measures from the same cow across different test days, ensuring proper partitioning of non-genetic repeatability.

The final linear mixed model included fixed effects of parity, lactation stage, breed composition, and altitude, along with random effects of herd-year-season of calving and permanent environment of the cow. To ensure adequate representation across subclasses, cows in parity ≥4 were pooled due to the limited number of observations in higher parities. Lactation stage was classified into four classes: early (5–60 days), mid (61–180 days), late (181–305 days), and extended (>305 days in milk). Genomic breed composition was classified into five categories based on exotic blood proportion: <0.25, 0.25–0.50, 0.50–0.75, 0.75–0.875, and above 0.875, as determined by Admixture analysis.^43^ Altitude was divided into three elevation clusters: low (1232–1500 m), mid (1501–2300 m), and high (>2300 m), following Hassen et al.^44^ Herd-year-season of calving (HYCS) was modeled as random effect due to the small numbers of animals within each class, including it as fixed effect could lead to biased estimates. The genetic variation of interest was captured through the random additive genetic effect, ensuring that the genetic effect was not underestimated. The distribution of animals and records across each fixed effect categories are presented in Table 2. Variance components were subsequently estimated using AIREMLF90, based on the single trait animal model described in Equation (1).

**Table 2.** Distribution of animals and records across fixed-effects categories.

Variables	Category	Frequency	Percent	Number of cows
GBC	<0.25	52	1.6	41
	0.25–0.50	334	10.0	159
	0.50–0.75	464	13.9	277
	0.75–0.875	1237	37.0	597
	>0.875	1253	37.5	573
Stage of lactation	Early lactation	409	12.2	224
	Mid lactation	1034	31.0	525
	Late lactation	978	29.3	457
	Extended lactation	919	27.5	441
Parity	1	1003	30.0	517
	2	1085	32.5	512
	3	674	20.2	322
	≥4	578	17.3	296
Altitude	Low (1232–1500 m)	312	9.3	129
	Mid (1501–2300 m)	1702	51.0	913
	High (>2300 m)	1326	39.7	605

### Genome-wide association analysis

A single step genome wide association (ssGWAS) method that integrates phenotype, genotype, and pedigree information improve accuracy of genomic prediction was used for this study.^18^ The single step genomic best linear unbiased prediction (ssGBLUP) approach implemented in the BLUPF90 software suite were used.^41^ The ssGWAS algorithm was applied using various programs from the BLUPF90 software family,^41^ including RENUMF90, AIREMLF90, BLUPF90, and PostGSF90. The ssGWAS analysis was conducted using a single-trait animal model, as indicated in Equation (1).

(1) y=Xβ+Nq+Wp+Za+e

where: y is the vector of milk SCS records; β is the vector of fixed effects including parity, genomic breed composition, altitude, and stage of lactation; q is the vector of random herd-year-season of calving effects; p is the vector of random permanent environmental effects; a is the vector of additive genetic effects; and e is the vector of residual effects. X, N, W, and Z are the incidence matrices relating y to β, q, p, and a, respectively. The random effects were assumed to be normally distributed with zero means. $q∼N(0,N{\sigma_{q}^{2}}^{\;})$; $p∼N(0,I\sigma_{p}^{2})$; $a∼N(0,H\sigma_{a}^{2});e∼N(0,I\sigma_{e}^{2})$, where: $\sigma_{q}^{2}$ is the variance of random herd-year- calving season effects, $\sigma_{p}^{2}$ is the variance of random permanent environmental effects, $\sigma_{a}^{2}$ is the additive genetic variance, $\sigma_{e}^{2}$ is the residual variance, I is the identity matrix, and H is the realized relationship matrix that combines the pedigree and genomic relationships of all genotyped and ungenotyped animals using the ssGBLUP approach.

$$H^{-1}=A^{-1}+[\begin{array}{cc} 0 & 0\\ 0 & G^{-1}-A_{22}^{-1} \end{array}\;]$$

where $A^{-1}$ is the inverse of the pedigree-based relationship matrix for all animals, $G^{-1}$ is the inverse of the genomic relationship matrix for genotyped animals and $A_{22}^{-1}$ is the inverse of pedigree based relationship matrix for genotyped animals.^45^

The *G* matrix was computed following^46^ using the formula: *G* = *ZDZ*′*q*, where *Z* is a matrix of gene content adjusted for allele frequencies, *D* is a diagonal matrix with the inverse of expected SNP variance (initially *D* =* I*), and *q* is a normalizing factor, which can be derived from SNP frequencies. SNP effects were determined using the postGSf90 program.^45^ The ssGWAS was run for five compression iteration.^17^ The percentage of the genetic variance explained by the *i*^th^ SNP window was calculated according to Wang et al.^17^

$$\frac{\text{Va}r_{\left(\text{ai}\right)}}{\sigma_{a}^{2}}\times 100\text{\%}=\frac{\text{Var}\left(\sum_{j=1}^{20}Z_{j}U_{j}\right)}{\sigma_{a}^{2}}\times 100\text{\%}$$

Where ai is genetic value of the *i*^th^ region that consists of 20 adjacent SNPs, $\sigma_{a}^{2}$ is the total genetic variance, $Z_{j}$ is vector of gene content of the *j*^th^ SNP for all individuals, and $U_{j}$ is marker effect of the *j*^th^ SNP within the *i*^th^ region.

The challenge of window-based GWAS is the absence of a universal standard for hypothesis testing,^47^ it is quite a common procedure in genetic studies. Window-based GWAS may use different window types (distinct or sliding windows) and variable window sizes (defined as the number of SNP or the number of base pairs). Typically, significance is declared based on the proportion of additive genetic variance explained by individual windows.^47^ However, there is no consensus on the optimal window size or the appropriate threshold for explained variance.^47^ In our study, a threshold of ≥1% additive genetic variance explained by 20 adjacent SNPs sliding windows was used to identify candidate genomic regions for gene annotation. Variation across different window sizes (5, 10, 15, 20, 30, and 50 SNPs) were diagnosed, we observed variability in number and size of peak regions explaining genetic variance, as shown in the Supplementary Figs. S2–S6. Smaller windows (5–15 SNPs) produce numerous narrow peaks, but several appeared unstable. In contrast, larger windows (30–50 SNPs) tended to merged adjacent peaks and diluted the variance signals. The 20 SNPs windows consistently captured the main genomic regions explaining variance in the studied traits, providing an optimal balance between mapping resolution and noise reduction. For this reason, we present these results in detail. Genomic windows were ultimately defined using 20 adjacent SNPs to minimize noise from smaller windows while avoiding the loss of broader signals that may be masked when using excessively large windows, as recommendation by Fragomeni et al.^48^ and Oliveira et al.^20^ The threshold criteria is consistent with previous ssGWAS studies in livestock, where similar or lower thresholds have been applied, for example, 0.31,^20^^,^^49^ 0.5,^7^^,^^50^^,^^51^ and 1%.^52^^,^^53^ Window-based variance approach captures the cumulative genetic contribution of adjacent SNPs and is particularly suitable for complex traits. Although SNP-level *p*-value are available in the BLUPF90 packages, we focused on windo-based varaince approach to detect meaningful genomic regions under practical field conditions. Genes located within these regions were considered putative candidates, reflecting the cumulative effects of multiple SNPs rather than relying solely on individual SNP *p*-values. A Manhattan plot illustrating these genomic windows and variance explained by SNP windows were generated in base R (version 4.2.2) using standard plotting functions (plot, abline, axis)^54^ to visualize the variance explained by SNP windows across chromosomes. Because window-based ssGWAS does not produce standard *p*-values, genomic inflation factors (*λ*) and QQ plots are not directly applicable, and therefore not reported in several studies,^17^^,^^55^ including ours.

### Functional annotation and enrichment analyses

Based on significant SNPs windows using the starting and ending coordinates of the genomic regions, gene annotations were performed using Biomart package in R (version 4.2.2) from the Ensembl BioMart database (www.ensembl.org/biomart/; accessed on 4^th^ November 2024^56^) and also from the NCBI Genome Data Viewer for domestic cattle (*Bos taurus*) (https://www.ncbi.nlm.nih.gov/gdv?org=bos-taurus&group=bovinae; accessed on 4^th^ November 2024), the background gene set was defined as all annotated genes within the basis of the latest *Bos taurus* genome assembly of ARS-UCD 2.0 genome to minimize bias.

For functional characterization of candidate genes was performed using the Database for Annotation, Visualization and Integrated Discovery (DAVID, v2023q4), a widely used bioinformatics resource that provides functional annotation tools for large gene lists.^57^^,^^58^ DAVID generates annotation tables, charts, and clusters to facilitate the interpretation of biological meaning. A Benjamini-Hochberg adjusted significant threshold of *p* ≤ 0.05 was applied to identify enriched Gene Ontology (GO) terms for biological processes, molecular functions, and cellular components, as well as Kyoto Encyclopedia of Genes and Genomes (KEGG) pathways. To prioritize functionally relevant genes, we highlighted those located within the top SNP windows explaining ≥1.0% of additive genetic variance and those previously reported to be associated with SCS, mastitis, immune responses, or lactation traits. Protein-protein interaction (PPI) networks of the candidate genes were generated using STRING v12.0 with a minimum interaction score of 0.4 (https://string-db.org/; accessed on 4^th^ November 2024). The STRING database integrates experimentally validated and predicted interaction, including both physical and functional associations, to visualize functional relationships.^59^

#### QTL identification

SNP windows explaining more than 1.0% of total additive genetic variance were further used to identify QTLs. The start and end positions of these windows were quired against the animal quantitative trait loci (QTL) database (Animal QTLdb) targeting cattle QTLdb (release 57, accessed on 15^th^ August 2025^60^) to retrieve previously reported QTLs within the regions. Identified QTLs were then catalogued based on their associations and causative effects.

## Results

### Variance component and genetic parameter estimates

The estimates of variance components for the SCS are presented in Table 3. The heritability estimate for the SCS was 0.11 ± 0.06, with the repeatability estimate was 0.38 ± 0.23, indicating that the SCS has relatively low heritability and is largely influenced by environmental factors. The estimated variance of the permanent environmental effect was 0.70 ± 0.15, demonstrating that persistent non-genetic factors contribute substantially to the trait. Along with the random herd-year-season of calving (HYSC) variance of 0.39 ± 0.06, this highlights that both between group environmental effects and within-animal consistent environmental influence play important roles in the observed variation of SCS. These results suggest that both persistent non-genetic effects and HYSC contribute considerably to SCS variation, while the low heritability implies that genetic selection alone would lead to slow improvement, emphasizing the importance of management interventions.

**Table 3.** Variance component and heritability estimations.

Variance	Value ± *SE*
Permanent environmental effect	0.70 ± 0.15
Random herd year season of calving effect	0.39 ± 0.06
Additive genetic effect	0.27 ± 0.15
Residual variance	1.19 ± 0.04
Total phenotype	2.55 ± 0.22
Heritability	0.11 ± 0.06
Repeatability	0.38 ± 0.23

### Candidate genes and genomic regions associated with the SCS

**Figure 1.**
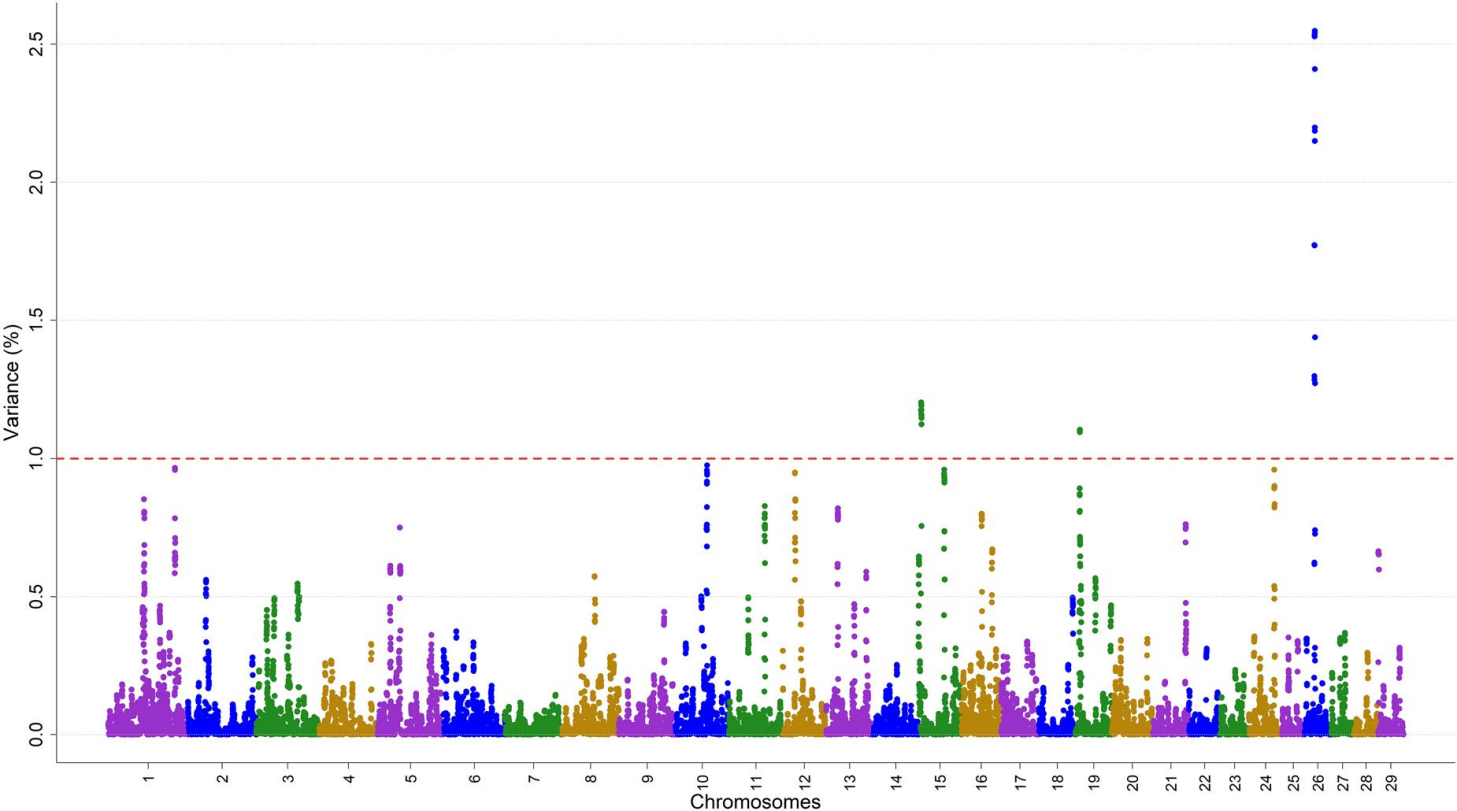
Manhattan plot of the proportion of additive genetic variance (%) explained by moving windows of 20 adjacent SNPs for milk SCS in Ethiopian dairy cattle. Each dot denotes a window. The red broken line represents 1% of genetic variance.

Genomic regions associated with the SCS were identified in three *Bos taurus* autosome chromosomes: BTA15, BTA19, and BTA26. The specific windows linked to SCS, along with their corresponding genes, are summarized in Table 4. On BTA15, the genomic region located between 5.7 and 7.0 megabase (Mb) was associated with SCS, spanning 1.3 Mb and contains 25 genes, including MMP13, BIRC2, BIRC3, and YAP1. On BTA19, the 1.1 Mb region between 8.2 and 9.3 Mb harbored 31 genes, such as MPO, LPO, and SRSF1. On BTA26, the genomic region located between 21.4 and 23.3 Mb, with 1.9 Mb region contains 60 genes, including BTRC and HIF1AN. The ranges of variance contributions for the chromosomes identified were Chr15 (0.00057–1.20217), Chr19 (0.00017–1.10444), and Chr26 (0.00002–2.54737). Some of the annotated genes were not well characterized and the summary is provided in Supplementary Table 1. Figure 1 shows a Manhattan plot illustrating the proportion of additive genetic variance explained by 20-SNP sliding windows for milk SCS.

**Table 4.** SNP windows that explained ≥1% of the genetic variance in the milk somatic score and their annotated genes.

Chr^a^	SNP window^b^		Var (%)^c^	Annotated gene^d^
	Start (bp)	Stop (bp)		
15	5,674,820	7,033,930	1.20	MMP13, MMP12, MMP3, MMP1, MMP8, MMP27, MMP20, MMP7, TMEM123, BIRC2, BIRC3, YAP1, CFAP300, CEP126, ANGPTL5
19	8,157,946	9,281,946	1.10	MSI2, MIR378D, CCDC182, MRPS23, CUEDC1, VEZF1, SRSF1, DYNLL2, MKS1, LPO, MPO, TSPOAP1
26	21,409,429	23,307,206	2.55	SEC31B, NDUFB8, HIF1AN, PAX2, TRNAG-UCC, SLF2, SEMA4G, MRPL43, TWNK, LZTS2, PDZD7, SFXN3, KAZALD1, TLX1, LBX1, BTRC, POLL, DPCD, FBXW4, FGF8, NPM3, OGA, KCNIP2, ARMH3, HPS6, LDB1, PPRC1, NOLC1, TRNAR-CCU, ELOVL3, PITX3, GBF1, NFKB2, PSD, FBXL15, CUEDC2, MIR146B, MFSD13A, ACTR1A, SUFU, TRIM8, ARL3

^a^ Chr: chromosome.

^b^ Starting and ending coordinates of the window base pairs, with 20 consecutive SNPs.

^c^ Var: % genetic variance explained by the SNPs within the window.

^d^ Ensembl symbol of annotated genes using the Bos taurus ARS-UCD2.0 assembly (http://www.ensembl.org/biomart/martview) and NCBI Genome Data Viewer for domestic cattle (*Bos taurus*)—(https://www.ncbi.nlm.nih.gov/gdv?org=bos-taurus&group=bovinae).

### Enrichment analysis of candidate genes

The GO enrichment analyses highlighted that the identified candidate genes involved in collagen catabolic process, extracellular matrix organization, and proteolysis were the most enriched biological processes (Table 5). Additionally, KEGG pathway analyses revealed two key pathways associated with the candidate genes influencing SCS and udder health in cows, namely, the interleukin 17 (IL-17) signaling pathway and transcriptional misregulation in cancer (Table 6).

**Table 5.** List of significantly enriched GO terms associated with biological process, cellular components, and molecular functions of the identified candidate genes.

GO term description	Count	Genes	Benjamini
Biological process			
GO:0030574∼collagen catabolic process	9	*LOC112441463*, MMP1, MMP12, MMP13, MMP20, MMP27, MMP3, MMP7, MMP8	7.22 × 10^−12^
GO:0030198∼extracellular matrix organization	10	KAZALD1, LOC112441463, MMP1, MMP12, MMP13, MMP20, MMP27, MMP3, MMP7, MMP8	4.28 × 10^−8^
GO:0006508∼proteolysis	10	BTRC, LOC112441463, MMP1, MMP12, MMP13, MMP20, MMP27, MMP3, MMP7, MMP8	0.013
Molecular function			
GO:0004222∼metalloendopeptidase activity	9	LOC112441463, MMP1, MMP12, MMP13, MMP20, MMP27, MMP3, MMP7, MMP8	6.40 × 10^−7^
GO:0008270∼zinc ion binding	11	BIRC2, HIF1AN, LOC112441463, MMP1, MMP12, MMP13, MMP20, MMP27, MMP3, MMP7, MMP8	0.005
Cellular components			
GO:0031012∼extracellular matrix	9	LOC112441463, MMP1, MMP12, MMP13, MMP20, MMP27, MMP3, MMP7, MMP8	4.18 × 10^−12^
GO:0005615∼extracellular space	16	KAZALD1, ANGPTL5, LOC788751, FGF8, LOC112441463, LPO, MMP1, MMP12, MMP13, MMP20, MMP27, MMP3, MMP7, MMP8, MPO, SEMA4G	0.003

**Table 6.** Significantly enriched KEGG pathways associated with identified candidate genes.

KEGG pathway	Count	Genes	Benjamini
bta04657:IL-17 signaling pathway	5	LOC112441463, MMP1, MMP13, MMP3, SRSF1	0.044
bta05202: Transcriptional misregulation in cancer	6	LDB1, TLX1, BIRC2, BIRC3, MMP3, MPO	0.045

### A protein-protein interaction network

**Figure 2.**
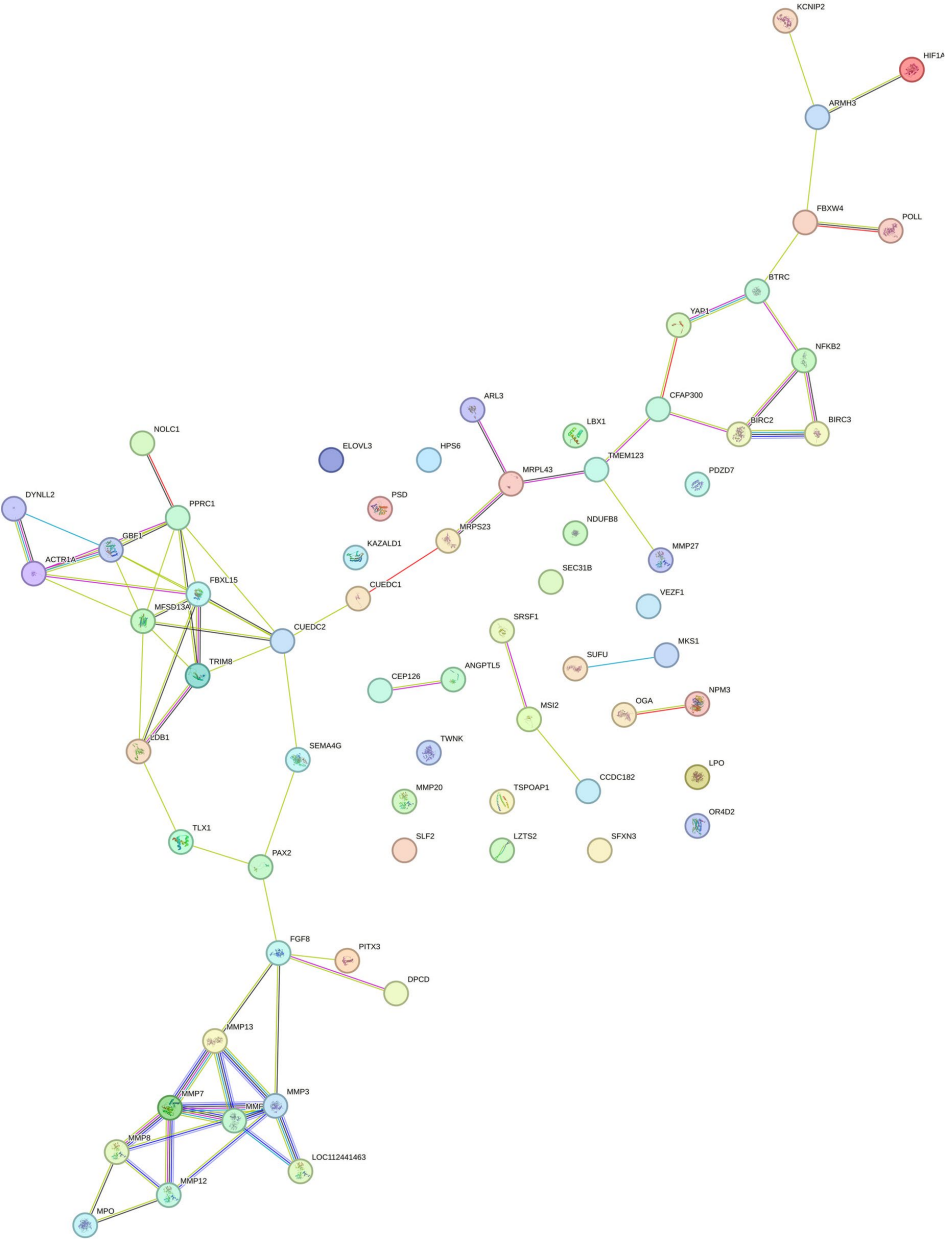
Gene network protein-protein interaction diagram.

The protein-protein interaction (PPI) network analysis revealed meaningful biological interactions among candidate genes (Fig. 2). The network comprised 67 proteins connected by 72 edge-observed interactions, with an average of 2.17 interactions per protein. The local clustering coefficient of 0.439 indicates moderate clustering, suggesting that while proteins form functional groups, the overall connectivity within the network is not very dense. Importantly, the network showed a higher number of interactions than expected by chance (12 edge), with a PPI enrichment *p*-value of 1.0 × 10^−16^, demonstrating highly significant enrichment for functional associations. This indicates that the candidate genes are likely involved in shared biological pathways or process rather than interacting randomly, supporting their potential functional relevance in the traits under study.

### QTL mapping

QTLs regions were identified in the significant genomic regions of chromosome 15, 19, and 26 found associated with somatic cell score. However, these loci are linked to a wide range of traits, including milk production traits, milk composition, growth and body conformation, fertility, feed efficiency, heat tolerance, and disease resistance, as detailed in Supplementary Table 2. Among the detected QTLs, some of them directly associated with SCS, for instance QTL: 238,553 and QTL: 10,446 (SCS), QTL: 4648 (udder attachment), and QTL: 106,743 (udder swelling score).

## Discussion

This study is, to our knowledge, the first to identify genomic regions and estimate the genetic parameters related to milk somatic cell scores in Ethiopian multi-breed dairy cattle. The heritability estimated in our study was higher than those reported by Ablondi et al.^24^ (*h*^2^ = 0.06) and Kheirabadi and Razmkabir^61^ (*h*^2^ = 0.03). Similarly, a study in Finland reported that the heritability of SCC in Ayrshire cows ranged from 0.09 to 0.12 and 0.08 to 0.10 in Holstein–Friesian cows for the first and second lactations, respectively.^25^ In contrast, Buaban et al.^7^ reported a higher heritability estimate of 0.32 for average SCS, while Sabedot et al.^62^ reported heritability estimates of 0.21 for Brazilian Jersey herds. In Iranian Holstein dairy cows, heritability estimates for SCS ranged from 0.15 to 0.18.^63^ These variations across studies reflect difference in breed, lactation, management, and environmental conditions. Our results indicate low heritability but moderate repeatability for SCS, consistent with those previous reports, highlighting the influence of both genetic and persistent environmental factors.

The significant permanent environmental variance underscores the role of non-genetic effects. Modeling herd-year-season of calving (HYCS) as random effect was appropriate given small number of animals per subclass in our data. Using HYCS as fixed effect under such condition could lead unstable estimates. Although the random modeling may slightly reduce precision, it allowed for a more reliable partitioning of variance and prevents overparameterization. The genetic variation of SCS remains adequately captured through the random additive genetic effect. The estimated HYCS variance also contributed notably to the observed phenotypic variation. Under smallholder conditions, where management practices can vary widely, these findings emphasize the need for integrated strategies to improve udder health. Effective reduction of SCS requires a combination of genetic selection and consistent management interventions, including improved hygiene, milking practices, and nutrition.

Several significant candidate genes from the matrix metalloproteinase (MMP) family were identified on BTA 15, including MMP1, MMP3, MMP7, MMP8, MMP12, MMP13, MMP20, and MMP27. MMPs are zinc-dependent proteolytic enzymes that involved in extracellular matrix (ECM) tissue remodeling and regulation of innate immunity through their effects on proinflammatory cytokines, chemokines, and other immune-related proteins.^64^^,^^65^ Their role in facilitating the migration of inflammatory cells to infection sites that variation in these genes could directly influence SCS and susceptibility to mastitis. However, their dysregulation can lead to excessive tissue destruction, increasing the risk of complications, such as mastitis.^66^ Similar to our findings, an association between MMP7 and the SCS has been reported in Romanian dairy cattle;^67^ MMP7 expression was significantly higher in cows infected with coagulase positive *Staphylococci* than healthy cows.^64^ Similarly, MMP8 expression has been reported to be higher in cows with clinical mastitis than in cows with subclinical mastitis or healthy cows.^68^ In agreement with our findings, MMP13 has been identified as a candidate gene associated with the SCS in the Slovenian Holstein Friesian population.^69^ The identification of these MMP genes in Ethiopian multi-breed cattle suggests that similar immune and inflammatory mechanism may contribute to variation in SCS under smallholder management conditions.

Bovine myeloperoxidase (MPO) is a lysosomal peroxidase enzyme stored in the azurophilic granules of neutrophils, playing a key role in the innate immune system and first-line defense against pathogens in cattle.^70^ In dairy cows, elevated MPO levels in milk neutrophils and macrophages have been reported in mastitis cow than the healthy one, reflecting its involvement in intramammary infections.^71^ It contributes to neutrophil function, including the release of neutrophil extracellular traps (NETs), which help contain infection and limit tissue damage.^71^ MPO in somatic cells has been suggested as a biomarker for measuring mammary gland inflammation in buffalo udders.^72^ The identification of MPO among candidate genes in our study suggests that variation in this gene may influence SCS and susceptabilty to mastitis. MPO in somatic cells established as a biomarker for measuring mammary gland inflammation in cattle and buffalo^72^^,^^73^ indicates that MPO could serve as traget for genomic selection aimed at improving udder health.

In our study, BIRC2 and BIRC3, members of the baculoviral inhibitor of apoptosis protein repeat (BIRC) family, were identified as candidate genes influencing SCS. These genes regulate apoptosis, inflammatory signaling, cell invasion, and cell proliferation.^74^^,^^75^ Transcriptome analysis have shown that BIRC2 and BIRC3 are upregulated in mammary glands infected with *Staphylococcus aureus* response, suggesting their role in immune response to mastitis.^76^^,^^77^ By inhibiting caspase protease activity, BIRC3 prevents excessive cell death, which may help maintain lactation during infection.^75^ Furthermore, in our findings, we found that SRSF1 (serine/arginine-rich splicing factor 1) was identified as a potential candidate gene influencing SCS. It encodes a splicing regulatory protein involved in apoptosis, cell proliferation, and alternative splicing, which can affect gene expression and cellular function.^78^^,^^79^ Although much of the literature on SRSF1 focuses on its role in cancer,^80^ its regulatory functions in apoptosis and cell proliferation suggest it may influence immune response and mammary gland health in cattle. Variation in SRSF1 could therefore contribute to differences in SCS, reflecting the cow’s ability to respond to intramammary infections.

Hypoxia-inducible factor 1-alpha subunit suppressor (HIF1AN), a component of hypoxia-inducible factor (HIF) pathways identified as potential candidate gene associated with SCS in our study. It modulates the stability and activity of hypoxia-inducible factor 1-alpha (HIF-1α), which plays a central role in immune cell metabolism and function.^81^ While much studies have shown that HIF1AN gene expression is downregulated in various cancers, including breast cancer, thyroid cancer, prostate adenocarcinoma, and uterine corpus endometrial carcinoma.^81^ Its role in immune regulation suggests that variation in this gene may influence SCS and cow’s ability to respond to mammary infection. In addition to its relation with SCS, HIF1AN has reported to have an association with milk production traits, particularly milk fat content in Holstein cattle^82^ and fatty acid composition in dual-purpose Belgian Blue cows.^83^ HIF1AN identification in our study implies a potential pleiotropic effect, where it may influence both milk composition and immune functions in the mammary gland.

Another notable gene identified was beta-transducin repeat containing E3 ubiquitin protein ligase (BTRC) which is involved in protein homeostasis and cellular regulation, and its activity may influence tissue remodeling and immune response in the mammary gland.^84^ In agreement to our results, BTRC is previously reported having an associated with milk production traits and udder characteristics, including mammary gland development and duct morphogenesis in dairy cattle.^85^^,^^86^ In addition to immune-related genes we have found, several candidate genes, such as BTRC, TLX1, KAZALD1, and LDB1, associated with milk composition traits including milk fat percentage.^82^^,^^83^ The presence of these genes within SCS-associated genomic regions highlights the potential for pleiotropic effects, where the same genomic regions may influence both udder health and milk production traits. In our study, we also identified QTLs associated with SCS. However, the identified regions contains QTLs linked with various traits, including milk production, milk composition, disease resistance, reproduction, and others traits which also confirm the pleotropic effects of the identified genomic.^42^^,^^50^^,^^87^ The consistence between QTLs identified in this study and those reported previously highlights the importance of these genomic regions for targeted genomic selection. Some genomic regions contained gens that are not well characterized (e.g., LOC112441463 and LOC788751), indicating that further functional studies are needed to clarify their role in SCS.

Although only a limited number of animals had phenotypic records, reflecting typical data constraints in developing country field conditions, the dataset was sufficient to estimate meaningful genetic parameters and identify relevant candidate genes. Because the ssGWAS approach we implemented helped mitigate the impact of missing data by leveraging genomic relationships among genotyped and non-genotyped animals.^18^ Studies with comparable sample sizes have been undertaken previously,^50^^,^^69^ supporting the reliability of our findings. While shallow pedigree depth, unbalanced breed composition, and possible population structure may have had also some influence, genomic selection has shown positive results under similar condition in crossbred smallholder cattle populations in East Africa.^22^ In Ethiopia, where mastitis prevalence is high and reducing milk quality and affecting income of farmers, such study that integrate genomic and phenotypic data provide valuable insight for tackling these challenges. Importantly, the availability of diverse indigenous and admixed cattle in the country may serve as a unique allelic diversity for resistance to udder health problems.^11^ We have conducted GWAS for traits with low heritability using a limited number of phenotyped animals; however, to address these limitations , it is crucial to validate the usefulness of the identified QTL.^42^ Strengthening pedigree and performance recordings, alongside incorporating the identified genomic regions and QTL-linked SNP windows into SNP panels and genomic evaluations, would provide a practical pathway to improve mastitis resistance in smallholder systems. Ultimately, validation in larger Ethiopia reference populations will be necessary before routinely implementation, but the overlap with known candidate genes and QTLs provides valuable insight into genetic architecture of SCS and confidence that these regions represent strong candidate for practical genomic selections tools tailored to Ethiopia production systems.

## Conclusions

In this study, we estimated genetic parameters and identified several candidate genes associated with SCS in dairy cattle. The low heritability and moderate repeatability illustrated that the SCS is mainly influenced by both genetic and environmental factors. The identified candidate genes (MPP7, MPP8, MMP13, BIRC2, BIRC3, BTRC, SRSF1, and MPO) and QTLs were involved in immunity, antimicrobial defense, inflammation, apoptosis, and tissue remodeling, most of which overlapped with previously reported regions, emphasizing their biological association with the studied trait. In Ethiopia, where mastitis prevalence is high, integrating the results of these findings along with improved recording schemes and management practices offers a promising pathway to reduce SCC and enhance milk quality. However, functional validation and replication in larger datasets are required before these markers can be reliably implemented in genomic selection programs.

## Supplementary Material

Supplementary_Figure_S1.docx

Supplementary_Figures_S2_S6.docx

Supplementary _Table_1_2.docx

## Data Availability

The datasets generated and analyzed during this study are available from the corresponding author upon reasonable request, without undue reservation.

## References

[1] Gebreyohanes G, Yilma Z, Moyo S, Mwai OA. *Dairy industry development in Ethiopia: Current status, major challenges and potential interventions for improvement*. ILRI. Position Paper. Nairobi: ILRI; 2021.

[2] CSA. *Agricultural Sample Survey 2021/22 [2014 E. C.]. Report on livestock and livestock characteristics (private peasant holdings). Volume 2 Statistical bulletin 594*. Addis Ababa: Federal Democratic Republic of Ethiopia Central Statistical Authority (CSA); 2022.

[3] Brajnik Z, Ogorevc J. Candidate genes for mastitis resistance in dairy cattle: a data integration approach. *J Anim Sci Biotechnol*. 2023;14(1):10. 36759924 10.1186/s40104-022-00821-0 PMC9912691

[4] Naserkheil M, Ghafouri F, Zakizadeh S, et al. Multi-omics integration and network analysis reveal potential hub genes and genetic mechanisms regulating bovine mastitis. *Curr Issues Mol Biol*. 2022;44(1):309–328. 35723402 10.3390/cimb44010023 PMC8928958

[5] Getaneh AM, Gebremedhin EZ. Meta-analysis of the prevalence of mastitis and associated risk factors in dairy cattle in Ethiopia. *Trop Anim Health Prod*. 2017;49(4):697–705. 28185209 10.1007/s11250-017-1246-3

[6] Girma A, Tamir D. Prevalence of bovine mastitis and its associated risk factors among dairy cows in Ethiopia during 2005–2022: a systematic review and meta-analysis. *Vet Med Int*. 2022;2022:7775197. 36164492 10.1155/2022/7775197 PMC9509276

[7] Buaban S, Lengnudum K, Boonkum W, Phakdeedindan P. Genome-wide association study on milk production and somatic cell score for Thai dairy cattle using weighted single-step approach with random regression test-day model. *J Dairy Sci*. 2022;105(1):468–494. 34756438 10.3168/jds.2020-19826

[8] Sahana G, Cai Z, Sanchez MP, Bouwman AC, Boichard D. Invited review: Good practices in genome-wide association studies to identify candidate sequence variants in dairy cattle. *J. Dairy Sci*. 2023;106(8): 5218–5241. doi: 10.3168/jds.2022-22694. 37349208

[9] Jiang L, Liu J, Sun D, et al. Genome wide association studies for milk production traits in Chinese Holstein population. *PLOS One*. 2010;5(10):e13661. 21048968 10.1371/journal.pone.0013661 PMC2965099

[10] Stranger BE, Stahl EA, Raj T. Progress and promise of genome-wide association studies for human complex trait genetics. *Genetics*. 2011;187(2):367–383. 21115973 10.1534/genetics.110.120907 PMC3030483

[11] Gebrehiwot NZ, Strucken EM, Aliloo H, Marshall K, Gibson JP. The patterns of admixture, divergence, and ancestry of African cattle populations determined from genome-wide SNP data. *BMC Genomics.* 2020;21(1):869. 33287702 10.1186/s12864-020-07270-x PMC7720612

[12] Kim K, Kwon T, Dessie T, et al. The mosaic genome of indigenous African cattle as a unique genetic resource for African pastoralism. *Nat Genet*. 2020;52(10):1099–1110. 32989325 10.1038/s41588-020-0694-2

[13] Terefe E, Belay G, Tijjani A, Han J, Hanotte O. Whole genome resequencing reveals genetic diversity and selection signatures of Ethiopian indigenous cattle adapted to local environments. Diversity. 2023;15(4):540. doi: 10.3390/d15040540.

[14] IDF. Guidelines for the use and interpretation of bovine milk somatic cell count. *Bull IDF*. 2013;466:14.

[15] Rainard P, Foucras G, Boichard D, Rupp R. Invited review: Low milk somatic cell count and susceptibility to mastitis. *J Dairy Sci*. 2018;101(8):6703–6714. 29803421 10.3168/jds.2018-14593

[16] Lourenco D, Legarra A, Tsuruta S, Masuda Y, Aguilar I, Misztal I. Single-step genomic evaluations from theory to practice: using SNP chips and sequence data in blupf90. *Genes*. 2020;11(7):790. 32674271 10.3390/genes11070790 PMC7397237

[17] Wang H, Misztal I, Aguilar I, et al. Genome-wide association mapping including phenotypes from relatives without genotypes in a single-step (ssGWAS) for 6-week body weight in broiler chickens. *Front Genet*. 2014;5:134. 24904635 10.3389/fgene.2014.00134 PMC4033036

[18] Wang H, Misztal I, Aguilar I, Legarra A, Muir WM. Genome-wide association mapping including phenotypes from relatives without genotypes. *Genet Res*. 2012;94(2):73–83. 10.1017/S0016672312000274 22624567

[19] Nani JP, Raschia MA, Poli MA, Calvinho LF, Amadio AF. Genome-wide association study for somatic cell score in Argentinean dairy cattle. *Livest Sci*. 2015;175:1–9. 10.1007/s13353-015-0278-5 25783851

[20] Oliveira HR, Cant JP, Brito LF, et al. Genome-wide association for milk production traits and somatic cell score in different lactation stages of Ayrshire, Holstein, and Jersey dairy cattle. *J Dairy Sci*. 2019;102(9):8159–8174. 31301836 10.3168/jds.2019-16451

[21] Welderufael BG, Løvendahl P, de Koning DJ, Janss LLG, Fikse WF. Genome-wide association study for susceptibility to and recoverability from mastitis in Danish Holstein cows. *Front Genet*. 2018;9:141. 29755506 10.3389/fgene.2018.00141 PMC5932407

[22] Brown A, Ojango J, Gibson J, Coffey M, Okeyo M, Mrode R. Short communication: Genomic selection in a crossbred cattle population using data from the Dairy Genetics East Africa Project. *J Dairy Sci*. 2016;99(9):7308–7312. 27423951 10.3168/jds.2016-11083

[23] Iung LHS, Petrini J, Ramírez-Díaz J, et al. Genome-wide association study for milk production traits in a Brazilian Holstein population. *J Dairy Sci*. 2019;102(6):5305–5314. 30904307 10.3168/jds.2018-14811

[24] Ablondi M, Summer A, Stocco G, et al. Heritability and genetic correlations of total and differential somatic cell count with milk yield and composition traits in Italian Simmental cows. *J Dairy Sci*. 2023;106(12):9071–9077. 37641255 10.3168/jds.2023-23639

[25] Koivula M, Negussie E, Mäntysaari EA. Genetic parameters for test-day somatic cell count at different lactation stages of Finnish dairy cattle. *Livest Prod Sci*. 2004;90(2–3):145–157.

[26] Liu D, Xu Z, Zhao W, et al. Genetic parameters and genome-wide association for milk production traits and somatic cell score in different lactation stages of Shanghai Holstein population. *Front Genet*. 2022;13:940650. 36134029 10.3389/fgene.2022.940650 PMC9483179

[27] Romano GS, Pinto LFB, Valloto AA, Horst JA, Pedrosa VB. Genetic parameters between somatic cell score and production traits for Holstein cattle in southern Brazil. *Rev Colom Cienc Pecua*. 2020;33(1):60–70.

[28] Martin P, Barkema HW, Brito LF, Narayana SG, Miglior F. Symposium review: Novel strategies to genetically improve mastitis resistance in dairy cattle. *J. Dairy Sci*. 2018;101(3):2724–2736. 29331471 10.3168/jds.2017-13554

[29] Potter TL, Arndt C, Hristov AN. Short communication: increased somatic cell count is associated with milk loss and reduced feed efficiency in lactating dairy cows. *J Dairy Sci*. 2018;101(10):9510–9515. 30077458 10.3168/jds.2017-14062

[30] Miglior F, Fleming A, Malchiodi F, Brito LF, Martin P, Baes CF. A 100-year review: identification and genetic selection of economically important traits in dairy cattle. *J Dairy Sci*. 2017;100(12):10251–10271. 29153164 10.3168/jds.2017-12968

[31] Milkotronic Ltd. LACTOSCAN COMBO operation manual. Bulgaria; 2017:0–213. http://lactoscan.com/editor/ufo/manuals/COMBO/IM_COMBO_EN.pdf.

[32] Chernet TF, Mwai O, Meseret S, et al. Milk somatic cell count, composition and yield of multi-breed dairy cattle in Ethiopia. *Cogent Food Agric*. 2024;10(1):1–15.

[33] Negussie E, Koivula M, Mäntysaari EA. Genetic parameters and single versus multi-trait evaluation of udder health traits. *Acta Agric Scand A Anim Sci*. 2006;56(2):73–82.

[34] Negussie E, Koivula M, Mäntysaari EA, Lidauer M. Genetic evaluation of somatic cell score in dairy cattle considering first and later lactations as two different but correlated traits. *J Anim Breed Genet*. 2006;123(4):224–238. 16882089 10.1111/j.1439-0388.2006.00594.x

[35] Mrode R, Pritchard T, Coffey M, Wall E. Joint estimation of genetic parameters for test-day somatic cell count and mastitis in the United Kingdom. *J Dairy Sci*. 2012;95(8):4618–4628. 22818477 10.3168/jds.2011-4971

[36] Ali AKA, Shook GE. An optimum transformation for somatic cell concentration in milk. *J. Dairy Sci*. 1980;63(3):487–490.

[37] Wiggans GR, Shook GE. A lactation measure of somatic cell count. *J Dairy Sci*. 1987;70(12):2666–2672. 3448115 10.3168/jds.S0022-0302(87)80337-5

[38] Mrode RA, Swanson GJT, Winters MS. Genetic parameters and evaluations for somatic cell counts and its relationship with production and type traits in some dairy breeds in the United Kingdom. *Anim Sci*. 1998;66(3):569–576.

[39] Nicolazzi E, Marras G, Stella A. SNPConvert: SNP array standardization and integration in livestock species. *Microarrays*. 2016;5(2):17. 27600083 10.3390/microarrays5020017 PMC5003493

[40] Purcell S, Neale B, Todd-Brown K, et al. PLINK: A tool set for whole-genome association and population-based linkage analyses. *Am J Hum Genet*. 2007;81(3):559–575. 17701901 10.1086/519795 PMC1950838

[41] Misztal I, Tsuruta S, Lourenco D, et al. Manual for BLUPF90 family of programs; 2022.

[42] Dikmen S, Cole JB, Null DJ, Hansen PJ. Genome-wide association mapping for identification of quantitative trait loci for rectal temperature during heat stress in Holstein cattle. *PLOS One*. 2013;8(7):e69202. 23935954 10.1371/journal.pone.0069202 PMC3720646

[43] Alexander DH, Novembre J, Lange K. Fast model-based estimation of ancestry in unrelated individuals. *Genome Res*. 2009;19(9):1655–1664. 19648217 10.1101/gr.094052.109 PMC2752134

[44] Hassen A, Ebro A, Kurtu M, Treydte AC. Livestock feed resources utilization and management as influenced by altitude in the Central Highlands of Ethiopia. *Livest Res Rural Dev*. 2010;22:229. http://www.lrrd.org/lrrd22/12/hass22229.htm.

[45] Aguilar I, Misztal I, Legarra A, Tsuruta S. Efficient computation of the genomic relationship matrix and other matrices used in single-step evaluation. *J Anim Breed Genet*. 2011;128(6):422–428. 22059575 10.1111/j.1439-0388.2010.00912.x

[46] VanRaden PM. Efficient methods to compute genomic predictions. *J Dairy Sci*. 2008;91(11):4414–4423. 18946147 10.3168/jds.2007-0980

[47] Aguilar I, Legarra A, Cardoso F, Masuda Y, Lourenco D, Misztal I. Frequentist *p*-values for large-scale-single step genome-wide association, with an application to birth weight in American Angus cattle. *Genet Sel Evol*. 2019;51(1):28. 31221101 10.1186/s12711-019-0469-3 PMC6584984

[48] Fragomeni BO, Misztal I, Lourenco DL, Aguilar I, Okimoto R, Muir WM. Changes in variance explained by top SNP windows over generations for three traits in broiler chicken. *Front Genet*. 2014;5:332. 25324857 10.3389/fgene.2014.00332 PMC4181244

[49] Raschia MA, Nani JP, Carignano HA, Amadio AF, Maizon DO, Poli MA. Weighted single-step genome-wide association analyses for milk traits in Holstein and Holstein x Jersey crossbred dairy cattle. *Livest Sci*. 2020;242:104294.

[50] Devani K, Plastow G, Orsel K, Valente TS. Genome-wide association study for mammary structure in Canadian Angus cows. *PLOS One*. 2020;15(8):e0237818. 32853245 10.1371/journal.pone.0237818 PMC7451565

[51] Zhou C, Li C, Cai W, et al. Genome-wide association study for milk protein composition traits in a Chinese Holstein population using a single-step approach. *Front Genet*. 2019;10:72. 30838020 10.3389/fgene.2019.00072 PMC6389681

[52] Lemos MVA, Chiaia HLJ, Berton MP, et al. Genome-wide association between single nucleotide polymorphisms with beef fatty acid profile in Nellore cattle using the single step procedure. *BMC Genomics*. 2016;17(1):213. 26960694 10.1186/s12864-016-2511-y PMC4784275

[53] Sweett H, Fonseca PAS, Suárez-Vega A, Livernois A, Miglior F, Cánovas A. Genome-wide association study to identify genomic regions and positional candidate genes associated with male fertility in beef cattle. *Sci Rep*. 2020;10(1):20102. 33208801 10.1038/s41598-020-75758-3 PMC7676258

[54] R Core Team. R: A language and environment for statistical computing. R Foundation for Statistical Computing; 2022. https://www.R-project.org/.

[55] Atashi H, Salavati M, De Koster J, Crowe MA, Opsomer G, Hostens M. Genome-wide association for metabolic clusters in early-lactation Holstein dairy cows. *J Dairy Sci*. 2020;103(7):6392–6406. 32331880 10.3168/jds.2019-17369

[56] Harrison PW, Amode MR, Austine-Orimoloye O, et al. Ensembl 2024. *Nucleic Acids Res*. 2024;52(D1):D891–D899. 37953337 10.1093/nar/gkad1049 PMC10767893

[57] Huang DW, Sherman BT, Lempicki RA. Systematic and integrative analysis of large gene lists using DAVID bioinformatics resources. *Nat Protoc*. 2009;4(1):44–57. 19131956 10.1038/nprot.2008.211

[58] Sherman BT, Hao M, Qiu J, et al. DAVID: a web server for functional enrichment analysis and functional annotation of gene lists (2021 update). *Nucleic Acids Res*. 2022;50(W1):W216–W221. 35325185 10.1093/nar/gkac194 PMC9252805

[59] Szklarczyk D, Kirsch R, Koutrouli M, et al. The STRING database in 2023: protein-protein association networks and functional enrichment analyses for any sequenced genome of interest. *Nucleic Acids Res*. 2023;51(D1):D638–D646. 36370105 10.1093/nar/gkac1000 PMC9825434

[60] Hu ZL, Park CA, Reecy JM. Bringing the animal QTLdb and CorrDB into the future: meeting new challenges and providing updated services. *Nucleic Acids Res*. 2022;50(D1):D956–D961. 34850103 10.1093/nar/gkab1116 PMC8728226

[61] Kheirabadi K, Razmkabir M. Genetic parameters for daily milk somatic cell score and relationships with yield traits of primiparous Holstein cattle in Iran. *J Anim Sci Technol*. 2016;58(1):38. 27800174 10.1186/s40781-016-0121-5 PMC5081878

[62] Sabedot MA, Romano GS, Pedrosa VB, Pinto LFB. Genetic parameters for milk traits, somatic cell, and total bacteria count scores in Brazilian Jersey herds. *R Bras Zootec*. 2018;47:e20160351.

[63] Atashi H, Hostens M. Genetic aspects of somatic cell count in Holstein dairy cows in Iran. *Animals*. 2021;11(6):1637. 34205847 10.3390/ani11061637 PMC8226697

[64] Kosciuczuk EM, Lisowski P, Jarczak J, et al. Transcriptome profiling of Staphylococci-infected cow mammary gland parenchyma. *BMC Vet Res*. 2017;13(1):1–12. 28587645 10.1186/s12917-017-1088-2 PMC5477815

[65] Soria-Valles C, Gutiérrez-Fernández A, Osorio FG, et al. MMP-25 metalloprotease regulates innate immune response through NF-κB signaling. *J Immunol*. 2016;197(1):296–302. 27259858 10.4049/jimmunol.1600094

[66] Scheau C, Badarau IA, Costache R, et al. The role of matrix metalloproteinases in the epithelial-mesenchymal transition of hepatocellular carcinoma. *Anal Cell Pathol*. 2019;2019:9423907. 10.1155/2019/9423907 PMC6899323 31886121

[67] Ilie DE, Mizeranschi AE, Mihali CV, et al. Genome-wide association studies for milk somatic cell score in Romanian dairy cattle. *Genes*. 2021;12(10):1495. 34680890 10.3390/genes12101495 PMC8535694

[68] Cheng Z, Buggiotti L, Salavati M, et al. Global transcriptomic profiles of circulating leucocytes in early lactation cows with clinical or subclinical mastitis. *Mol Biol Rep*. 2021;48(5):4611–4623. 34146201 10.1007/s11033-021-06494-8

[69] Ashja A, Zorc M, Dovc P. Genome-wide association study for milk somatic cell score in Holstein Friesian cows in Slovenia. *Animals*. 2024;14(18):2713. 39335302 10.3390/ani14182713 PMC11429251

[70] Depreester E, Meyer E, Demeyere K, Van Eetvelde M, Hostens M, Opsomer G. Flow cytometric assessment of myeloperoxidase in bovine blood neutrophils and monocytes. *J Dairy Sci*. 2017;100(9):7638–7647. 28690058 10.3168/jds.2016-12186

[71] Alhussien MN, Panda BSK, Dang AK. A comparative study on changes in total and differential milk cell counts, activity, and expression of milk phagocytes of healthy and mastitic indigenous Sahiwal cows. *Front Vet Sci*. 2021;8:670811. 34235202 10.3389/fvets.2021.670811 PMC8255372

[72] Ciliberti MG, Santillo A, Caroprese M, Albenzio M. Cytokine profile, differential somatic cell count, and oxidative status of Italian Mediterranean buffalo milk affected by the temperature–humidity index. *Front Vet Sci*. 2024;11:1449017. 39606644 10.3389/fvets.2024.1449017 PMC11599857

[73] Alhussien MN, Dang AK. Sensitive and rapid lateral-flow assay for early detection of subclinical mammary infection in dairy cows. *Sci Rep*. 2020;10(1):11161. 32636460 10.1038/s41598-020-68174-0 PMC7341798

[74] Bakhtiarizadeh MR, Mirzaei S, Norouzi M, Sheybani N, Vafaei Sadi MS. Identification of gene modules and hub genes involved in mastitis development using a systems biology approach. *Front Genet*. 2020;11:722. 32754201 10.3389/fgene.2020.00722 PMC7371005

[75] Sikka P, Singh KP, Singh I, et al. Whole blood transcriptome analysis of lactating Murrah buffaloes divergent to contrasting genetic merits for milk yield. *Front Anim Sci*. 2023;4:1–17.

[76] Fang L, Hou Y, An J, et al. Genome-wide transcriptional and post-transcriptional regulation of innate immune and defense responses of bovine mammary gland to *Staphylococcus aureus*. *Front Cell Infect Microbiol*. 2016;6:193. 28083515 10.3389/fcimb.2016.00193 PMC5183581

[77] Olsaker I, Boman GM, Argreth Downing A, Talbot R, Storset AK. The early phase transcriptome of bovine monocyte-derived macrophages infected with *Staphylococcus aureus in vitro*. *BMC Genomics*. 2013;14:891. 24341851 10.1186/1471-2164-14-891 PMC3878444

[78] Anczuków O, Rosenberg AZ, Akerman M, et al. The splicing factor SRSF1 regulates apoptosis and proliferation to promote mammary epithelial cell transformation. *Nat Struct Mol Biol*. 2012;19(2):220–228. 22245967 10.1038/nsmb.2207 PMC3272117

[79] Gonçalves V, Jordan P. Posttranscriptional regulation of splicing factor SRSF1 and its role in cancer cell biology. *Biomed Res Int*. 2015;2015:1–10. 10.1155/2015/287048 PMC4529898 26273603

[80] Lo Giudice A, Asmundo MG, Broggi G, et al. The clinical role of SRSF1 expression in cancer: a review of the current literature. *Appl Sci*. 2022;12(5):2268.

[81] Tang S, Liu D, Fang Y, et al. Low expression of HIF1AN accompanied by less immune infiltration is associated with poor prognosis in breast cancer. *Front Oncol*. 2023;13:1080910. 36816977 10.3389/fonc.2023.1080910 PMC9932925

[82] Pedrosa VB, Schenkel FS, Chen S-Y, et al. Genomewide association analyses of lactation persistency and milk production traits in Holstein cattle based on imputed whole-genome sequence data. *Genes*. 2021;12(11):1830. 34828436 10.3390/genes12111830 PMC8624223

[83] Atashi H, Chen Y, Wilmot H, et al. Single-step genome-wide association for selected milk fatty acids in dual-purpose Belgian blue cows. *J Dairy Sci*. 2023;106(9):6299–6315. 37479585 10.3168/jds.2022-22432

[84] Kim DJ, Yi YW, Seong YS. Beta-transducin repeats-containing proteins as an anticancer target. *Cancers*. 2023;15(17):4248. 37686524 10.3390/cancers15174248 PMC10487276

[85] Marete A, Lund MS, Boichard D, Ramayo-Caldas Y. A system-based analysis of the genetic determinism of udder conformation and health phenotypes across three French dairy cattle breeds. *PLOS One*. 2018;13(7):e0199931. 29965995 10.1371/journal.pone.0199931 PMC6028091

[86] Raven L-A, Cocks BG, Kemper KE, et al. Targeted imputation of sequence variants and gene expression profiling identifies twelve candidate genes associated with lactation volume, composition and calving interval in dairy cattle. *Mamm Genome*. 2016;27(1–2):81–97. 26613780 10.1007/s00335-015-9613-8

[87] Maddahi N, Sadeghi M, Sarghale AJ, Saatchi M, Davar Siar MK, Kholghi M. Identification of candidate gene networks affecting the number of somatic cells count and milk production in Iranian Holstein cows using genome-wide association study. *Sci Rep*. 2025;15(1):32168. 1 40890164 10.1038/s41598-025-09103-x PMC12402163

