# Supplementary material for: Single-step genome-wide association study of milk somatic cell scores across multi-cattle breeds in Ethiopia
Source: Anim Biotechnol. 2025 Nov 18;36(1):2586262. doi: 10.1080/10495398.2025.2586262 (PMC12698049; doi:10.1080/10495398.2025.2586262)
Supplement: Supplementary_Figure_S1.docx [file LABT_A_2586262_SM4800.docx]

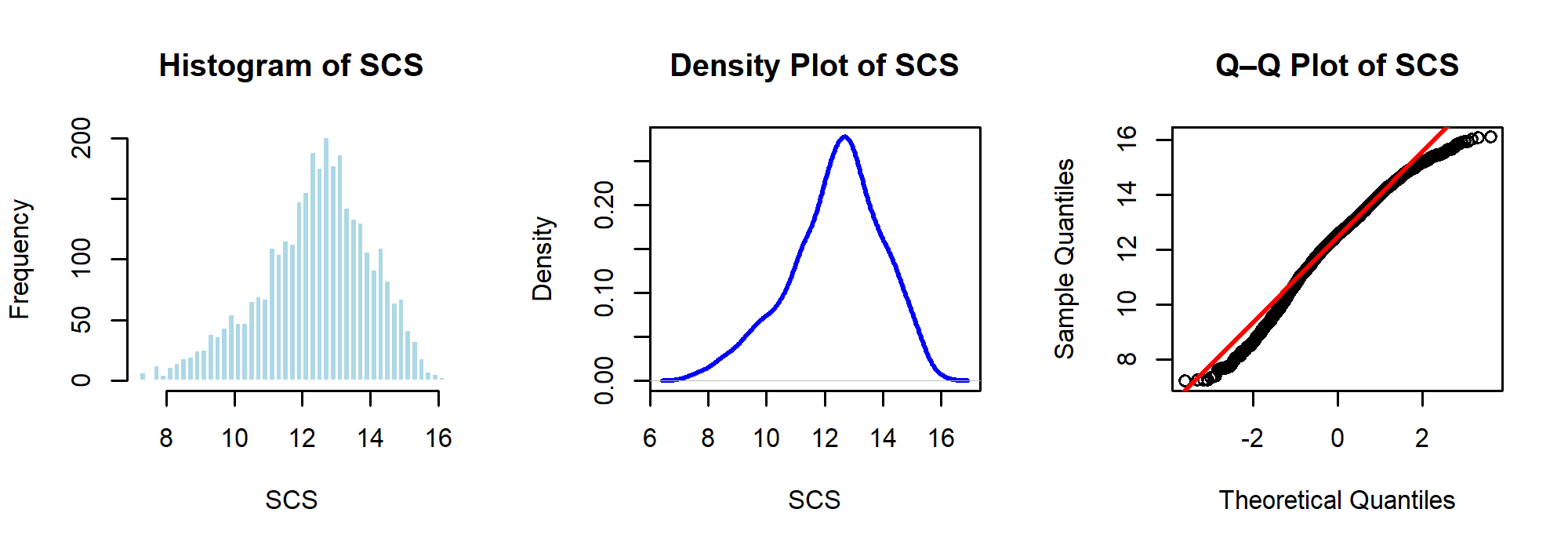


**Supplementary Figure S1.** Assessment of normality for transformed somatic cell score (SCS). The histogram, density plot, and Q–Q plot.
