# Supplementary material for: Single-step genome-wide association study of milk somatic cell scores across multi-cattle breeds in Ethiopia
Source: Anim Biotechnol. 2025 Nov 18;36(1):2586262. doi: 10.1080/10495398.2025.2586262 (PMC12698049; doi:10.1080/10495398.2025.2586262)
Supplement: Supplementary_Figures_S2_S6.docx [file LABT_A_2586262_SM4799.docx]

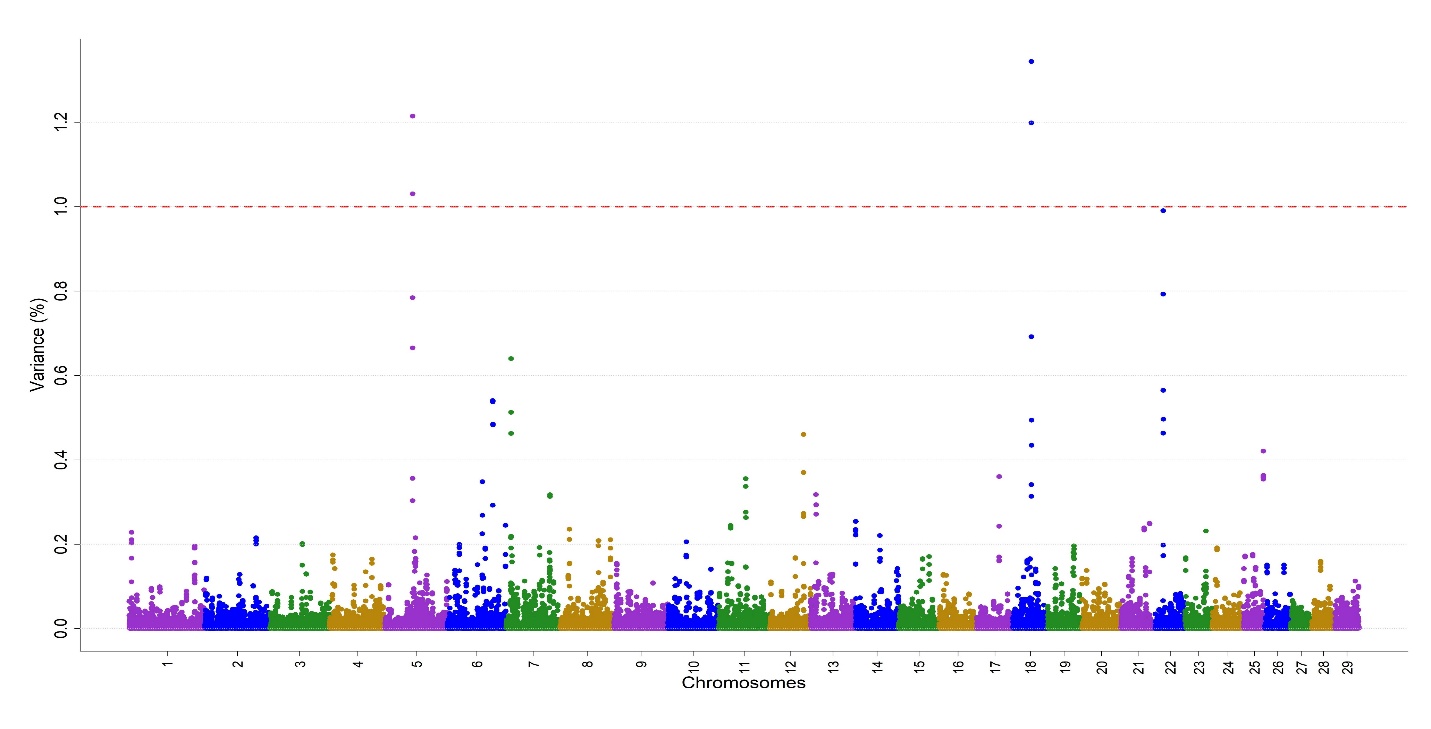


**Supplementary Figure S2.** Manhattan plots of the proportion of additive genetic variance (%) explained by moving windows of 5 adjacent SNPs for milk SCS in Ethiopian dairy cattle. Each dot denotes a window. The red broken line represents 1% of genetic variance.


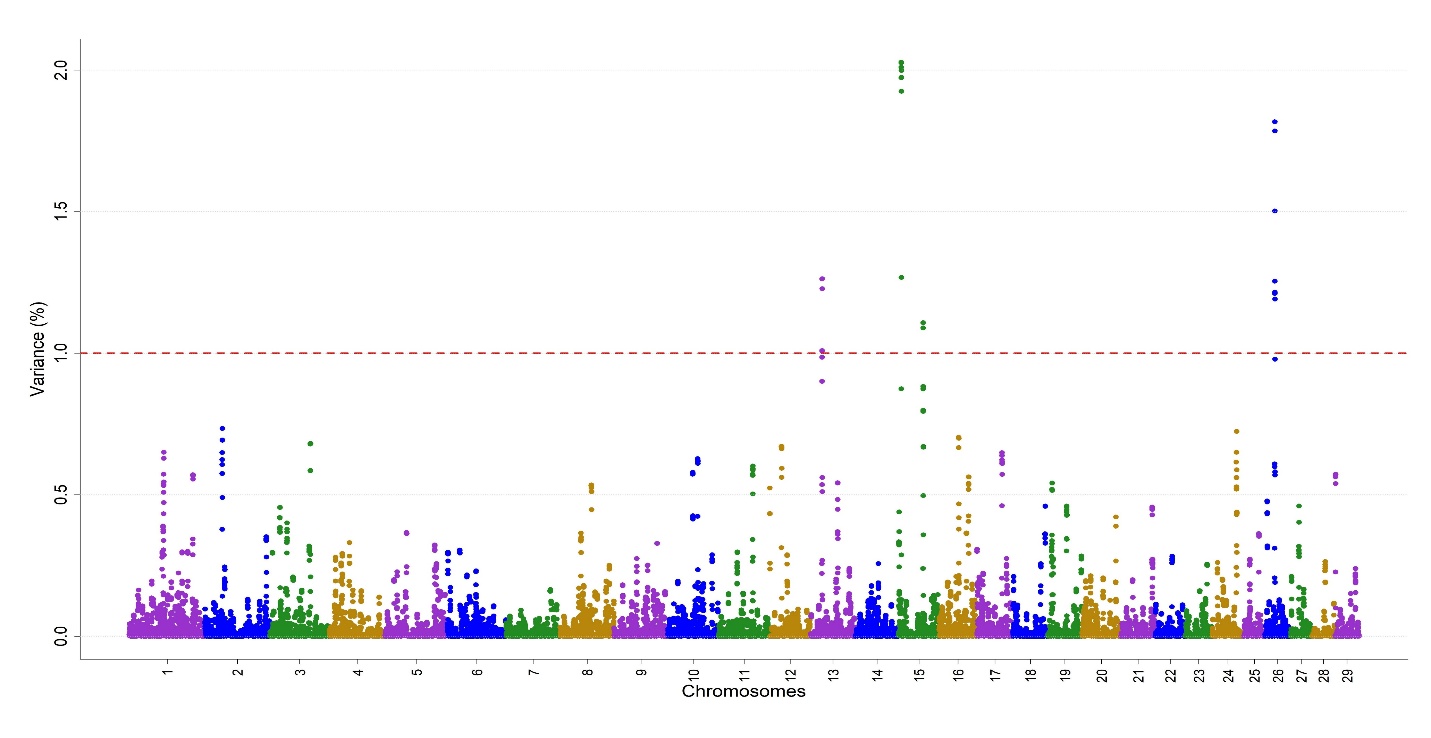


**Supplementary Figure S3.** Manhattan plots of the proportion of additive genetic variance (%) explained by moving windows of 10 adjacent SNPs for milk SCS in Ethiopian dairy cattle. Each dot denotes a window. The red broken line represents 1% of genetic variance.


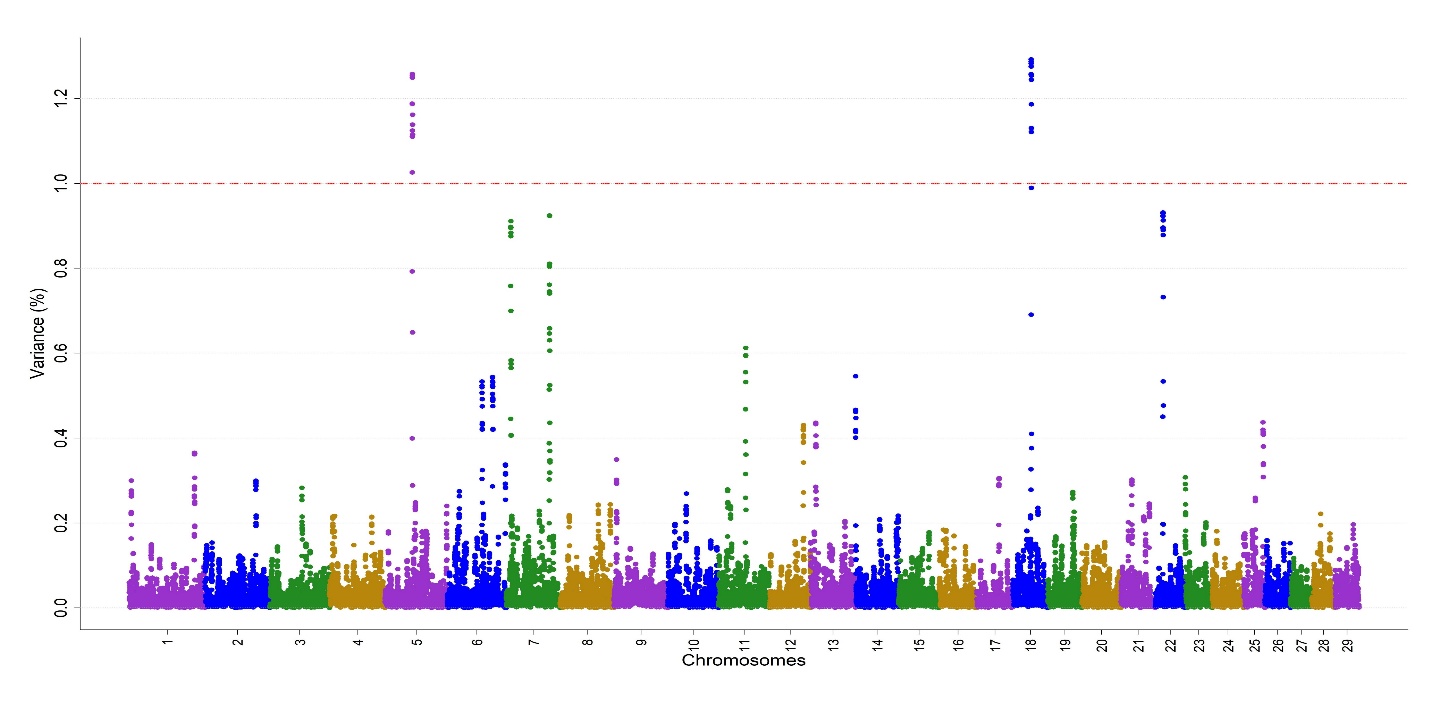
 **Supplementary Figure S4.** Manhattan plots of the proportion of additive genetic variance (%) explained by moving windows of 15 adjacent SNPs for milk SCS in Ethiopian dairy cattle. Each dot denotes a window. The red broken line represents 1% of genetic variance.


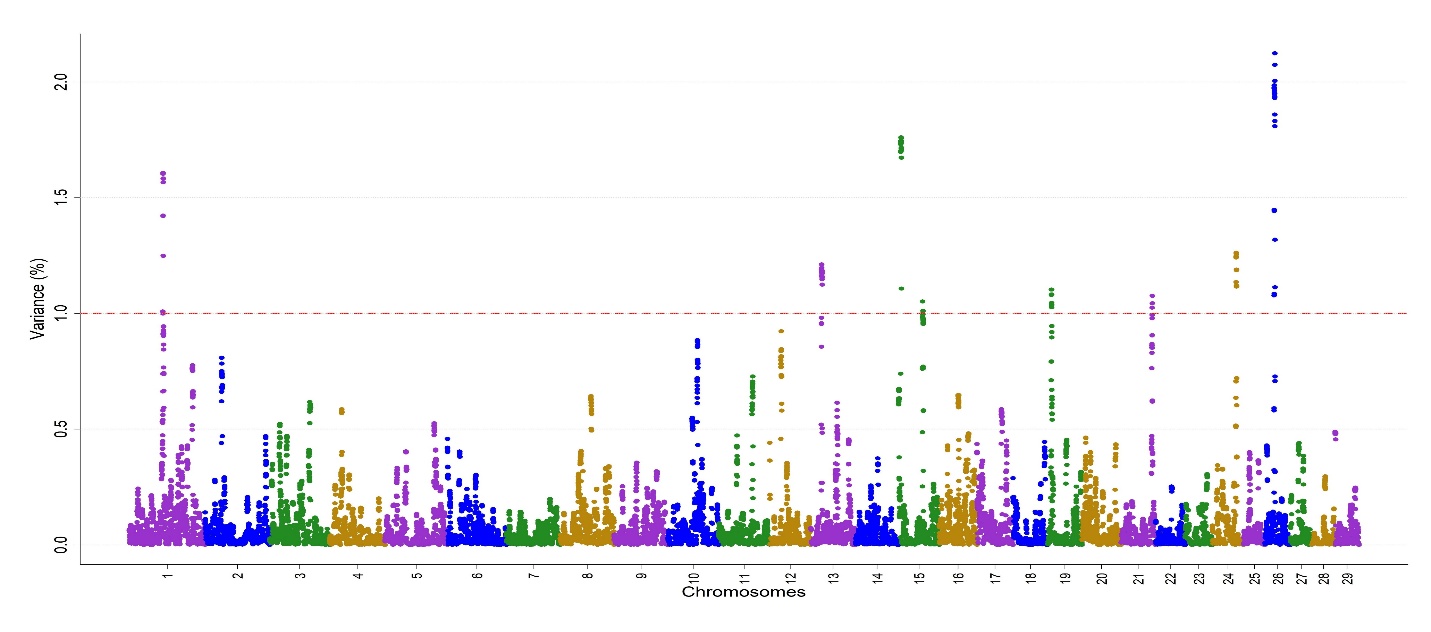
 **Supplementary Figure S5.** Manhattan plots of the proportion of additive genetic variance (%) explained by moving windows of 30 adjacent SNPs for milk SCS in Ethiopian dairy cattle. Each dot denotes a window. The red broken line represents 1% of genetic variance.


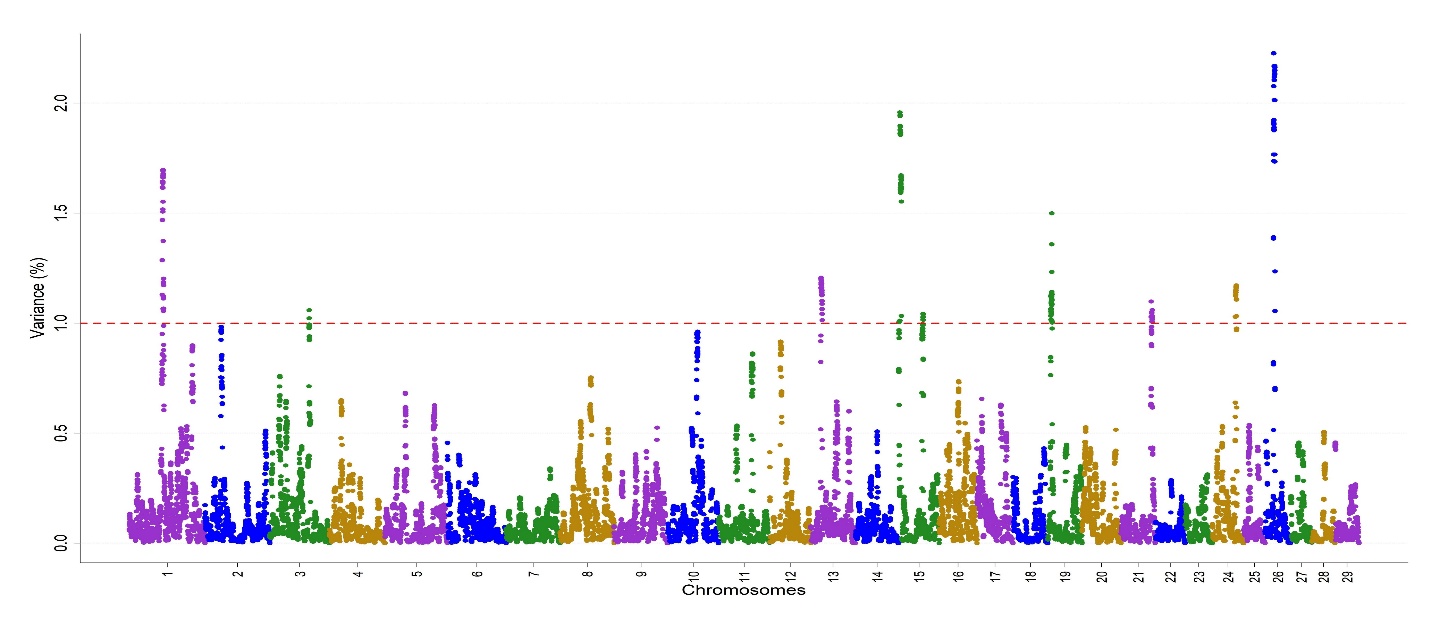
 **Supplementary Figure S6.** Manhattan plots of the proportion of additive genetic variance (%) explained by moving windows of 50 adjacent SNPs for milk SCS in Ethiopian dairy cattle. Each dot denotes a window. The red broken line represents 1% of genetic variance.
