# Supplementary material for: Single-step genome-wide association study of milk somatic cell scores across multi-cattle breeds in Ethiopia
Source: Anim Biotechnol. 2025 Nov 18;36(1):2586262. doi: 10.1080/10495398.2025.2586262 (PMC12698049; doi:10.1080/10495398.2025.2586262)
Supplement: Supplementary _Table_1_2.docx [file LABT_A_2586262_SM4798.docx]

**Supplementary Table 1.** Summary of annotated gens within SCS-associated genomic regions that are poorly characterized in cattle

| **Chr^1^** | **SNP window^2^** | | **Var (%)^3^** | **Annotated gene^4^** |
| --- | --- | --- | --- | --- |
|  | Start,bp | Stop,bp |  |  |
| 15 | 5674820 | 7033930 | 1.20 | LOC112441598, LOC112441463, LOC100848277, LOC100336562, LOC132342298, LOC107133144, LOC104974165, LOC100848148, LOC783022, LOC112441715, |
| 19 | 8157946 | 9281946 | 1.10 | LOC101904749, LOC112442596, LOC132342994, LOC132342995, LOC132342996, LOC514828, OR4D2B, OR4D1B, OR4D2, OR4D1, OR4D2G, OR4D2E, OR4D2D, OR4D33P, OR4D2F, LOC788751, LOC112442599, LOC112442764, LOC112442768 |
| 26 | 21409429 | 23307206 | 2.55 | LOC104975965, LOC132343947, LOC112444556, LOC132343948, LOC132343949, LOC100847491, LOC132343992, LOC101908075, LOC132343929, LOC112444554, LOC112444524, LOC101902227, LOC785229, LOC132343951, LOC132343952, C26H10orf95, LOC132343954, LOC132343953 |
| *^1^Chr = chromosome.*  *^2^Starting and ending coordinates of the window base pairs, with 20 consecutive SNPs.*  *^3^Var = % genetic variance explained by the SNPs within the window.*  *^4^Ensembl symbol of annotated genes using the Bos taurus ARS-UCD2.0 assembly (http: / / www. ensembl .org/ biomart/ martview) and NCBI Genome Data Viewer for domestic cattle (Bos taurus) - (https://www.ncbi.nlm.nih.gov/gdv?org=bos-taurus&group=bovinae)* | | | | |

**Supplementary Table 2.** QTL identified in the defined genomic region linked with milk SCS in cattle

| **Chr** | **DataType** | **QTLID** | **Trait Name** | **base pair** | **Breed** |
| --- | --- | --- | --- | --- | --- |
| 15 | Association | QTL:245405 | Milk fat yield | 5700833-5700837 | Holstein |
| 15 | Association | QTL:238553 | Somatic cell score | 5686806-5686810 | Baltana Romaneasca |
| 15 | QTL | QTL:3180 | Veterinary treatments | 6395340-17110034 |  |
| 15 | QTL | QTL:3571 | Non-return rate | 17110034-0 |  |
| 15 | QTL | QTL:5290 | Feed conversion ratio | 4959430-27974287 |  |
| 15 | QTL | QTL:19626 | Tridecylic acid content | 20-59060134 | Angus |
| 15 | QTL | QTL:166717 | Cystic ovaries | 5134162-6310157 | Holstein |
| 15 | Association | QTL:68764 | Body weight gain | 5724089-5724093 | Charolais, Gelbvieh, Hereford, Limousin, Pinzgauer, Red angus, Red poll, Simmental, Angus |
| 15 | QTL | QTL:10981 | Body weight | 0-6395340 | Angus |
| 15 | QTL | QTL:10980 | Marbling score | 0-6395340 | Angus |
| 15 | QTL | QTL:10979 | Body height | 0-6395340 | Angus |
| 15 | Association | QTL:179218 | Blood calcium level | 6300940-6300944 | Holstein |
| 15 | Association | QTL:57624 | Bovine respiratory disease susceptibility | 6225472-6225512 |  |
| 15 | Association | QTL:96417 | Bovine tuberculosis susceptibility | 6552662-6552666 | Holstein |
| 15 | QTL | QTL:1319 | Kidney, pelvic, and heart fat percentage | 6395340-18019005 |  |
| 19 | Association | QTL:242544 | Milk yield | 8593909-8593913 | Holstein |
| 19 | Association | QTL:242545 | Milk yield | 8627948-8627952 | Holstein |
| 19 | Association | QTL:242546 | Milk yield | 8625412-8625416 | Holstein |
| 19 | Association | QTL:221178 | Bovine coronavirus susceptibility | 8360866-8360870 | Charolais, Hereford, Holstein, Red angus, Angus |
| 19 | Association | QTL:221199 | Bovine coronavirus susceptibility | 8360866-8360870 | Holstein |
| 19 | Association | QTL:221311 | Bovine respiratory disease susceptibility | 8360866-8360870 | Charolais, Hereford, Holstein, Red angus, Angus |
| 19 | Association | QTL:221395 | Bovine respiratory disease susceptibility | 8360866-8360870 | Holstein |
| 19 | Association | QTL:253639 | Milk protein percentage | 8626385-8626389 | Holstein |
| 19 | Association | QTL:221198 | Bovine coronavirus susceptibility | 8325970-8325974 | Holstein |
| 19 | Association | QTL:221394 | Bovine respiratory disease susceptibility | 8325970-8325974 | Holstein |
| 19 | Association | QTL:316121 | Milk yield | 9212876-9212880 | Holstein |
| 19 | Association | QTL:316137 | Milk yield | 9212875-9212879 | Holstein |
| 19 | Association | QTL:242551 | Milk yield | 9159365-9159369 | Holstein |
| 19 | Association | QTL:232723 | Marbling score | 9279003-9279007 | Angus |
| 19 | Association | QTL:232724 | Marbling score | 8683660-8683664 | Angus |
| 19 | Association | QTL:242552 | Milk yield | 8275176-8275180 | Holstein |
| 19 | Association | QTL:242556 | Milk yield | 8373635-8373639 | Holstein |
| 19 | Association | QTL:221197 | Bovine coronavirus susceptibility | 8294505-8294509 | Holstein |
| 19 | Association | QTL:232730 | Marbling score | 8976140-8976144 | Angus |
| 19 | Association | QTL:226766 | Shear force | 8193851-8193855 | Angus |
| 19 | Association | QTL:242558 | Milk yield | 8546970-8546974 | Holstein |
| 19 | Association | QTL:286849 | Body weight | 8344352-8344356 | Holstein, Simmental |
| 19 | Association | QTL:174686 | Milk yield | 8596152-8596156 | Holstein |
| 19 | QTL | QTL:2637 | Average daily gain | 3098379-9285876 |  |
| 19 | QTL | QTL:3547 | Subcutaneous fat thickness | 3098379-9285876 |  |
| 19 | QTL | QTL:3577 | Structural soundness | 0-9285876 |  |
| 19 | QTL | QTL:106743 | Udder swelling score | 7937747-8780884 | Limousin |
| 19 | Association | QTL:163909 | Milk yield | 8418325-8418329 | Jersey |
| 19 | Association | QTL:102461 | Milk yield | 8300470-8300474 | Holstein |
| 19 | Association | QTL:212866 | Inseminations per conception | 8527488-8527492 | Holstein |
| 19 | Association | QTL:102462 | Milk yield | 8312417-8312421 | Holstein |
| 19 | Association | QTL:215498 | Milk protein percentage | 8412277-8412281 | Braunvieh, Holstein, Jersey, Normande, Norwegian red, Fleckvieh, MontbÃ©liarde, Australian Red |
| 19 | Association | QTL:102492 | Milk yield | 8423545-8423549 | Holstein |
| 19 | Association | QTL:155599 | Milk tridecylic acid content | 8670753-8670757 | Holstein |
| 19 | Association | QTL:181363 | Conception rate | 8798122-8798126 | Holstein |
| 19 | Association | QTL:181533 | Inseminations per conception | 8798122-8798126 | Holstein |
| 19 | Association | QTL:146016 | Stillbirth | 8575561-8575565 | Brown swiss |
| 19 | Association | QTL:145989 | Stillbirth | 8605644-8605648 | Brown swiss |
| 19 | Association | QTL:145990 | Stillbirth | 8606282-8606286 | Brown swiss |
| 19 | Association | QTL:145988 | Stillbirth | 8605185-8605189 | Brown swiss |
| 19 | Association | QTL:146186 | Stillbirth | 8606456-8606460 | Brown swiss |
| 19 | Association | QTL:146193 | Stillbirth | 8606965-8606969 | Brown swiss |
| 19 | Association | QTL:146182 | Stillbirth | 8606434-8606438 | Brown swiss |
| 19 | Association | QTL:146195 | Stillbirth | 8607024-8607028 | Brown swiss |
| 19 | Association | QTL:146190 | Stillbirth | 8606533-8606537 | Brown swiss |
| 19 | Association | QTL:106030 | Milk protein percentage | 8625412-8625416 | Holstein |
| 19 | QTL | QTL:11074 | Body weight | 3098379-9285876 | Angus |
| 19 | QTL | QTL:11073 | Scrotal circumference | 3098379-9285876 | Angus |
| 19 | Association | QTL:146150 | Stillbirth | 8588457-8588461 | Brown swiss |
| 19 | Association | QTL:145985 | Stillbirth | 8606000-8606004 | Brown swiss |
| 19 | Association | QTL:146103 | Stillbirth | 8730582-8730586 | Brown swiss |
| 19 | Association | QTL:65293 | Body weight | 8775185-8775189 | Brahman |
| 19 | Association | QTL:65541 | Maturity rate | 8775185-8775189 | Brahman |
| 19 | Association | QTL:146135 | Stillbirth | 8699133-8699137 | Brown swiss |
| 19 | Association | QTL:146140 | Stillbirth | 8699593-8699597 | Brown swiss |
| 19 | Association | QTL:146120 | Stillbirth | 8818855-8818859 | Brown swiss |
| 19 | Association | QTL:146119 | Stillbirth | 8819189-8819193 | Brown swiss |
| 19 | Association | QTL:146113 | Stillbirth | 8826733-8826737 | Brown swiss |
| 19 | Association | QTL:146115 | Stillbirth | 8826089-8826093 | Brown swiss |
| 19 | Association | QTL:146116 | Stillbirth | 8822286-8822290 | Brown swiss |
| 19 | Association | QTL:146110 | Stillbirth | 8830189-8830193 | Brown swiss |
| 19 | Association | QTL:146118 | Stillbirth | 8828381-8828385 | Brown swiss |
| 19 | Association | QTL:146114 | Stillbirth | 8827805-8827809 | Brown swiss |
| 19 | Association | QTL:146111 | Stillbirth | 8830889-8830893 | Brown swiss |
| 19 | Association | QTL:146112 | Stillbirth | 8832986-8832990 | Brown swiss |
| 19 | Association | QTL:146117 | Stillbirth | 8836370-8836374 | Brown swiss |
| 19 | Association | QTL:146122 | Stillbirth | 8836925-8836929 | Brown swiss |
| 19 | Association | QTL:146109 | Stillbirth | 8839866-8839870 | Brown swiss |
| 19 | Association | QTL:146098 | Stillbirth | 8846360-8846364 | Brown swiss |
| 19 | Association | QTL:145997 | Stillbirth | 8863578-8863582 | Brown swiss |
| 19 | Association | QTL:146105 | Stillbirth | 8918112-8918116 | Brown swiss |
| 19 | Association | QTL:146099 | Stillbirth | 8879688-8879692 | Brown swiss |
| 19 | Association | QTL:146097 | Stillbirth | 8893993-8893997 | Brown swiss |
| 19 | Association | QTL:146096 | Stillbirth | 8894317-8894321 | Brown swiss |
| 19 | Association | QTL:146106 | Stillbirth | 9165745-9165749 | Brown swiss |
| 19 | Association | QTL:146100 | Stillbirth | 9168494-9168498 | Brown swiss |
| 19 | Association | QTL:105638 | Milk protein percentage | 9182176-9182180 | Holstein |
| 19 | Association | QTL:105546 | Milk protein percentage | 9183068-9183072 | Holstein |
| 19 | Association | QTL:105787 | Milk protein percentage | 9185070-9185074 | Holstein |
| 19 | Association | QTL:106112 | Milk protein percentage | 9195471-9195475 | Holstein |
| 19 | Association | QTL:106040 | Milk protein percentage | 9215704-9215708 | Holstein |
| 19 | Association | QTL:106041 | Milk protein percentage | 9217829-9217833 | Holstein |
| 19 | Association | QTL:106042 | Milk protein percentage | 9229624-9229628 | Holstein |
| 19 | Association | QTL:146048 | Stillbirth | 9169253-9169257 | Brown swiss |
| 19 | QTL | QTL:1339 | Yield grade | 0-21420040 |  |
| 19 | Association | QTL:12011 | Myristic acid content | 8631697-8770638 |  |
| 19 | Association | QTL:12012 | Myristoleic acid content | 8631697-8770638 |  |
| 19 | Association | QTL:12013 | Palmitoleic acid content | 8631697-8770638 |  |
| 19 | Association | QTL:12014 | cis-10-Heptadecenoic acid content | 8631697-8770638 |  |
| 19 | Association | QTL:12015 | trans-Vaccenic acid content | 8631697-8770638 |  |
| 19 | Association | QTL:12016 | cis-Vaccenic acid content | 8631697-8770638 |  |
| 19 | QTL | QTL:10443 | Milk fat yield | 8162772-34966768 | Holstein |
| 19 | QTL | QTL:10446 | Somatic cell score | 2373572-61307628 | Holstein |
| 19 | QTL | QTL:1338 | Retail product yield | 0-8683800 |  |
| 19 | QTL | QTL:11072 | Body weight | 0-9285876 | Angus |
| 19 | QTL | QTL:11071 | Body weight | 0-9285876 | Angus |
| 19 | QTL | QTL:10444 | Milk fat percentage | 1389408-52681720 | Holstein |
| 19 | QTL | QTL:10445 | Milk protein percentage | 2373572-59397192 | Holstein |
| 19 | QTL | QTL:10021 | Oleic acid content | 9262720-44576840 | Jersey, Limousin |
| 19 | QTL | QTL:10027 | Milk stearic acid content | 6368120-53839560 | Holstein |
| 26 | Association | QTL:254850 | Milk protein percentage | 22599205-22599209 | Holstein |
| 26 | Association | QTL:254852 | Milk protein percentage | 22990255-22990259 | Holstein |
| 26 | Association | QTL:254853 | Milk protein percentage | 22524366-22524370 | Holstein |
| 26 | Association | QTL:245540 | Milk fat yield | 21722491-21722495 | Holstein |
| 26 | Association | QTL:254854 | Milk protein percentage | 23002944-23002948 | Holstein |
| 26 | Association | QTL:245541 | Milk fat yield | 22010297-22010301 | Holstein |
| 26 | Association | QTL:254855 | Milk protein percentage | 23052377-23052381 | Holstein |
| 26 | Association | QTL:245549 | Milk fat yield | 23210092-23210096 | Holstein |
| 26 | Association | QTL:254856 | Milk protein percentage | 22496545-22496549 | Holstein |
| 26 | Association | QTL:245554 | Milk fat yield | 21722160-21722164 | Holstein |
| 26 | Association | QTL:245563 | Milk fat yield | 21453883-21453887 | Holstein |
| 26 | Association | QTL:254858 | Milk protein percentage | 22663390-22663394 | Holstein |
| 26 | Association | QTL:254859 | Milk protein percentage | 23016228-23016232 | Holstein |
| 26 | Association | QTL:245567 | Milk fat yield | 21419518-21419522 | Holstein |
| 26 | Association | QTL:245568 | Milk fat yield | 22221436-22221440 | Holstein |
| 26 | Association | QTL:254860 | Milk protein percentage | 22791748-22791752 | Holstein |
| 26 | Association | QTL:254861 | Milk protein percentage | 22771448-22771452 | Holstein |
| 26 | Association | QTL:245578 | Milk fat yield | 21445142-21445146 | Holstein |
| 26 | Association | QTL:249357 | Milk fat percentage | 21617319-21617323 | Holstein |
| 26 | Association | QTL:254865 | Milk protein percentage | 22468171-22468175 | Holstein |
| 26 | Association | QTL:254866 | Milk protein percentage | 22967472-22967476 | Holstein |
| 26 | Association | QTL:254868 | Milk protein percentage | 22469086-22469090 | Holstein |
| 26 | Association | QTL:254870 | Milk protein percentage | 22468894-22468898 | Holstein |
| 26 | Association | QTL:245618 | Milk fat yield | 22115359-22115363 | Holstein |
| 26 | Association | QTL:245619 | Milk fat yield | 22137362-22137366 | Holstein |
| 26 | Association | QTL:245620 | Milk fat yield | 22133377-22133381 | Holstein |
| 26 | Association | QTL:234908 | Connective tissue amount | 22153601-22153605 | Angus |
| 26 | Association | QTL:245622 | Milk fat yield | 22176406-22176410 | Holstein |
| 26 | Association | QTL:245623 | Milk fat yield | 22209701-22209705 | Holstein |
| 26 | Association | QTL:245624 | Milk fat yield | 22242033-22242037 | Holstein |
| 26 | Association | QTL:245625 | Milk fat yield | 22247162-22247166 | Holstein |
| 26 | Association | QTL:254871 | Milk protein percentage | 23129691-23129695 | Holstein |
| 26 | Association | QTL:254872 | Milk protein percentage | 23117244-23117248 | Holstein |
| 26 | Association | QTL:254873 | Milk protein percentage | 23114655-23114659 | Holstein |
| 26 | Association | QTL:254874 | Milk protein percentage | 23062180-23062184 | Holstein |
| 26 | Association | QTL:254875 | Milk protein percentage | 23078284-23078288 | Holstein |
| 26 | Association | QTL:254876 | Milk protein percentage | 23088322-23088326 | Holstein |
| 26 | Association | QTL:254877 | Milk protein percentage | 22962092-22962096 | Holstein |
| 26 | Association | QTL:245626 | Milk fat yield | 21437338-21437342 | Holstein |
| 26 | Association | QTL:245628 | Milk fat yield | 21610246-21610250 | Holstein |
| 26 | Association | QTL:245629 | Milk fat yield | 21539985-21539989 | Holstein |
| 26 | Association | QTL:254879 | Milk protein percentage | 22772591-22772595 | Holstein |
| 26 | Association | QTL:254880 | Milk protein percentage | 22812858-22812862 | Holstein |
| 26 | Association | QTL:254881 | Milk protein percentage | 22847959-22847963 | Holstein |
| 26 | Association | QTL:245631 | Milk fat yield | 21505508-21505512 | Holstein |
| 26 | Association | QTL:245639 | Milk fat yield | 21719871-21719875 | Holstein |
| 26 | Association | QTL:245641 | Milk fat yield | 21748665-21748669 | Holstein |
| 26 | Association | QTL:254887 | Milk protein percentage | 22677163-22677167 | Holstein |
| 26 | Association | QTL:245664 | Milk fat yield | 23232003-23232007 | Holstein |
| 26 | Association | QTL:245665 | Milk fat yield | 23227628-23227632 | Holstein |
| 26 | Association | QTL:245666 | Milk fat yield | 23186206-23186210 | Holstein |
| 26 | Association | QTL:254889 | Milk protein percentage | 22913406-22913410 | Holstein |
| 26 | Association | QTL:254893 | Milk protein percentage | 22894184-22894188 | Holstein |
| 26 | Association | QTL:245761 | Milk fat yield | 21445477-21445481 | Holstein |
| 26 | Association | QTL:255094 | Milk protein percentage | 22859043-22859047 | Holstein |
| 26 | QTL | QTL:239769 | Milk fat yield | 22025162-22025166 | Holstein, Jersey |
| 26 | QTL | QTL:239774 | Milk fat percentage | 22133890-22133894 | Holstein, Jersey |
| 26 | Association | QTL:296875 | Residual feed intake | 21421886-21421890 | Holstein |
| 26 | Association | QTL:296876 | Residual feed intake | 21422280-21422284 | Holstein |
| 26 | Association | QTL:296877 | Residual feed intake | 21423240-21423244 | Holstein |
| 26 | QTL | QTL:239763 | Milk yield | 22033047-22033051 | Holstein, Jersey |
| 26 | Association | QTL:125830 | Subcutaneous fat thickness | 22512567-22512571 | Charolais, Angus |
| 26 | Association | QTL:125831 | Lean meat yield | 22512567-22512571 | Charolais, Angus |
| 26 | Association | QTL:125832 | Longissimus muscle area | 22512567-22512571 | Charolais, Angus |
| 26 | Association | QTL:174334 | Milk fat yield | 23017986-23017990 | Holstein |
| 26 | Association | QTL:174327 | Milk fat yield | 21455314-21455318 | Holstein |
| 26 | Association | QTL:100441 | Milk fat yield | 22144758-22144798 | Ayrshire, Danish red, Swedish Red-and-White |
| 26 | Association | QTL:99601 | Milk fat yield | 23205860-23205864 | Ayrshire, Danish red, Swedish Red-and-White |
| 26 | Association | QTL:100409 | Milk fat yield | 22274880-22274884 | Ayrshire, Danish red, Swedish Red-and-White |
| 26 | Association | QTL:100141 | Milk fat yield | 22863642-22863646 | Ayrshire, Danish red, Swedish Red-and-White |
| 26 | Association | QTL:100307 | Milk fat yield | 22279598-22279602 | Ayrshire, Danish red, Swedish Red-and-White |
| 26 | Association | QTL:100135 | Milk fat yield | 22871297-22871301 | Ayrshire, Danish red, Swedish Red-and-White |
| 26 | Association | QTL:100183 | Milk fat yield | 22855492-22855496 | Ayrshire, Danish red, Swedish Red-and-White |
| 26 | Association | QTL:100133 | Milk fat yield | 22871709-22871713 | Ayrshire, Danish red, Swedish Red-and-White |
| 26 | Association | QTL:100109 | Milk fat yield | 22302520-22302524 | Ayrshire, Danish red, Swedish Red-and-White |
| 26 | Association | QTL:100159 | Milk fat yield | 22293750-22293754 | Ayrshire, Danish red, Swedish Red-and-White |
| 26 | Association | QTL:100145 | Milk fat yield | 22861100-22861104 | Ayrshire, Danish red, Swedish Red-and-White |
| 26 | Association | QTL:174332 | Milk fat yield | 22107109-22107113 | Holstein |
| 26 | Association | QTL:174333 | Milk fat yield | 22144979-22144983 | Holstein |
| 26 | Association | QTL:174330 | Milk fat yield | 22187704-22187708 | Holstein |
| 26 | Association | QTL:174331 | Milk fat yield | 22247162-22247166 | Holstein |
| 26 | Association | QTL:174329 | Milk fat yield | 21539985-21539989 | Holstein |
| 26 | Association | QTL:174328 | Milk fat yield | 21494661-21494665 | Holstein |
| 26 | Association | QTL:174335 | Milk fat yield | 23227628-23227632 | Holstein |
| 26 | Association | QTL:112594 | Milk kappa-casein percentage | 21637531-21637535 | Holstein |
| 26 | Association | QTL:119319 | Milk unglycosylated kappa-casein percentage | 21637531-21637535 | Holstein |
| 26 | Association | QTL:100090 | Milk fat yield | 22828698-22828702 | Ayrshire, Danish red, Swedish Red-and-White |
| 26 | Association | QTL:100049 | Milk fat yield | 22934019-22934023 | Ayrshire, Danish red, Swedish Red-and-White |
| 26 | Association | QTL:100447 | Milk fat yield | 22299232-22299236 | Ayrshire, Danish red, Swedish Red-and-White |
| 26 | Association | QTL:99718 | Milk fat yield | 23123539-23123543 | Ayrshire, Danish red, Swedish Red-and-White |
| 26 | Association | QTL:100178 | Milk fat yield | 22493971-22493975 | Ayrshire, Danish red, Swedish Red-and-White |
| 26 | Association | QTL:100096 | Milk fat yield | 22819837-22819841 | Ayrshire, Danish red, Swedish Red-and-White |
| 26 | Association | QTL:100069 | Milk fat yield | 22870226-22870230 | Ayrshire, Danish red, Swedish Red-and-White |
| 26 | Association | QTL:100078 | Milk fat yield | 22812364-22812368 | Ayrshire, Danish red, Swedish Red-and-White |
| 26 | Association | QTL:100007 | Milk fat yield | 23065869-23065873 | Ayrshire, Danish red, Swedish Red-and-White |
| 26 | Association | QTL:100037 | Milk fat yield | 23001055-23001059 | Ayrshire, Danish red, Swedish Red-and-White |
| 26 | Association | QTL:100046 | Milk fat yield | 22930808-22930812 | Ayrshire, Danish red, Swedish Red-and-White |
| 26 | Association | QTL:100022 | Milk fat yield | 22956787-22956791 | Ayrshire, Danish red, Swedish Red-and-White |
| 26 | Association | QTL:100018 | Milk fat yield | 22981498-22981502 | Ayrshire, Danish red, Swedish Red-and-White |
| 26 | Association | QTL:100055 | Milk fat yield | 22917242-22917246 | Ayrshire, Danish red, Swedish Red-and-White |
| 26 | Association | QTL:100016 | Milk fat yield | 22967258-22967262 | Ayrshire, Danish red, Swedish Red-and-White |
| 26 | Association | QTL:100061 | Milk fat yield | 22915633-22915637 | Ayrshire, Danish red, Swedish Red-and-White |
| 26 | Association | QTL:100057 | Milk fat yield | 22915467-22915471 | Ayrshire, Danish red, Swedish Red-and-White |
| 26 | Association | QTL:100387 | Milk fat yield | 23096912-23096916 | Ayrshire, Danish red, Swedish Red-and-White |
| 26 | Association | QTL:100040 | Milk fat yield | 22947399-22947403 | Ayrshire, Danish red, Swedish Red-and-White |
| 26 | Association | QTL:100086 | Milk fat yield | 22813441-22813445 | Ayrshire, Danish red, Swedish Red-and-White |
| 26 | Association | QTL:99888 | Milk fat yield | 22955096-22955100 | Ayrshire, Danish red, Swedish Red-and-White |
| 26 | Association | QTL:100066 | Milk fat yield | 22871187-22871191 | Ayrshire, Danish red, Swedish Red-and-White |
| 26 | Association | QTL:100103 | Milk fat yield | 22822537-22822541 | Ayrshire, Danish red, Swedish Red-and-White |
| 26 | Association | QTL:100035 | Milk fat yield | 23007055-23007059 | Ayrshire, Danish red, Swedish Red-and-White |
| 26 | Association | QTL:100062 | Milk fat yield | 22913720-22913724 | Ayrshire, Danish red, Swedish Red-and-White |
| 26 | Association | QTL:100024 | Milk fat yield | 22965558-22965562 | Ayrshire, Danish red, Swedish Red-and-White |
| 26 | Association | QTL:100010 | Milk fat yield | 22882039-22882043 | Ayrshire, Danish red, Swedish Red-and-White |
| 26 | Association | QTL:100045 | Milk fat yield | 22936402-22936406 | Ayrshire, Danish red, Swedish Red-and-White |
| 26 | Association | QTL:100184 | Milk fat yield | 22525063-22525067 | Ayrshire, Danish red, Swedish Red-and-White |
| 26 | Association | QTL:100267 | Milk fat yield | 23062607-23062611 | Ayrshire, Danish red, Swedish Red-and-White |
| 26 | Association | QTL:100041 | Milk fat yield | 23024273-23024277 | Ayrshire, Danish red, Swedish Red-and-White |
| 26 | Association | QTL:100019 | Milk fat yield | 22983442-22983446 | Ayrshire, Danish red, Swedish Red-and-White |
| 26 | Association | QTL:100239 | Milk fat yield | 23183927-23183931 | Ayrshire, Danish red, Swedish Red-and-White |
| 26 | Association | QTL:100091 | Milk fat yield | 22801092-22801096 | Ayrshire, Danish red, Swedish Red-and-White |
| 26 | Association | QTL:100093 | Milk fat yield | 22836226-22836230 | Ayrshire, Danish red, Swedish Red-and-White |
| 26 | Association | QTL:100083 | Milk fat yield | 22879215-22879219 | Ayrshire, Danish red, Swedish Red-and-White |
| 26 | Association | QTL:99962 | Milk fat yield | 22659814-22659818 | Ayrshire, Danish red, Swedish Red-and-White |
| 26 | Association | QTL:100094 | Milk fat yield | 22822006-22822010 | Ayrshire, Danish red, Swedish Red-and-White |
| 26 | Association | QTL:99994 | Milk fat yield | 22991662-22991666 | Ayrshire, Danish red, Swedish Red-and-White |
| 26 | Association | QTL:100023 | Milk fat yield | 22956885-22956889 | Ayrshire, Danish red, Swedish Red-and-White |
| 26 | Association | QTL:100206 | Milk fat yield | 23071161-23071165 | Ayrshire, Danish red, Swedish Red-and-White |
| 26 | Association | QTL:100080 | Milk fat yield | 22770529-22770533 | Ayrshire, Danish red, Swedish Red-and-White |
| 26 | Association | QTL:100340 | Milk fat yield | 23078827-23078831 | Ayrshire, Danish red, Swedish Red-and-White |
| 26 | Association | QTL:100047 | Milk fat yield | 22931547-22931551 | Ayrshire, Danish red, Swedish Red-and-White |
| 26 | Association | QTL:99980 | Milk fat yield | 23054089-23054093 | Ayrshire, Danish red, Swedish Red-and-White |
| 26 | Association | QTL:100038 | Milk fat yield | 22943609-22943613 | Ayrshire, Danish red, Swedish Red-and-White |
| 26 | Association | QTL:100064 | Milk fat yield | 22888963-22888967 | Ayrshire, Danish red, Swedish Red-and-White |
| 26 | Association | QTL:99974 | Milk fat yield | 22780547-22780551 | Ayrshire, Danish red, Swedish Red-and-White |
| 26 | Association | QTL:100053 | Milk fat yield | 23058677-23058681 | Ayrshire, Danish red, Swedish Red-and-White |
| 26 | Association | QTL:99868 | Milk fat yield | 22719510-22719514 | Ayrshire, Danish red, Swedish Red-and-White |
| 26 | Association | QTL:100042 | Milk fat yield | 23028685-23028689 | Ayrshire, Danish red, Swedish Red-and-White |
| 26 | Association | QTL:100124 | Milk fat yield | 23069087-23069091 | Ayrshire, Danish red, Swedish Red-and-White |
| 26 | Association | QTL:100092 | Milk fat yield | 22835291-22835295 | Ayrshire, Danish red, Swedish Red-and-White |
| 26 | Association | QTL:100013 | Milk fat yield | 22974553-22974557 | Ayrshire, Danish red, Swedish Red-and-White |
| 26 | Association | QTL:99984 | Milk fat yield | 22898739-22898743 | Ayrshire, Danish red, Swedish Red-and-White |
| 26 | Association | QTL:100060 | Milk fat yield | 22883692-22883696 | Ayrshire, Danish red, Swedish Red-and-White |
| 26 | Association | QTL:100089 | Milk fat yield | 22801095-22801099 | Ayrshire, Danish red, Swedish Red-and-White |
| 26 | Association | QTL:100029 | Milk fat yield | 23003689-23003693 | Ayrshire, Danish red, Swedish Red-and-White |
| 26 | Association | QTL:99966 | Milk fat yield | 23114331-23114335 | Ayrshire, Danish red, Swedish Red-and-White |
| 26 | Association | QTL:100039 | Milk fat yield | 22944925-22944929 | Ayrshire, Danish red, Swedish Red-and-White |
| 26 | Association | QTL:100192 | Milk fat yield | 23201081-23201085 | Ayrshire, Danish red, Swedish Red-and-White |
| 26 | Association | QTL:100051 | Milk fat yield | 22928664-22928668 | Ayrshire, Danish red, Swedish Red-and-White |
| 26 | Association | QTL:100009 | Milk fat yield | 22896623-22896627 | Ayrshire, Danish red, Swedish Red-and-White |
| 26 | Association | QTL:100015 | Milk fat yield | 22977097-22977101 | Ayrshire, Danish red, Swedish Red-and-White |
| 26 | Association | QTL:100085 | Milk fat yield | 22811500-22811504 | Ayrshire, Danish red, Swedish Red-and-White |
| 26 | Association | QTL:100058 | Milk fat yield | 22916813-22916817 | Ayrshire, Danish red, Swedish Red-and-White |
| 26 | Association | QTL:100021 | Milk fat yield | 22956755-22956759 | Ayrshire, Danish red, Swedish Red-and-White |
| 26 | Association | QTL:100012 | Milk fat yield | 22899671-22899675 | Ayrshire, Danish red, Swedish Red-and-White |
| 26 | Association | QTL:100028 | Milk fat yield | 23017918-23017922 | Ayrshire, Danish red, Swedish Red-and-White |
| 26 | Association | QTL:100102 | Milk fat yield | 23067588-23067592 | Ayrshire, Danish red, Swedish Red-and-White |
| 26 | Association | QTL:99808 | Milk fat yield | 23190836-23190840 | Ayrshire, Danish red, Swedish Red-and-White |
| 26 | Association | QTL:99724 | Milk fat yield | 23122923-23122927 | Ayrshire, Danish red, Swedish Red-and-White |
| 26 | Association | QTL:100071 | Milk fat yield | 22802629-22802633 | Ayrshire, Danish red, Swedish Red-and-White |
| 26 | Association | QTL:100054 | Milk fat yield | 22919185-22919189 | Ayrshire, Danish red, Swedish Red-and-White |
| 26 | Association | QTL:99733 | Milk fat yield | 23122482-23122486 | Ayrshire, Danish red, Swedish Red-and-White |
| 26 | Association | QTL:100405 | Milk fat yield | 23081331-23081335 | Ayrshire, Danish red, Swedish Red-and-White |
| 26 | Association | QTL:100435 | Milk fat yield | 22304072-22304076 | Ayrshire, Danish red, Swedish Red-and-White |
| 26 | Association | QTL:99626 | Milk fat yield | 23130413-23130417 | Ayrshire, Danish red, Swedish Red-and-White |
| 26 | Association | QTL:100026 | Milk fat yield | 23009702-23009706 | Ayrshire, Danish red, Swedish Red-and-White |
| 26 | Association | QTL:100063 | Milk fat yield | 22887105-22887109 | Ayrshire, Danish red, Swedish Red-and-White |
| 26 | Association | QTL:100081 | Milk fat yield | 23222229-23222233 | Ayrshire, Danish red, Swedish Red-and-White |
| 26 | Association | QTL:100032 | Milk fat yield | 22971051-22971055 | Ayrshire, Danish red, Swedish Red-and-White |
| 26 | Association | QTL:100068 | Milk fat yield | 22777942-22777946 | Ayrshire, Danish red, Swedish Red-and-White |
| 26 | Association | QTL:100017 | Milk fat yield | 22981456-22981460 | Ayrshire, Danish red, Swedish Red-and-White |
| 26 | Association | QTL:100052 | Milk fat yield | 22935341-22935345 | Ayrshire, Danish red, Swedish Red-and-White |
| 26 | Association | QTL:100020 | Milk fat yield | 22985122-22985126 | Ayrshire, Danish red, Swedish Red-and-White |
| 26 | Association | QTL:100025 | Milk fat yield | 22964679-22964683 | Ayrshire, Danish red, Swedish Red-and-White |
| 26 | Association | QTL:100095 | Milk fat yield | 22826321-22826325 | Ayrshire, Danish red, Swedish Red-and-White |
| 26 | Association | QTL:99869 | Milk fat yield | 22710351-22710355 | Ayrshire, Danish red, Swedish Red-and-White |
| 26 | Association | QTL:100031 | Milk fat yield | 23013011-23013015 | Ayrshire, Danish red, Swedish Red-and-White |
| 26 | Association | QTL:100084 | Milk fat yield | 23066730-23066734 | Ayrshire, Danish red, Swedish Red-and-White |
| 26 | Association | QTL:100075 | Milk fat yield | 22768645-22768649 | Ayrshire, Danish red, Swedish Red-and-White |
| 26 | Association | QTL:100030 | Milk fat yield | 23013907-23013911 | Ayrshire, Danish red, Swedish Red-and-White |
| 26 | Association | QTL:100033 | Milk fat yield | 22949402-22949406 | Ayrshire, Danish red, Swedish Red-and-White |
| 26 | Association | QTL:100011 | Milk fat yield | 22878718-22878722 | Ayrshire, Danish red, Swedish Red-and-White |
| 26 | Association | QTL:100315 | Milk fat yield | 22467084-22467088 | Ayrshire, Danish red, Swedish Red-and-White |
| 26 | Association | QTL:100074 | Milk fat yield | 22805657-22805661 | Ayrshire, Danish red, Swedish Red-and-White |
| 26 | Association | QTL:100172 | Milk fat yield | 23069775-23069779 | Ayrshire, Danish red, Swedish Red-and-White |
| 26 | Association | QTL:100003 | Milk fat yield | 23006575-23006579 | Ayrshire, Danish red, Swedish Red-and-White |
| 26 | Association | QTL:100059 | Milk fat yield | 22911152-22911156 | Ayrshire, Danish red, Swedish Red-and-White |
| 26 | Association | QTL:100044 | Milk fat yield | 22936348-22936352 | Ayrshire, Danish red, Swedish Red-and-White |
| 26 | Association | QTL:100161 | Milk fat yield | 22486041-22486045 | Ayrshire, Danish red, Swedish Red-and-White |
| 26 | Association | QTL:100264 | Milk fat yield | 23062582-23062586 | Ayrshire, Danish red, Swedish Red-and-White |
| 26 | Association | QTL:100077 | Milk fat yield | 22805223-22805227 | Ayrshire, Danish red, Swedish Red-and-White |
| 26 | Association | QTL:99854 | Milk fat yield | 21486897-21486901 | Ayrshire, Danish red, Swedish Red-and-White |
| 26 | Association | QTL:99815 | Milk fat yield | 23198664-23198668 | Ayrshire, Danish red, Swedish Red-and-White |
| 26 | Association | QTL:99851 | Milk fat yield | 21486686-21486690 | Ayrshire, Danish red, Swedish Red-and-White |
| 26 | QTL | QTL:10191 | Milk fat yield | 7008275-42377141 | Holstein |
| 26 | QTL | QTL:12179 | Myristic acid content | 0-28819076 | Jersey, Limousin |
| 26 | QTL | QTL:4648 | Udder attachment | 3336463-34144055 |  |
| 26 | QTL | QTL:1649 | Body form composite index | 0-27278565 |  |
| 26 | QTL | QTL:12180 | Saturated fatty acid content | 3274895-22924265 | Jersey, Limousin |
| 26 | QTL | QTL:2735 | Milk fat percentage | 3336463-27273325 |  |
| 26 | QTL | QTL:2572 | Milk fat yield | 14974129-27273325 |  |
| 26 | QTL | QTL:1359 | Retail product yield | 1859485-27273325 |  |
| 26 | QTL | QTL:1360 | Fat percentage | 1859485-27273325 |  |
| 26 | QTL | QTL:1361 | Yield grade | 1859485-27273325 |  |
| 26 | QTL | QTL:11228 | Body height | 10119425-27823507 | Angus |
| 26 | QTL | QTL:11227 | Calving ease | 17651684-34353648 | Angus |
| 26 | QTL | QTL:12182 | Myristoleic acid content | 0-36023845 | Jersey, Limousin |
| 26 | QTL | QTL:11333 | Milk protein yield | 20730085-24646859 | Holstein |
| 26 | QTL | QTL:11334 | Milk yield | 20730085-24646859 | Holstein |
| 26 | QTL | QTL:11332 | Milk fat percentage | 20730085-24646859 | Holstein |
| 26 | QTL | QTL:11331 | Milk fat yield | 20730085-24646859 | Holstein |
| 26 | QTL | QTL:16063 | Stillbirth | 20730085-24646859 | Jersey |
| 26 | QTL | QTL:10453 | Milk fat yield | 9890182-23448248 | Holstein |
| 26 | QTL | QTL:10452 | Milk yield | 5632819-22924265 | Holstein |
| 26 | QTL | QTL:56650 | Milk capric acid content | 1419676-38996499 | Holstein |
| 26 | QTL | QTL:56651 | Milk cis-9-C10:1 fatty acid content | 1419676-38996499 | Holstein |
| 26 | QTL | QTL:56652 | Milk lauroleic acid content | 1419676-38996499 | Holstein |
| 26 | QTL | QTL:56653 | Milk myristoleic acid content | 1419676-38996499 | Holstein |
| 26 | QTL | QTL:56654 | Milk palmitoleic acid content | 1419676-38996499 | Holstein |
| 26 | QTL | QTL:10454 | Milk protein yield | 8121739-26133662 | Holstein |
| 26 | QTL | QTL:11231 | Body weight | 19400477-27823507 | Angus |
| 26 | QTL | QTL:11378 | Stillbirth | 17651684-24646859 | Holstein |
| 26 | Association | QTL:167351 | Milk myristoleic acid content | 21410560-21410564 | Holstein |
| 26 | Association | QTL:167367 | Milk C10 index | 21410560-21410564 | Holstein |
| 26 | Association | QTL:167401 | Milk C14 index | 21410560-21410564 | Holstein |
| 26 | Association | QTL:199133 | Milk capric acid content | 21410560-21410564 | Holstein |
| 26 | Association | QTL:199134 | Milk C14 index | 21410560-21410564 | Holstein |
| 26 | Association | QTL:204181 | Milk myristoleic acid content | 21410560-21410564 | Holstein |
| 26 | Association | QTL:204182 | Milk C16 index | 21410560-21410564 | Holstein |
| 26 | Association | QTL:210431 | Milk palmitoleic acid content | 21410560-21410564 | Holstein |
| 26 | Association | QTL:197967 | Milk capric acid content | 21419518-21419522 | Holstein |
| 26 | Association | QTL:197968 | Milk myristic acid content | 21419518-21419522 | Holstein |
| 26 | Association | QTL:197969 | Milk C14 index | 21419518-21419522 | Holstein |
| 26 | Association | QTL:203160 | Milk myristoleic acid content | 21419518-21419522 | Holstein |
| 26 | Association | QTL:203161 | Milk C16 index | 21419518-21419522 | Holstein |
| 26 | Association | QTL:209514 | Milk palmitoleic acid content | 21419518-21419522 | Holstein |
| 26 | Association | QTL:199124 | Milk capric acid content | 21422548-21422552 | Holstein |
| 26 | Association | QTL:199125 | Milk myristic acid content | 21422548-21422552 | Holstein |
| 26 | Association | QTL:199126 | Milk C14 index | 21422548-21422552 | Holstein |
| 26 | Association | QTL:204175 | Milk myristoleic acid content | 21422548-21422552 | Holstein |
| 26 | Association | QTL:204176 | Milk C16 index | 21422548-21422552 | Holstein |
| 26 | Association | QTL:210428 | Milk palmitoleic acid content | 21422548-21422552 | Holstein |
| 26 | Association | QTL:196748 | Milk capric acid content | 21423654-21423658 | Holstein |
| 26 | Association | QTL:196749 | Milk myristic acid content | 21423654-21423658 | Holstein |
| 26 | Association | QTL:196750 | Milk C14 index | 21423654-21423658 | Holstein |
| 26 | Association | QTL:201994 | Milk myristoleic acid content | 21423654-21423658 | Holstein |
| 26 | Association | QTL:201995 | Milk C16 index | 21423654-21423658 | Holstein |
| 26 | Association | QTL:207993 | Milk palmitoleic acid content | 21423654-21423658 | Holstein |
| 26 | Association | QTL:199109 | Milk capric acid content | 21424738-21424742 | Holstein |
| 26 | Association | QTL:199110 | Milk myristic acid content | 21424738-21424742 | Holstein |
| 26 | Association | QTL:199111 | Milk C14 index | 21424738-21424742 | Holstein |
| 26 | Association | QTL:204165 | Milk myristoleic acid content | 21424738-21424742 | Holstein |
| 26 | Association | QTL:204166 | Milk C16 index | 21424738-21424742 | Holstein |
| 26 | Association | QTL:210423 | Milk palmitoleic acid content | 21424738-21424742 | Holstein |
| 26 | Association | QTL:197019 | Milk capric acid content | 21445477-21445481 | Holstein |
| 26 | Association | QTL:197020 | Milk myristic acid content | 21445477-21445481 | Holstein |
| 26 | Association | QTL:197021 | Milk C14 index | 21445477-21445481 | Holstein |
| 26 | Association | QTL:202238 | Milk myristoleic acid content | 21445477-21445481 | Holstein |
| 26 | Association | QTL:202239 | Milk C16 index | 21445477-21445481 | Holstein |
| 26 | Association | QTL:208319 | Milk palmitoleic acid content | 21445477-21445481 | Holstein |
| 26 | Association | QTL:167368 | Milk C10 index | 21430123-21430127 | Holstein |
| 26 | Association | QTL:167402 | Milk C14 index | 21430123-21430127 | Holstein |
| 26 | Association | QTL:199112 | Milk capric acid content | 21430123-21430127 | Holstein |
| 26 | Association | QTL:199113 | Milk myristic acid content | 21430123-21430127 | Holstein |
| 26 | Association | QTL:199114 | Milk C14 index | 21430123-21430127 | Holstein |
| 26 | Association | QTL:204167 | Milk myristoleic acid content | 21430123-21430127 | Holstein |
| 26 | Association | QTL:204168 | Milk C16 index | 21430123-21430127 | Holstein |
| 26 | Association | QTL:210424 | Milk palmitoleic acid content | 21430123-21430127 | Holstein |
| 26 | Association | QTL:199115 | Milk capric acid content | 21433894-21433898 | Holstein |
| 26 | Association | QTL:199116 | Milk myristic acid content | 21433894-21433898 | Holstein |
| 26 | Association | QTL:199117 | Milk C14 index | 21433894-21433898 | Holstein |
| 26 | Association | QTL:204169 | Milk myristoleic acid content | 21433894-21433898 | Holstein |
| 26 | Association | QTL:204170 | Milk C16 index | 21433894-21433898 | Holstein |
| 26 | Association | QTL:210425 | Milk palmitoleic acid content | 21433894-21433898 | Holstein |
| 26 | Association | QTL:199118 | Milk capric acid content | 21436479-21436483 | Holstein |
| 26 | Association | QTL:199119 | Milk myristic acid content | 21436479-21436483 | Holstein |
| 26 | Association | QTL:199120 | Milk C14 index | 21436479-21436483 | Holstein |
| 26 | Association | QTL:204171 | Milk myristoleic acid content | 21436479-21436483 | Holstein |
| 26 | Association | QTL:204172 | Milk C16 index | 21436479-21436483 | Holstein |
| 26 | Association | QTL:210426 | Milk palmitoleic acid content | 21436479-21436483 | Holstein |
| 26 | Association | QTL:196843 | Milk capric acid content | 21439534-21439538 | Holstein |
| 26 | Association | QTL:196844 | Milk myristic acid content | 21439534-21439538 | Holstein |
| 26 | Association | QTL:196845 | Milk C14 index | 21439534-21439538 | Holstein |
| 26 | Association | QTL:202079 | Milk myristoleic acid content | 21439534-21439538 | Holstein |
| 26 | Association | QTL:202080 | Milk C16 index | 21439534-21439538 | Holstein |
| 26 | Association | QTL:208098 | Milk palmitoleic acid content | 21439534-21439538 | Holstein |
| 26 | Association | QTL:198035 | Milk capric acid content | 21442687-21442691 | Holstein |
| 26 | Association | QTL:198036 | Milk myristic acid content | 21442687-21442691 | Holstein |
| 26 | Association | QTL:198037 | Milk C14 index | 21442687-21442691 | Holstein |
| 26 | Association | QTL:203227 | Milk myristoleic acid content | 21442687-21442691 | Holstein |
| 26 | Association | QTL:203228 | Milk C16 index | 21442687-21442691 | Holstein |
| 26 | Association | QTL:209617 | Milk palmitoleic acid content | 21442687-21442691 | Holstein |
| 26 | Association | QTL:199121 | Milk capric acid content | 21438247-21438251 | Holstein |
| 26 | Association | QTL:199122 | Milk myristic acid content | 21438247-21438251 | Holstein |
| 26 | Association | QTL:199123 | Milk C14 index | 21438247-21438251 | Holstein |
| 26 | Association | QTL:204173 | Milk myristoleic acid content | 21438247-21438251 | Holstein |
| 26 | Association | QTL:204174 | Milk C16 index | 21438247-21438251 | Holstein |
| 26 | Association | QTL:210427 | Milk palmitoleic acid content | 21438247-21438251 | Holstein |
| 26 | Association | QTL:197471 | Milk capric acid content | 21446029-21446033 | Holstein |
| 26 | Association | QTL:197472 | Milk myristic acid content | 21446029-21446033 | Holstein |
| 26 | Association | QTL:197473 | Milk C14 index | 21446029-21446033 | Holstein |
| 26 | Association | QTL:202689 | Milk myristoleic acid content | 21446029-21446033 | Holstein |
| 26 | Association | QTL:202690 | Milk C16 index | 21446029-21446033 | Holstein |
| 26 | Association | QTL:208914 | Milk palmitoleic acid content | 21446029-21446033 | Holstein |
| 26 | Association | QTL:199106 | Milk capric acid content | 21444069-21444073 | Holstein |
| 26 | Association | QTL:199107 | Milk myristic acid content | 21444069-21444073 | Holstein |
| 26 | Association | QTL:199108 | Milk C14 index | 21444069-21444073 | Holstein |
| 26 | Association | QTL:204163 | Milk myristoleic acid content | 21444069-21444073 | Holstein |
| 26 | Association | QTL:204164 | Milk C16 index | 21444069-21444073 | Holstein |
| 26 | Association | QTL:210422 | Milk palmitoleic acid content | 21444069-21444073 | Holstein |
| 26 | Association | QTL:167352 | Milk myristoleic acid content | 21455314-21455318 | Holstein |
| 26 | Association | QTL:167369 | Milk C10 index | 21455314-21455318 | Holstein |
| 26 | Association | QTL:167403 | Milk C14 index | 21455314-21455318 | Holstein |
| 26 | Association | QTL:196031 | Milk capric acid content | 21455314-21455318 | Holstein |
| 26 | Association | QTL:196032 | Milk C14 index | 21455314-21455318 | Holstein |
| 26 | Association | QTL:201221 | Milk myristoleic acid content | 21455314-21455318 | Holstein |
| 26 | Association | QTL:201222 | Milk C16 index | 21455314-21455318 | Holstein |
| 26 | Association | QTL:206801 | Milk palmitoleic acid content | 21455314-21455318 | Holstein |
| 26 | Association | QTL:196491 | Milk capric acid content | 21456115-21456119 | Holstein |
| 26 | Association | QTL:196492 | Milk myristic acid content | 21456115-21456119 | Holstein |
| 26 | Association | QTL:196493 | Milk C14 index | 21456115-21456119 | Holstein |
| 26 | Association | QTL:201780 | Milk myristoleic acid content | 21456115-21456119 | Holstein |
| 26 | Association | QTL:201781 | Milk C16 index | 21456115-21456119 | Holstein |
| 26 | Association | QTL:207692 | Milk palmitoleic acid content | 21456115-21456119 | Holstein |
| 26 | Association | QTL:197598 | Milk capric acid content | 21453883-21453887 | Holstein |
| 26 | Association | QTL:197599 | Milk myristic acid content | 21453883-21453887 | Holstein |
| 26 | Association | QTL:197600 | Milk C14 index | 21453883-21453887 | Holstein |
| 26 | Association | QTL:202804 | Milk myristoleic acid content | 21453883-21453887 | Holstein |
| 26 | Association | QTL:202805 | Milk C16 index | 21453883-21453887 | Holstein |
| 26 | Association | QTL:209061 | Milk palmitoleic acid content | 21453883-21453887 | Holstein |
| 26 | Association | QTL:196932 | Milk capric acid content | 21464176-21464180 | Holstein |
| 26 | Association | QTL:196933 | Milk C14 index | 21464176-21464180 | Holstein |
| 26 | Association | QTL:202178 | Milk myristoleic acid content | 21464176-21464180 | Holstein |
| 26 | Association | QTL:202179 | Milk C16 index | 21464176-21464180 | Holstein |
| 26 | Association | QTL:208239 | Milk palmitoleic acid content | 21464176-21464180 | Holstein |
| 26 | Association | QTL:197340 | Milk capric acid content | 21460937-21460941 | Holstein |
| 26 | Association | QTL:197341 | Milk C14 index | 21460937-21460941 | Holstein |
| 26 | Association | QTL:202555 | Milk myristoleic acid content | 21460937-21460941 | Holstein |
| 26 | Association | QTL:202556 | Milk C16 index | 21460937-21460941 | Holstein |
| 26 | Association | QTL:208758 | Milk palmitoleic acid content | 21460937-21460941 | Holstein |
| 26 | Association | QTL:197807 | Milk capric acid content | 21463260-21463264 | Holstein |
| 26 | Association | QTL:197808 | Milk C14 index | 21463260-21463264 | Holstein |
| 26 | Association | QTL:203010 | Milk myristoleic acid content | 21463260-21463264 | Holstein |
| 26 | Association | QTL:203011 | Milk C16 index | 21463260-21463264 | Holstein |
| 26 | Association | QTL:209328 | Milk palmitoleic acid content | 21463260-21463264 | Holstein |
| 26 | Association | QTL:196008 | Milk capric acid content | 21476913-21476917 | Holstein |
| 26 | Association | QTL:196009 | Milk myristic acid content | 21476913-21476917 | Holstein |
| 26 | Association | QTL:196010 | Milk C14 index | 21476913-21476917 | Holstein |
| 26 | Association | QTL:201193 | Milk myristoleic acid content | 21476913-21476917 | Holstein |
| 26 | Association | QTL:201194 | Milk C16 index | 21476913-21476917 | Holstein |
| 26 | Association | QTL:206763 | Milk palmitoleic acid content | 21476913-21476917 | Holstein |
| 26 | Association | QTL:206764 | Milk C18 index | 21476913-21476917 | Holstein |
| 26 | Association | QTL:196647 | Milk capric acid content | 21474306-21474310 | Holstein |
| 26 | Association | QTL:196648 | Milk myristic acid content | 21474306-21474310 | Holstein |
| 26 | Association | QTL:196649 | Milk C14 index | 21474306-21474310 | Holstein |
| 26 | Association | QTL:201908 | Milk myristoleic acid content | 21474306-21474310 | Holstein |
| 26 | Association | QTL:201909 | Milk C16 index | 21474306-21474310 | Holstein |
| 26 | Association | QTL:207891 | Milk palmitoleic acid content | 21474306-21474310 | Holstein |
| 26 | Association | QTL:207892 | Milk C18 index | 21474306-21474310 | Holstein |
| 26 | Association | QTL:195794 | Milk C14 index | 21484884-21484888 | Holstein |
| 26 | Association | QTL:200917 | Milk myristoleic acid content | 21484884-21484888 | Holstein |
| 26 | Association | QTL:200918 | Milk C16 index | 21484884-21484888 | Holstein |
| 26 | Association | QTL:206335 | Milk palmitoleic acid content | 21484884-21484888 | Holstein |
| 26 | Association | QTL:199234 | Milk C14 index | 21483530-21483534 | Holstein |
| 26 | Association | QTL:204306 | Milk myristoleic acid content | 21483530-21483534 | Holstein |
| 26 | Association | QTL:204307 | Milk C16 index | 21483530-21483534 | Holstein |
| 26 | Association | QTL:210485 | Milk palmitoleic acid content | 21483530-21483534 | Holstein |
| 26 | Association | QTL:199278 | Milk C14 index | 21482459-21482463 | Holstein |
| 26 | Association | QTL:204363 | Milk myristoleic acid content | 21482459-21482463 | Holstein |
| 26 | Association | QTL:204364 | Milk C16 index | 21482459-21482463 | Holstein |
| 26 | Association | QTL:210507 | Milk palmitoleic acid content | 21482459-21482463 | Holstein |
| 26 | Association | QTL:195595 | Milk C14 index | 21492342-21492346 | Holstein |
| 26 | Association | QTL:200662 | Milk myristoleic acid content | 21492342-21492346 | Holstein |
| 26 | Association | QTL:200663 | Milk C16 index | 21492342-21492346 | Holstein |
| 26 | Association | QTL:205909 | Milk palmitoleic acid content | 21492342-21492346 | Holstein |
| 26 | Association | QTL:196817 | Milk C14 index | 21489948-21489952 | Holstein |
| 26 | Association | QTL:202053 | Milk myristoleic acid content | 21489948-21489952 | Holstein |
| 26 | Association | QTL:202054 | Milk C16 index | 21489948-21489952 | Holstein |
| 26 | Association | QTL:208077 | Milk palmitoleic acid content | 21489948-21489952 | Holstein |
| 26 | Association | QTL:198445 | Milk C14 index | 21488716-21488720 | Holstein |
| 26 | Association | QTL:203611 | Milk myristoleic acid content | 21488716-21488720 | Holstein |
| 26 | Association | QTL:203612 | Milk C16 index | 21488716-21488720 | Holstein |
| 26 | Association | QTL:210080 | Milk palmitoleic acid content | 21488716-21488720 | Holstein |
| 26 | Association | QTL:199232 | Milk C14 index | 21487113-21487117 | Holstein |
| 26 | Association | QTL:204302 | Milk myristoleic acid content | 21487113-21487117 | Holstein |
| 26 | Association | QTL:204303 | Milk C16 index | 21487113-21487117 | Holstein |
| 26 | Association | QTL:210483 | Milk palmitoleic acid content | 21487113-21487117 | Holstein |
| 26 | Association | QTL:199233 | Milk C14 index | 21485753-21485757 | Holstein |
| 26 | Association | QTL:204304 | Milk myristoleic acid content | 21485753-21485757 | Holstein |
| 26 | Association | QTL:204305 | Milk C16 index | 21485753-21485757 | Holstein |
| 26 | Association | QTL:210484 | Milk palmitoleic acid content | 21485753-21485757 | Holstein |
| 26 | Association | QTL:195627 | Milk C14 index | 21496108-21496112 | Holstein |
| 26 | Association | QTL:200705 | Milk myristoleic acid content | 21496108-21496112 | Holstein |
| 26 | Association | QTL:200706 | Milk C16 index | 21496108-21496112 | Holstein |
| 26 | Association | QTL:205977 | Milk palmitoleic acid content | 21496108-21496112 | Holstein |
| 26 | Association | QTL:199227 | Milk capric acid content | 21497490-21497494 | Holstein |
| 26 | Association | QTL:199228 | Milk C14 index | 21497490-21497494 | Holstein |
| 26 | Association | QTL:204296 | Milk myristoleic acid content | 21497490-21497494 | Holstein |
| 26 | Association | QTL:204297 | Milk C16 index | 21497490-21497494 | Holstein |
| 26 | Association | QTL:210480 | Milk palmitoleic acid content | 21497490-21497494 | Holstein |
| 26 | Association | QTL:199229 | Milk C14 index | 21496766-21496770 | Holstein |
| 26 | Association | QTL:204298 | Milk myristoleic acid content | 21496766-21496770 | Holstein |
| 26 | Association | QTL:204299 | Milk C16 index | 21496766-21496770 | Holstein |
| 26 | Association | QTL:210481 | Milk palmitoleic acid content | 21496766-21496770 | Holstein |
| 26 | Association | QTL:199230 | Milk capric acid content | 21495448-21495452 | Holstein |
| 26 | Association | QTL:199231 | Milk C14 index | 21495448-21495452 | Holstein |
| 26 | Association | QTL:204300 | Milk myristoleic acid content | 21495448-21495452 | Holstein |
| 26 | Association | QTL:204301 | Milk C16 index | 21495448-21495452 | Holstein |
| 26 | Association | QTL:210482 | Milk palmitoleic acid content | 21495448-21495452 | Holstein |
| 26 | Association | QTL:152384 | Saturated fatty acid content | 21494661-21494665 | Angus |
| 26 | Association | QTL:167370 | Milk C10 index | 21494661-21494665 | Holstein |
| 26 | Association | QTL:167404 | Milk C14 index | 21494661-21494665 | Holstein |
| 26 | Association | QTL:199220 | Milk capric acid content | 21505508-21505512 | Holstein |
| 26 | Association | QTL:199221 | Milk myristic acid content | 21505508-21505512 | Holstein |
| 26 | Association | QTL:199222 | Milk C14 index | 21505508-21505512 | Holstein |
| 26 | Association | QTL:204290 | Milk myristoleic acid content | 21505508-21505512 | Holstein |
| 26 | Association | QTL:204291 | Milk C16 index | 21505508-21505512 | Holstein |
| 26 | Association | QTL:210477 | Milk palmitoleic acid content | 21505508-21505512 | Holstein |
| 26 | Association | QTL:199223 | Milk capric acid content | 21503646-21503650 | Holstein |
| 26 | Association | QTL:199224 | Milk C14 index | 21503646-21503650 | Holstein |
| 26 | Association | QTL:204292 | Milk myristoleic acid content | 21503646-21503650 | Holstein |
| 26 | Association | QTL:204293 | Milk C16 index | 21503646-21503650 | Holstein |
| 26 | Association | QTL:210478 | Milk palmitoleic acid content | 21503646-21503650 | Holstein |
| 26 | Association | QTL:199225 | Milk capric acid content | 21500807-21500811 | Holstein |
| 26 | Association | QTL:199226 | Milk C14 index | 21500807-21500811 | Holstein |
| 26 | Association | QTL:204294 | Milk myristoleic acid content | 21500807-21500811 | Holstein |
| 26 | Association | QTL:204295 | Milk C16 index | 21500807-21500811 | Holstein |
| 26 | Association | QTL:210479 | Milk palmitoleic acid content | 21500807-21500811 | Holstein |
| 26 | Association | QTL:112592 | Milk kappa-casein percentage | 21629772-21629776 | Holstein |
| 26 | Association | QTL:119317 | Milk unglycosylated kappa-casein percentage | 21629772-21629776 | Holstein |
| 26 | Association | QTL:112593 | Milk kappa-casein percentage | 21635616-21635620 | Holstein |
| 26 | Association | QTL:119318 | Milk unglycosylated kappa-casein percentage | 21635616-21635620 | Holstein |
| 26 | Association | QTL:112595 | Milk kappa-casein percentage | 21641638-21641642 | Holstein |
| 26 | Association | QTL:119320 | Milk unglycosylated kappa-casein percentage | 21641638-21641642 | Holstein |
| 26 | Association | QTL:167371 | Milk C10 index | 21516641-21516645 | Holstein |
| 26 | Association | QTL:167405 | Milk C14 index | 21516641-21516645 | Holstein |
| 26 | Association | QTL:199218 | Milk capric acid content | 21516641-21516645 | Holstein |
| 26 | Association | QTL:199219 | Milk C14 index | 21516641-21516645 | Holstein |
| 26 | Association | QTL:204288 | Milk myristoleic acid content | 21516641-21516645 | Holstein |
| 26 | Association | QTL:204289 | Milk C16 index | 21516641-21516645 | Holstein |
| 26 | Association | QTL:210476 | Milk palmitoleic acid content | 21516641-21516645 | Holstein |
| 26 | Association | QTL:112596 | Milk kappa-casein percentage | 21645436-21645440 | Holstein |
| 26 | Association | QTL:119321 | Milk unglycosylated kappa-casein percentage | 21645436-21645440 | Holstein |
| 26 | Association | QTL:199215 | Milk capric acid content | 21525783-21525787 | Holstein |
| 26 | Association | QTL:199216 | Milk myristic acid content | 21525783-21525787 | Holstein |
| 26 | Association | QTL:199217 | Milk C14 index | 21525783-21525787 | Holstein |
| 26 | Association | QTL:204286 | Milk myristoleic acid content | 21525783-21525787 | Holstein |
| 26 | Association | QTL:204287 | Milk C16 index | 21525783-21525787 | Holstein |
| 26 | Association | QTL:210475 | Milk palmitoleic acid content | 21525783-21525787 | Holstein |
| 26 | Association | QTL:112597 | Milk kappa-casein percentage | 21662778-21662782 | Holstein |
| 26 | Association | QTL:119322 | Milk unglycosylated kappa-casein percentage | 21662778-21662782 | Holstein |
| 26 | Association | QTL:112598 | Milk kappa-casein percentage | 21663507-21663511 | Holstein |
| 26 | Association | QTL:119323 | Milk unglycosylated kappa-casein percentage | 21663507-21663511 | Holstein |
| 26 | Association | QTL:199193 | Milk capric acid content | 21541162-21541166 | Holstein |
| 26 | Association | QTL:199194 | Milk myristic acid content | 21541162-21541166 | Holstein |
| 26 | Association | QTL:199195 | Milk C14 index | 21541162-21541166 | Holstein |
| 26 | Association | QTL:204258 | Milk myristoleic acid content | 21541162-21541166 | Holstein |
| 26 | Association | QTL:204259 | Milk C16 index | 21541162-21541166 | Holstein |
| 26 | Association | QTL:210465 | Milk palmitoleic acid content | 21541162-21541166 | Holstein |
| 26 | Association | QTL:167347 | Milk C14 index | 21539985-21539989 | Simmental |
| 26 | Association | QTL:167353 | Milk myristoleic acid content | 21539985-21539989 | Holstein |
| 26 | Association | QTL:167372 | Milk C10 index | 21539985-21539989 | Holstein |
| 26 | Association | QTL:167406 | Milk C14 index | 21539985-21539989 | Holstein |
| 26 | Association | QTL:199196 | Milk capric acid content | 21539985-21539989 | Holstein |
| 26 | Association | QTL:199197 | Milk myristic acid content | 21539985-21539989 | Holstein |
| 26 | Association | QTL:199198 | Milk C14 index | 21539985-21539989 | Holstein |
| 26 | Association | QTL:204260 | Milk myristoleic acid content | 21539985-21539989 | Holstein |
| 26 | Association | QTL:204261 | Milk C16 index | 21539985-21539989 | Holstein |
| 26 | Association | QTL:210466 | Milk palmitoleic acid content | 21539985-21539989 | Holstein |
| 26 | Association | QTL:112599 | Milk kappa-casein percentage | 21669169-21669173 | Holstein |
| 26 | Association | QTL:119324 | Milk unglycosylated kappa-casein percentage | 21669169-21669173 | Holstein |
| 26 | Association | QTL:197862 | Milk capric acid content | 21557217-21557221 | Holstein |
| 26 | Association | QTL:197863 | Milk myristic acid content | 21557217-21557221 | Holstein |
| 26 | Association | QTL:197864 | Milk C14 index | 21557217-21557221 | Holstein |
| 26 | Association | QTL:203056 | Milk myristoleic acid content | 21557217-21557221 | Holstein |
| 26 | Association | QTL:203057 | Milk C16 index | 21557217-21557221 | Holstein |
| 26 | Association | QTL:209404 | Milk palmitoleic acid content | 21557217-21557221 | Holstein |
| 26 | Association | QTL:209405 | Milk C18 index | 21557217-21557221 | Holstein |
| 26 | Association | QTL:34592 | Milk capric acid content | 21412700-21412704 | Jersey |
| 26 | Association | QTL:32910 | Milk C14 index | 21410560-21410564 | Holstein |
| 26 | Association | QTL:33561 | Milk myristoleic acid content | 21410560-21410564 | Holstein |
| 26 | Association | QTL:62448 | Milk C14 index | 21410560-21410564 | Holstein |
| 26 | Association | QTL:62581 | Milk myristoleic acid content | 21410560-21410564 | Holstein |
| 26 | Association | QTL:62898 | Milk C14 index | 21410560-21410564 | Holstein |
| 26 | Association | QTL:63033 | Milk myristoleic acid content | 21410560-21410564 | Holstein |
| 26 | Association | QTL:32839 | Milk C14 index | 21423654-21423658 | Holstein |
| 26 | Association | QTL:33324 | Milk myristoleic acid content | 21423654-21423658 | Holstein |
| 26 | Association | QTL:62440 | Milk C14 index | 21423654-21423658 | Holstein |
| 26 | Association | QTL:62538 | Milk myristoleic acid content | 21423654-21423658 | Holstein |
| 26 | Association | QTL:62886 | Milk C14 index | 21423654-21423658 | Holstein |
| 26 | Association | QTL:62965 | Milk myristoleic acid content | 21423654-21423658 | Holstein |
| 26 | Association | QTL:33006 | Milk C14 index | 21419518-21419522 | Holstein |
| 26 | Association | QTL:62459 | Milk C14 index | 21419518-21419522 | Holstein |
| 26 | Association | QTL:62588 | Milk myristoleic acid content | 21419518-21419522 | Holstein |
| 26 | Association | QTL:62927 | Milk C14 index | 21419518-21419522 | Holstein |
| 26 | Association | QTL:63068 | Milk myristoleic acid content | 21419518-21419522 | Holstein |
| 26 | Association | QTL:32870 | Milk C14 index | 21422548-21422552 | Holstein |
| 26 | Association | QTL:33502 | Milk myristoleic acid content | 21422548-21422552 | Holstein |
| 26 | Association | QTL:62460 | Milk C14 index | 21422548-21422552 | Holstein |
| 26 | Association | QTL:62590 | Milk myristoleic acid content | 21422548-21422552 | Holstein |
| 26 | Association | QTL:62892 | Milk C14 index | 21422548-21422552 | Holstein |
| 26 | Association | QTL:63018 | Milk myristoleic acid content | 21422548-21422552 | Holstein |
| 26 | Association | QTL:32840 | Milk C14 index | 21424738-21424742 | Holstein |
| 26 | Association | QTL:33325 | Milk myristoleic acid content | 21424738-21424742 | Holstein |
| 26 | Association | QTL:62441 | Milk C14 index | 21424738-21424742 | Holstein |
| 26 | Association | QTL:62548 | Milk myristoleic acid content | 21424738-21424742 | Holstein |
| 26 | Association | QTL:62887 | Milk C14 index | 21424738-21424742 | Holstein |
| 26 | Association | QTL:62974 | Milk myristoleic acid content | 21424738-21424742 | Holstein |
| 26 | Association | QTL:32992 | Milk C14 index | 21430123-21430127 | Holstein |
| 26 | Association | QTL:62461 | Milk C14 index | 21430123-21430127 | Holstein |
| 26 | Association | QTL:62591 | Milk myristoleic acid content | 21430123-21430127 | Holstein |
| 26 | Association | QTL:62920 | Milk C14 index | 21430123-21430127 | Holstein |
| 26 | Association | QTL:63060 | Milk myristoleic acid content | 21430123-21430127 | Holstein |
| 26 | Association | QTL:32841 | Milk C14 index | 21433894-21433898 | Holstein |
| 26 | Association | QTL:33326 | Milk myristoleic acid content | 21433894-21433898 | Holstein |
| 26 | Association | QTL:62439 | Milk C14 index | 21433894-21433898 | Holstein |
| 26 | Association | QTL:62544 | Milk myristoleic acid content | 21433894-21433898 | Holstein |
| 26 | Association | QTL:62885 | Milk C14 index | 21433894-21433898 | Holstein |
| 26 | Association | QTL:62967 | Milk myristoleic acid content | 21433894-21433898 | Holstein |
| 26 | Association | QTL:32993 | Milk C14 index | 21436479-21436483 | Holstein |
| 26 | Association | QTL:62462 | Milk C14 index | 21436479-21436483 | Holstein |
| 26 | Association | QTL:62592 | Milk myristoleic acid content | 21436479-21436483 | Holstein |
| 26 | Association | QTL:62921 | Milk C14 index | 21436479-21436483 | Holstein |
| 26 | Association | QTL:63061 | Milk myristoleic acid content | 21436479-21436483 | Holstein |
| 26 | QTL | QTL:5372 | Gestation length | 13492567-36089342 | Holstein, Jersey |
| 26 | Association | QTL:32995 | Milk C14 index | 21439534-21439538 | Holstein |
| 26 | Association | QTL:62464 | Milk C14 index | 21439534-21439538 | Holstein |
| 26 | Association | QTL:62594 | Milk myristoleic acid content | 21439534-21439538 | Holstein |
| 26 | Association | QTL:62923 | Milk C14 index | 21439534-21439538 | Holstein |
| 26 | Association | QTL:63063 | Milk myristoleic acid content | 21439534-21439538 | Holstein |
| 26 | Association | QTL:32996 | Milk C14 index | 21442687-21442691 | Holstein |
| 26 | Association | QTL:62465 | Milk C14 index | 21442687-21442691 | Holstein |
| 26 | Association | QTL:62595 | Milk myristoleic acid content | 21442687-21442691 | Holstein |
| 26 | Association | QTL:62924 | Milk C14 index | 21442687-21442691 | Holstein |
| 26 | Association | QTL:63064 | Milk myristoleic acid content | 21442687-21442691 | Holstein |
| 26 | Association | QTL:32997 | Milk C14 index | 21444069-21444073 | Holstein |
| 26 | Association | QTL:62466 | Milk C14 index | 21444069-21444073 | Holstein |
| 26 | Association | QTL:62596 | Milk myristoleic acid content | 21444069-21444073 | Holstein |
| 26 | Association | QTL:62925 | Milk C14 index | 21444069-21444073 | Holstein |
| 26 | Association | QTL:63065 | Milk myristoleic acid content | 21444069-21444073 | Holstein |
| 26 | Association | QTL:32994 | Milk C14 index | 21438247-21438251 | Holstein |
| 26 | Association | QTL:62463 | Milk C14 index | 21438247-21438251 | Holstein |
| 26 | Association | QTL:62593 | Milk myristoleic acid content | 21438247-21438251 | Holstein |
| 26 | Association | QTL:62922 | Milk C14 index | 21438247-21438251 | Holstein |
| 26 | Association | QTL:63062 | Milk myristoleic acid content | 21438247-21438251 | Holstein |
| 26 | Association | QTL:32527 | Milk myristoleic acid content | 21455314-21455318 | Holstein |
| 26 | Association | QTL:32572 | Milk C14 index | 21455314-21455318 | Holstein |
| 26 | Association | QTL:33104 | Milk C14 index | 21455314-21455318 | Holstein |
| 26 | Association | QTL:62481 | Milk C14 index | 21455314-21455318 | Holstein |
| 26 | Association | QTL:62690 | Milk myristoleic acid content | 21455314-21455318 | Holstein |
| 26 | Association | QTL:62954 | Milk C14 index | 21455314-21455318 | Holstein |
| 26 | Association | QTL:63173 | Milk myristoleic acid content | 21455314-21455318 | Holstein |
| 26 | Association | QTL:33003 | Milk C14 index | 21456115-21456119 | Holstein |
| 26 | Association | QTL:33827 | Milk myristoleic acid content | 21456115-21456119 | Holstein |
| 26 | Association | QTL:62446 | Milk C14 index | 21456115-21456119 | Holstein |
| 26 | Association | QTL:62608 | Milk myristoleic acid content | 21456115-21456119 | Holstein |
| 26 | Association | QTL:62893 | Milk C14 index | 21456115-21456119 | Holstein |
| 26 | Association | QTL:63051 | Milk myristoleic acid content | 21456115-21456119 | Holstein |
| 26 | Association | QTL:32924 | Milk C14 index | 21453883-21453887 | Holstein |
| 26 | Association | QTL:33689 | Milk myristoleic acid content | 21453883-21453887 | Holstein |
| 26 | Association | QTL:62478 | Milk C14 index | 21453883-21453887 | Holstein |
| 26 | Association | QTL:62662 | Milk myristoleic acid content | 21453883-21453887 | Holstein |
| 26 | Association | QTL:62907 | Milk C14 index | 21453883-21453887 | Holstein |
| 26 | Association | QTL:63071 | Milk myristoleic acid content | 21453883-21453887 | Holstein |
| 26 | Association | QTL:198383 | Milk capric acid content | 21600587-21600591 | Holstein |
| 26 | Association | QTL:198384 | Milk myristic acid content | 21600587-21600591 | Holstein |
| 26 | Association | QTL:198385 | Milk C14 index | 21600587-21600591 | Holstein |
| 26 | Association | QTL:203553 | Milk myristoleic acid content | 21600587-21600591 | Holstein |
| 26 | Association | QTL:203554 | Milk C16 index | 21600587-21600591 | Holstein |
| 26 | Association | QTL:210009 | Milk palmitoleic acid content | 21600587-21600591 | Holstein |
| 26 | Association | QTL:33106 | Milk C14 index | 21464176-21464180 | Holstein |
| 26 | Association | QTL:62543 | Milk C14 index | 21464176-21464180 | Holstein |
| 26 | Association | QTL:62722 | Milk myristoleic acid content | 21464176-21464180 | Holstein |
| 26 | Association | QTL:62990 | Milk C14 index | 21464176-21464180 | Holstein |
| 26 | Association | QTL:63204 | Milk myristoleic acid content | 21464176-21464180 | Holstein |
| 26 | Association | QTL:33244 | Milk C14 index | 21460937-21460941 | Holstein |
| 26 | Association | QTL:62541 | Milk C14 index | 21460937-21460941 | Holstein |
| 26 | Association | QTL:62720 | Milk myristoleic acid content | 21460937-21460941 | Holstein |
| 26 | Association | QTL:63015 | Milk C14 index | 21460937-21460941 | Holstein |
| 26 | Association | QTL:63234 | Milk myristoleic acid content | 21460937-21460941 | Holstein |
| 26 | Association | QTL:33105 | Milk C14 index | 21463260-21463264 | Holstein |
| 26 | Association | QTL:62542 | Milk C14 index | 21463260-21463264 | Holstein |
| 26 | Association | QTL:62721 | Milk myristoleic acid content | 21463260-21463264 | Holstein |
| 26 | Association | QTL:62989 | Milk C14 index | 21463260-21463264 | Holstein |
| 26 | Association | QTL:63203 | Milk myristoleic acid content | 21463260-21463264 | Holstein |
| 26 | Association | QTL:199181 | Milk capric acid content | 21609150-21609154 | Holstein |
| 26 | Association | QTL:199182 | Milk myristic acid content | 21609150-21609154 | Holstein |
| 26 | Association | QTL:199183 | Milk C14 index | 21609150-21609154 | Holstein |
| 26 | Association | QTL:204250 | Milk myristoleic acid content | 21609150-21609154 | Holstein |
| 26 | Association | QTL:204251 | Milk C16 index | 21609150-21609154 | Holstein |
| 26 | Association | QTL:210461 | Milk palmitoleic acid content | 21609150-21609154 | Holstein |
| 26 | Association | QTL:167354 | Milk myristoleic acid content | 21610246-21610250 | Holstein |
| 26 | Association | QTL:167373 | Milk C10 index | 21610246-21610250 | Holstein |
| 26 | Association | QTL:167407 | Milk C14 index | 21610246-21610250 | Holstein |
| 26 | Association | QTL:199184 | Milk capric acid content | 21610246-21610250 | Holstein |
| 26 | Association | QTL:199185 | Milk myristic acid content | 21610246-21610250 | Holstein |
| 26 | Association | QTL:199186 | Milk C14 index | 21610246-21610250 | Holstein |
| 26 | Association | QTL:204252 | Milk myristoleic acid content | 21610246-21610250 | Holstein |
| 26 | Association | QTL:204253 | Milk C16 index | 21610246-21610250 | Holstein |
| 26 | Association | QTL:210462 | Milk palmitoleic acid content | 21610246-21610250 | Holstein |
| 26 | Association | QTL:199187 | Milk capric acid content | 21606014-21606018 | Holstein |
| 26 | Association | QTL:199188 | Milk myristic acid content | 21606014-21606018 | Holstein |
| 26 | Association | QTL:199189 | Milk C14 index | 21606014-21606018 | Holstein |
| 26 | Association | QTL:204254 | Milk myristoleic acid content | 21606014-21606018 | Holstein |
| 26 | Association | QTL:204255 | Milk C16 index | 21606014-21606018 | Holstein |
| 26 | Association | QTL:210463 | Milk palmitoleic acid content | 21606014-21606018 | Holstein |
| 26 | Association | QTL:199190 | Milk capric acid content | 21604425-21604429 | Holstein |
| 26 | Association | QTL:199191 | Milk myristic acid content | 21604425-21604429 | Holstein |
| 26 | Association | QTL:199192 | Milk C14 index | 21604425-21604429 | Holstein |
| 26 | Association | QTL:204256 | Milk myristoleic acid content | 21604425-21604429 | Holstein |
| 26 | Association | QTL:204257 | Milk C16 index | 21604425-21604429 | Holstein |
| 26 | Association | QTL:210464 | Milk palmitoleic acid content | 21604425-21604429 | Holstein |
| 26 | Association | QTL:32998 | Milk C14 index | 21445477-21445481 | Holstein |
| 26 | Association | QTL:62456 | Milk C14 index | 21445477-21445481 | Holstein |
| 26 | Association | QTL:62602 | Milk myristoleic acid content | 21445477-21445481 | Holstein |
| 26 | Association | QTL:62916 | Milk C14 index | 21445477-21445481 | Holstein |
| 26 | Association | QTL:63069 | Milk myristoleic acid content | 21445477-21445481 | Holstein |
| 26 | Association | QTL:32999 | Milk C14 index | 21446029-21446033 | Holstein |
| 26 | Association | QTL:62457 | Milk C14 index | 21446029-21446033 | Holstein |
| 26 | Association | QTL:62603 | Milk myristoleic acid content | 21446029-21446033 | Holstein |
| 26 | Association | QTL:62917 | Milk C14 index | 21446029-21446033 | Holstein |
| 26 | Association | QTL:63070 | Milk myristoleic acid content | 21446029-21446033 | Holstein |
| 26 | Association | QTL:197700 | Milk C14 index | 21612554-21612558 | Holstein |
| 26 | Association | QTL:202903 | Milk myristoleic acid content | 21612554-21612558 | Holstein |
| 26 | Association | QTL:202904 | Milk C16 index | 21612554-21612558 | Holstein |
| 26 | Association | QTL:209203 | Milk palmitoleic acid content | 21612554-21612558 | Holstein |
| 26 | Association | QTL:32926 | Milk C14 index | 21476913-21476917 | Holstein |
| 26 | Association | QTL:33493 | Milk myristoleic acid content | 21476913-21476917 | Holstein |
| 26 | Association | QTL:62445 | Milk C14 index | 21476913-21476917 | Holstein |
| 26 | Association | QTL:62560 | Milk myristoleic acid content | 21476913-21476917 | Holstein |
| 26 | Association | QTL:62890 | Milk C14 index | 21476913-21476917 | Holstein |
| 26 | Association | QTL:63017 | Milk myristoleic acid content | 21476913-21476917 | Holstein |
| 26 | Association | QTL:32925 | Milk C14 index | 21474306-21474310 | Holstein |
| 26 | Association | QTL:33492 | Milk myristoleic acid content | 21474306-21474310 | Holstein |
| 26 | Association | QTL:62444 | Milk C14 index | 21474306-21474310 | Holstein |
| 26 | Association | QTL:62559 | Milk myristoleic acid content | 21474306-21474310 | Holstein |
| 26 | Association | QTL:62889 | Milk C14 index | 21474306-21474310 | Holstein |
| 26 | Association | QTL:63016 | Milk myristoleic acid content | 21474306-21474310 | Holstein |
| 26 | Association | QTL:152417 | Saturated fatty acid content | 21621448-21621452 | Angus |
| 26 | Association | QTL:33196 | Milk C14 index | 21484884-21484888 | Holstein |
| 26 | Association | QTL:62537 | Milk C14 index | 21484884-21484888 | Holstein |
| 26 | Association | QTL:62670 | Milk myristoleic acid content | 21484884-21484888 | Holstein |
| 26 | Association | QTL:63010 | Milk C14 index | 21484884-21484888 | Holstein |
| 26 | Association | QTL:63184 | Milk myristoleic acid content | 21484884-21484888 | Holstein |
| 26 | Association | QTL:33195 | Milk C14 index | 21483530-21483534 | Holstein |
| 26 | Association | QTL:62536 | Milk C14 index | 21483530-21483534 | Holstein |
| 26 | Association | QTL:62669 | Milk myristoleic acid content | 21483530-21483534 | Holstein |
| 26 | Association | QTL:63009 | Milk C14 index | 21483530-21483534 | Holstein |
| 26 | Association | QTL:63183 | Milk myristoleic acid content | 21483530-21483534 | Holstein |
| 26 | Association | QTL:33194 | Milk C14 index | 21482459-21482463 | Holstein |
| 26 | Association | QTL:62535 | Milk C14 index | 21482459-21482463 | Holstein |
| 26 | Association | QTL:62668 | Milk myristoleic acid content | 21482459-21482463 | Holstein |
| 26 | Association | QTL:63008 | Milk C14 index | 21482459-21482463 | Holstein |
| 26 | Association | QTL:63182 | Milk myristoleic acid content | 21482459-21482463 | Holstein |
| 26 | Association | QTL:33199 | Milk C14 index | 21489948-21489952 | Holstein |
| 26 | Association | QTL:62517 | Milk C14 index | 21489948-21489952 | Holstein |
| 26 | Association | QTL:62639 | Milk myristoleic acid content | 21489948-21489952 | Holstein |
| 26 | Association | QTL:62993 | Milk C14 index | 21489948-21489952 | Holstein |
| 26 | Association | QTL:63151 | Milk myristoleic acid content | 21489948-21489952 | Holstein |
| 26 | Association | QTL:33198 | Milk C14 index | 21488716-21488720 | Holstein |
| 26 | Association | QTL:62516 | Milk C14 index | 21488716-21488720 | Holstein |
| 26 | Association | QTL:62638 | Milk myristoleic acid content | 21488716-21488720 | Holstein |
| 26 | Association | QTL:62992 | Milk C14 index | 21488716-21488720 | Holstein |
| 26 | Association | QTL:63150 | Milk myristoleic acid content | 21488716-21488720 | Holstein |
| 26 | Association | QTL:33197 | Milk C14 index | 21485753-21485757 | Holstein |
| 26 | Association | QTL:62515 | Milk C14 index | 21485753-21485757 | Holstein |
| 26 | Association | QTL:62637 | Milk myristoleic acid content | 21485753-21485757 | Holstein |
| 26 | Association | QTL:62991 | Milk C14 index | 21485753-21485757 | Holstein |
| 26 | Association | QTL:63149 | Milk myristoleic acid content | 21485753-21485757 | Holstein |
| 26 | Association | QTL:167374 | Milk C10 index | 21638372-21638376 | Holstein |
| 26 | Association | QTL:167408 | Milk C14 index | 21638372-21638376 | Holstein |
| 26 | Association | QTL:200073 | Milk C14 index | 21638372-21638376 | Holstein |
| 26 | Association | QTL:205065 | Milk myristoleic acid content | 21638372-21638376 | Holstein |
| 26 | Association | QTL:205066 | Milk C16 index | 21638372-21638376 | Holstein |
| 26 | Association | QTL:211293 | Milk palmitoleic acid content | 21638372-21638376 | Holstein |
| 26 | Association | QTL:33200 | Milk C14 index | 21492342-21492346 | Holstein |
| 26 | Association | QTL:62518 | Milk C14 index | 21492342-21492346 | Holstein |
| 26 | Association | QTL:62640 | Milk myristoleic acid content | 21492342-21492346 | Holstein |
| 26 | Association | QTL:62994 | Milk C14 index | 21492342-21492346 | Holstein |
| 26 | Association | QTL:63152 | Milk myristoleic acid content | 21492342-21492346 | Holstein |
| 26 | Association | QTL:32984 | Milk C14 index | 21497490-21497494 | Holstein |
| 26 | Association | QTL:33763 | Milk myristoleic acid content | 21497490-21497494 | Holstein |
| 26 | Association | QTL:62526 | Milk C14 index | 21497490-21497494 | Holstein |
| 26 | Association | QTL:62759 | Milk myristoleic acid content | 21497490-21497494 | Holstein |
| 26 | Association | QTL:62970 | Milk C14 index | 21497490-21497494 | Holstein |
| 26 | Association | QTL:63191 | Milk myristoleic acid content | 21497490-21497494 | Holstein |
| 26 | Association | QTL:33211 | Milk C14 index | 21496766-21496770 | Holstein |
| 26 | Association | QTL:62531 | Milk C14 index | 21496766-21496770 | Holstein |
| 26 | Association | QTL:62707 | Milk myristoleic acid content | 21496766-21496770 | Holstein |
| 26 | Association | QTL:63000 | Milk C14 index | 21496766-21496770 | Holstein |
| 26 | Association | QTL:63206 | Milk myristoleic acid content | 21496766-21496770 | Holstein |
| 26 | Association | QTL:32983 | Milk C14 index | 21495448-21495452 | Holstein |
| 26 | Association | QTL:62525 | Milk C14 index | 21495448-21495452 | Holstein |
| 26 | Association | QTL:62758 | Milk myristoleic acid content | 21495448-21495452 | Holstein |
| 26 | Association | QTL:62969 | Milk C14 index | 21495448-21495452 | Holstein |
| 26 | Association | QTL:63190 | Milk myristoleic acid content | 21495448-21495452 | Holstein |
| 26 | Association | QTL:32525 | Milk myristoleic acid content | 21494661-21494665 | Holstein |
| 26 | Association | QTL:32570 | Milk C14 index | 21494661-21494665 | Holstein |
| 26 | Association | QTL:32871 | Milk C14 index | 21505508-21505512 | Holstein |
| 26 | Association | QTL:33503 | Milk myristoleic acid content | 21505508-21505512 | Holstein |
| 26 | Association | QTL:62530 | Milk C14 index | 21505508-21505512 | Holstein |
| 26 | Association | QTL:62733 | Milk myristoleic acid content | 21505508-21505512 | Holstein |
| 26 | Association | QTL:62915 | Milk C14 index | 21505508-21505512 | Holstein |
| 26 | Association | QTL:63108 | Milk myristoleic acid content | 21505508-21505512 | Holstein |
| 26 | Association | QTL:32986 | Milk C14 index | 21503646-21503650 | Holstein |
| 26 | Association | QTL:33765 | Milk myristoleic acid content | 21503646-21503650 | Holstein |
| 26 | Association | QTL:62527 | Milk C14 index | 21503646-21503650 | Holstein |
| 26 | Association | QTL:62760 | Milk myristoleic acid content | 21503646-21503650 | Holstein |
| 26 | Association | QTL:62971 | Milk C14 index | 21503646-21503650 | Holstein |
| 26 | Association | QTL:63192 | Milk myristoleic acid content | 21503646-21503650 | Holstein |
| 26 | Association | QTL:32985 | Milk C14 index | 21500807-21500811 | Holstein |
| 26 | Association | QTL:33764 | Milk myristoleic acid content | 21500807-21500811 | Holstein |
| 26 | Association | QTL:62533 | Milk C14 index | 21500807-21500811 | Holstein |
| 26 | Association | QTL:62768 | Milk myristoleic acid content | 21500807-21500811 | Holstein |
| 26 | Association | QTL:62975 | Milk C14 index | 21500807-21500811 | Holstein |
| 26 | Association | QTL:63202 | Milk myristoleic acid content | 21500807-21500811 | Holstein |
| 26 | Association | QTL:196879 | Milk capric acid content | 21653685-21653689 | Holstein |
| 26 | Association | QTL:196880 | Milk C14 index | 21653685-21653689 | Holstein |
| 26 | Association | QTL:202115 | Milk myristoleic acid content | 21653685-21653689 | Holstein |
| 26 | Association | QTL:202116 | Milk C16 index | 21653685-21653689 | Holstein |
| 26 | Association | QTL:208148 | Milk palmitoleic acid content | 21653685-21653689 | Holstein |
| 26 | Association | QTL:197481 | Milk capric acid content | 21681710-21681714 | Holstein |
| 26 | Association | QTL:197482 | Milk myristic acid content | 21681710-21681714 | Holstein |
| 26 | Association | QTL:197483 | Milk C14 index | 21681710-21681714 | Holstein |
| 26 | Association | QTL:202694 | Milk myristoleic acid content | 21681710-21681714 | Holstein |
| 26 | Association | QTL:202695 | Milk C16 index | 21681710-21681714 | Holstein |
| 26 | Association | QTL:208919 | Milk palmitoleic acid content | 21681710-21681714 | Holstein |
| 26 | Association | QTL:32880 | Milk C14 index | 21541162-21541166 | Holstein |
| 26 | Association | QTL:33565 | Milk myristoleic acid content | 21541162-21541166 | Holstein |
| 26 | Association | QTL:62546 | Milk C14 index | 21541162-21541166 | Holstein |
| 26 | Association | QTL:62772 | Milk myristoleic acid content | 21541162-21541166 | Holstein |
| 26 | Association | QTL:62929 | Milk C14 index | 21541162-21541166 | Holstein |
| 26 | Association | QTL:63145 | Milk myristoleic acid content | 21541162-21541166 | Holstein |
| 26 | Association | QTL:32524 | Milk myristoleic acid content | 21539985-21539989 | Holstein |
| 26 | Association | QTL:32564 | Milk C14 index | 21539985-21539989 | Holstein |
| 26 | Association | QTL:32937 | Milk C14 index | 21539985-21539989 | Holstein |
| 26 | Association | QTL:33690 | Milk myristoleic acid content | 21539985-21539989 | Holstein |
| 26 | Association | QTL:62512 | Milk C14 index | 21539985-21539989 | Holstein |
| 26 | Association | QTL:62761 | Milk myristoleic acid content | 21539985-21539989 | Holstein |
| 26 | Association | QTL:62930 | Milk C14 index | 21539985-21539989 | Holstein |
| 26 | Association | QTL:63148 | Milk myristoleic acid content | 21539985-21539989 | Holstein |
| 26 | Association | QTL:167355 | Milk myristoleic acid content | 21687729-21687733 | Holstein |
| 26 | Association | QTL:167375 | Milk C10 index | 21687729-21687733 | Holstein |
| 26 | Association | QTL:167409 | Milk C14 index | 21687729-21687733 | Holstein |
| 26 | Association | QTL:199279 | Milk capric acid content | 21687729-21687733 | Holstein |
| 26 | Association | QTL:199280 | Milk C14 index | 21687729-21687733 | Holstein |
| 26 | Association | QTL:204365 | Milk myristoleic acid content | 21687729-21687733 | Holstein |
| 26 | Association | QTL:204366 | Milk C16 index | 21687729-21687733 | Holstein |
| 26 | Association | QTL:210508 | Milk palmitoleic acid content | 21687729-21687733 | Holstein |
| 26 | Association | QTL:167356 | Milk myristoleic acid content | 21696802-21696806 | Holstein |
| 26 | Association | QTL:167376 | Milk C10 index | 21696802-21696806 | Holstein |
| 26 | Association | QTL:167410 | Milk C14 index | 21696802-21696806 | Holstein |
| 26 | Association | QTL:199281 | Milk capric acid content | 21696802-21696806 | Holstein |
| 26 | Association | QTL:199282 | Milk myristic acid content | 21696802-21696806 | Holstein |
| 26 | Association | QTL:199283 | Milk C14 index | 21696802-21696806 | Holstein |
| 26 | Association | QTL:204367 | Milk myristoleic acid content | 21696802-21696806 | Holstein |
| 26 | Association | QTL:204368 | Milk C16 index | 21696802-21696806 | Holstein |
| 26 | Association | QTL:210509 | Milk palmitoleic acid content | 21696802-21696806 | Holstein |
| 26 | Association | QTL:199284 | Milk C14 index | 21699254-21699258 | Holstein |
| 26 | Association | QTL:204369 | Milk myristoleic acid content | 21699254-21699258 | Holstein |
| 26 | Association | QTL:204370 | Milk C16 index | 21699254-21699258 | Holstein |
| 26 | Association | QTL:210510 | Milk palmitoleic acid content | 21699254-21699258 | Holstein |
| 26 | Association | QTL:107223 | Conception rate | 21827274-21827278 | Holstein |
| 26 | Association | QTL:33072 | Milk C14 index | 21557217-21557221 | Holstein |
| 26 | Association | QTL:62443 | Milk C14 index | 21557217-21557221 | Holstein |
| 26 | Association | QTL:62555 | Milk myristoleic acid content | 21557217-21557221 | Holstein |
| 26 | Association | QTL:62906 | Milk C14 index | 21557217-21557221 | Holstein |
| 26 | Association | QTL:63050 | Milk myristoleic acid content | 21557217-21557221 | Holstein |
| 26 | Association | QTL:199285 | Milk capric acid content | 21711961-21711965 | Holstein |
| 26 | Association | QTL:199286 | Milk myristic acid content | 21711961-21711965 | Holstein |
| 26 | Association | QTL:199287 | Milk C14 index | 21711961-21711965 | Holstein |
| 26 | Association | QTL:204371 | Milk myristoleic acid content | 21711961-21711965 | Holstein |
| 26 | Association | QTL:204372 | Milk C16 index | 21711961-21711965 | Holstein |
| 26 | Association | QTL:210511 | Milk palmitoleic acid content | 21711961-21711965 | Holstein |
| 26 | Association | QTL:199288 | Milk capric acid content | 21715817-21715821 | Holstein |
| 26 | Association | QTL:199289 | Milk myristic acid content | 21715817-21715821 | Holstein |
| 26 | Association | QTL:199290 | Milk C14 index | 21715817-21715821 | Holstein |
| 26 | Association | QTL:204373 | Milk myristoleic acid content | 21715817-21715821 | Holstein |
| 26 | Association | QTL:204374 | Milk C16 index | 21715817-21715821 | Holstein |
| 26 | Association | QTL:210512 | Milk palmitoleic acid content | 21715817-21715821 | Holstein |
| 26 | Association | QTL:167357 | Milk myristoleic acid content | 21730300-21730304 | Holstein |
| 26 | Association | QTL:167377 | Milk C10 index | 21730300-21730304 | Holstein |
| 26 | Association | QTL:167411 | Milk C14 index | 21730300-21730304 | Holstein |
| 26 | Association | QTL:199291 | Milk capric acid content | 21730300-21730304 | Holstein |
| 26 | Association | QTL:199292 | Milk C14 index | 21730300-21730304 | Holstein |
| 26 | Association | QTL:204375 | Milk myristoleic acid content | 21730300-21730304 | Holstein |
| 26 | Association | QTL:204376 | Milk C16 index | 21730300-21730304 | Holstein |
| 26 | Association | QTL:210513 | Milk palmitoleic acid content | 21730300-21730304 | Holstein |
| 26 | QTL | QTL:106561 | Milk yield | 21487341-22001261 | Blonde d'aquitaine |
| 26 | Association | QTL:33074 | Milk C14 index | 21606014-21606018 | Holstein |
| 26 | Association | QTL:34129 | Milk C14 index | 21606014-21606018 | Jersey |
| 26 | Association | QTL:62549 | Milk C14 index | 21606014-21606018 | Holstein |
| 26 | Association | QTL:62792 | Milk myristoleic acid content | 21606014-21606018 | Holstein |
| 26 | Association | QTL:62998 | Milk C14 index | 21606014-21606018 | Holstein |
| 26 | Association | QTL:63228 | Milk myristoleic acid content | 21606014-21606018 | Holstein |
| 26 | Association | QTL:33073 | Milk C14 index | 21604425-21604429 | Holstein |
| 26 | Association | QTL:34128 | Milk C14 index | 21604425-21604429 | Jersey |
| 26 | Association | QTL:62547 | Milk C14 index | 21604425-21604429 | Holstein |
| 26 | Association | QTL:62774 | Milk myristoleic acid content | 21604425-21604429 | Holstein |
| 26 | Association | QTL:62996 | Milk C14 index | 21604425-21604429 | Holstein |
| 26 | Association | QTL:63224 | Milk myristoleic acid content | 21604425-21604429 | Holstein |
| 26 | Association | QTL:32828 | Milk C14 index | 21610246-21610250 | Holstein |
| 26 | Association | QTL:33272 | Milk myristoleic acid content | 21610246-21610250 | Holstein |
| 26 | Association | QTL:62511 | Milk C14 index | 21610246-21610250 | Holstein |
| 26 | Association | QTL:62709 | Milk myristoleic acid content | 21610246-21610250 | Holstein |
| 26 | Association | QTL:62891 | Milk C14 index | 21610246-21610250 | Holstein |
| 26 | Association | QTL:63042 | Milk myristoleic acid content | 21610246-21610250 | Holstein |
| 26 | Association | QTL:167358 | Milk myristoleic acid content | 21761088-21761092 | Holstein |
| 26 | Association | QTL:167378 | Milk C10 index | 21761088-21761092 | Holstein |
| 26 | Association | QTL:167412 | Milk C14 index | 21761088-21761092 | Holstein |
| 26 | Association | QTL:199323 | Milk capric acid content | 21761088-21761092 | Holstein |
| 26 | Association | QTL:199324 | Milk C14 index | 21761088-21761092 | Holstein |
| 26 | Association | QTL:204415 | Milk myristoleic acid content | 21761088-21761092 | Holstein |
| 26 | Association | QTL:204416 | Milk C16 index | 21761088-21761092 | Holstein |
| 26 | Association | QTL:210535 | Milk palmitoleic acid content | 21761088-21761092 | Holstein |
| 26 | QTL | QTL:19634 | Myristoleic acid content | 21539985-21539989 | Angus |
| 26 | QTL | QTL:19639 | Palmitoleic acid content | 21539985-21539989 | Angus |
| 26 | QTL | QTL:19756 | Palmitoleic acid content | 21539985-21539989 | Angus |
| 26 | QTL | QTL:19788 | Cis-12-C18:1 fatty acid content | 21539985-21539989 | Angus |
| 26 | QTL | QTL:20519 | Cis-12-C18:1 fatty acid content | 21539985-21539989 | Angus |
| 26 | QTL | QTL:19629 | Myristic acid content | 21494661-21494665 | Angus |
| 26 | QTL | QTL:19728 | Saturated fatty acid content | 21494661-21494665 | Angus |
| 26 | QTL | QTL:19749 | Myristic acid content | 21494661-21494665 | Angus |
| 26 | QTL | QTL:20534 | Atherogenic index | 21494661-21494665 | Angus |
| 26 | Association | QTL:33151 | Milk C14 index | 21638372-21638376 | Holstein |
| 26 | Association | QTL:62476 | Milk C14 index | 21638372-21638376 | Holstein |
| 26 | Association | QTL:62663 | Milk myristoleic acid content | 21638372-21638376 | Holstein |
| 26 | Association | QTL:62966 | Milk C14 index | 21638372-21638376 | Holstein |
| 26 | Association | QTL:63153 | Milk myristoleic acid content | 21638372-21638376 | Holstein |
| 26 | Association | QTL:32951 | Milk C14 index | 21681710-21681714 | Holstein |
| 26 | Association | QTL:33610 | Milk myristoleic acid content | 21681710-21681714 | Holstein |
| 26 | Association | QTL:62451 | Milk C14 index | 21681710-21681714 | Holstein |
| 26 | Association | QTL:62693 | Milk myristoleic acid content | 21681710-21681714 | Holstein |
| 26 | Association | QTL:62912 | Milk C14 index | 21681710-21681714 | Holstein |
| 26 | Association | QTL:63094 | Milk myristoleic acid content | 21681710-21681714 | Holstein |
| 26 | Association | QTL:32878 | Milk C14 index | 21687729-21687733 | Holstein |
| 26 | Association | QTL:33497 | Milk myristoleic acid content | 21687729-21687733 | Holstein |
| 26 | Association | QTL:62453 | Milk C14 index | 21687729-21687733 | Holstein |
| 26 | Association | QTL:62699 | Milk myristoleic acid content | 21687729-21687733 | Holstein |
| 26 | Association | QTL:62899 | Milk C14 index | 21687729-21687733 | Holstein |
| 26 | Association | QTL:63072 | Milk myristoleic acid content | 21687729-21687733 | Holstein |
| 26 | Association | QTL:167413 | Milk C14 index | 21834743-21834747 | Holstein |
| 26 | Association | QTL:199325 | Milk C14 index | 21834743-21834747 | Holstein |
| 26 | Association | QTL:204417 | Milk myristoleic acid content | 21834743-21834747 | Holstein |
| 26 | Association | QTL:204418 | Milk C16 index | 21834743-21834747 | Holstein |
| 26 | Association | QTL:210536 | Milk palmitoleic acid content | 21834743-21834747 | Holstein |
| 26 | Association | QTL:32923 | Milk C14 index | 21696802-21696806 | Holstein |
| 26 | Association | QTL:33644 | Milk myristoleic acid content | 21696802-21696806 | Holstein |
| 26 | Association | QTL:62454 | Milk C14 index | 21696802-21696806 | Holstein |
| 26 | Association | QTL:62700 | Milk myristoleic acid content | 21696802-21696806 | Holstein |
| 26 | Association | QTL:62909 | Milk C14 index | 21696802-21696806 | Holstein |
| 26 | Association | QTL:63083 | Milk myristoleic acid content | 21696802-21696806 | Holstein |
| 26 | Association | QTL:33033 | Milk C14 index | 21699254-21699258 | Holstein |
| 26 | Association | QTL:33706 | Milk myristoleic acid content | 21699254-21699258 | Holstein |
| 26 | Association | QTL:62523 | Milk C14 index | 21699254-21699258 | Holstein |
| 26 | Association | QTL:62658 | Milk myristoleic acid content | 21699254-21699258 | Holstein |
| 26 | Association | QTL:62982 | Milk C14 index | 21699254-21699258 | Holstein |
| 26 | Association | QTL:63085 | Milk myristoleic acid content | 21699254-21699258 | Holstein |
| 26 | Association | QTL:32827 | Milk C14 index | 21711961-21711965 | Holstein |
| 26 | Association | QTL:33267 | Milk myristoleic acid content | 21711961-21711965 | Holstein |
| 26 | Association | QTL:62437 | Milk C14 index | 21711961-21711965 | Holstein |
| 26 | Association | QTL:62513 | Milk myristoleic acid content | 21711961-21711965 | Holstein |
| 26 | Association | QTL:62882 | Milk C14 index | 21711961-21711965 | Holstein |
| 26 | Association | QTL:62913 | Milk myristoleic acid content | 21711961-21711965 | Holstein |
| 26 | Association | QTL:32829 | Milk C14 index | 21715817-21715821 | Holstein |
| 26 | Association | QTL:33273 | Milk myristoleic acid content | 21715817-21715821 | Holstein |
| 26 | Association | QTL:62438 | Milk C14 index | 21715817-21715821 | Holstein |
| 26 | Association | QTL:62514 | Milk myristoleic acid content | 21715817-21715821 | Holstein |
| 26 | Association | QTL:62883 | Milk C14 index | 21715817-21715821 | Holstein |
| 26 | Association | QTL:62914 | Milk myristoleic acid content | 21715817-21715821 | Holstein |
| 26 | Association | QTL:33007 | Milk C14 index | 21730300-21730304 | Holstein |
| 26 | Association | QTL:33835 | Milk myristoleic acid content | 21730300-21730304 | Holstein |
| 26 | Association | QTL:62449 | Milk C14 index | 21730300-21730304 | Holstein |
| 26 | Association | QTL:62576 | Milk myristoleic acid content | 21730300-21730304 | Holstein |
| 26 | Association | QTL:62911 | Milk C14 index | 21730300-21730304 | Holstein |
| 26 | Association | QTL:63037 | Milk myristoleic acid content | 21730300-21730304 | Holstein |
| 26 | Association | QTL:166385 | Milk yield | 21932904-21932908 | Holstein, Normande, MontbÃ©liarde |
| 26 | Association | QTL:32520 | Milk myristoleic acid content | 21834743-21834747 |  |
| 26 | Association | QTL:32563 | Milk C14 index | 21834743-21834747 | Holstein |
| 26 | Association | QTL:33068 | Milk C14 index | 21834743-21834747 | Holstein |
| 26 | Association | QTL:33755 | Milk myristoleic acid content | 21834743-21834747 | Holstein |
| 26 | Association | QTL:62486 | Milk C14 index | 21834743-21834747 | Holstein |
| 26 | Association | QTL:62572 | Milk myristoleic acid content | 21834743-21834747 | Holstein |
| 26 | Association | QTL:62968 | Milk C14 index | 21834743-21834747 | Holstein |
| 26 | Association | QTL:63055 | Milk myristoleic acid content | 21834743-21834747 | Holstein |
| 26 | Association | QTL:167359 | Milk myristoleic acid content | 22010297-22010301 | Holstein |
| 26 | Association | QTL:167379 | Milk C10 index | 22010297-22010301 | Holstein |
| 26 | Association | QTL:167414 | Milk C14 index | 22010297-22010301 | Holstein |
| 26 | Association | QTL:196186 | Milk capric acid content | 22010297-22010301 | Holstein |
| 26 | Association | QTL:196187 | Milk myristic acid content | 22010297-22010301 | Holstein |
| 26 | Association | QTL:196188 | Milk C14 index | 22010297-22010301 | Holstein |
| 26 | Association | QTL:201403 | Milk myristoleic acid content | 22010297-22010301 | Holstein |
| 26 | Association | QTL:201404 | Milk C16 index | 22010297-22010301 | Holstein |
| 26 | Association | QTL:207045 | Milk palmitoleic acid content | 22010297-22010301 | Holstein |
| 26 | QTL | QTL:56582 | Milk capric acid content | 3006550-41086362 | Holstein |
| 26 | QTL | QTL:56583 | Milk myristic acid content | 3006550-41086362 | Holstein |
| 26 | QTL | QTL:56584 | Milk palmitic acid content | 3006550-41086362 | Holstein |
| 26 | QTL | QTL:56585 | Milk cis-9-C10:1 fatty acid content | 3006550-41086362 | Holstein |
| 26 | QTL | QTL:56586 | Milk lauroleic acid content | 3006550-41086362 | Holstein |
| 26 | QTL | QTL:56587 | Milk myristoleic acid content | 3006550-41086362 | Holstein |
| 26 | QTL | QTL:56588 | Milk palmitoleic acid content | 3006550-41086362 | Holstein |
| 26 | Association | QTL:197601 | Milk capric acid content | 22042818-22042822 | Holstein |
| 26 | Association | QTL:197602 | Milk myristic acid content | 22042818-22042822 | Holstein |
| 26 | Association | QTL:197603 | Milk C14 index | 22042818-22042822 | Holstein |
| 26 | Association | QTL:202806 | Milk myristoleic acid content | 22042818-22042822 | Holstein |
| 26 | Association | QTL:202807 | Milk C16 index | 22042818-22042822 | Holstein |
| 26 | Association | QTL:209062 | Milk palmitoleic acid content | 22042818-22042822 | Holstein |
| 26 | Association | QTL:195389 | Milk capric acid content | 22050193-22050197 | Holstein |
| 26 | Association | QTL:195390 | Milk myristic acid content | 22050193-22050197 | Holstein |
| 26 | Association | QTL:195391 | Milk C14 index | 22050193-22050197 | Holstein |
| 26 | Association | QTL:200390 | Milk myristoleic acid content | 22050193-22050197 | Holstein |
| 26 | Association | QTL:200391 | Milk C16 index | 22050193-22050197 | Holstein |
| 26 | Association | QTL:205485 | Milk palmitoleic acid content | 22050193-22050197 | Holstein |
| 26 | Association | QTL:167360 | Milk myristoleic acid content | 22056641-22056645 | Holstein |
| 26 | Association | QTL:167380 | Milk C10 index | 22056641-22056645 | Holstein |
| 26 | Association | QTL:167415 | Milk C14 index | 22056641-22056645 | Holstein |
| 26 | Association | QTL:195231 | Milk capric acid content | 22056641-22056645 | Holstein |
| 26 | Association | QTL:195232 | Milk myristic acid content | 22056641-22056645 | Holstein |
| 26 | Association | QTL:195233 | Milk C14 index | 22056641-22056645 | Holstein |
| 26 | Association | QTL:200232 | Milk myristoleic acid content | 22056641-22056645 | Holstein |
| 26 | Association | QTL:200233 | Milk C16 index | 22056641-22056645 | Holstein |
| 26 | Association | QTL:205237 | Milk palmitoleic acid content | 22056641-22056645 | Holstein |
| 26 | Association | QTL:195583 | Milk capric acid content | 22069139-22069143 | Holstein |
| 26 | Association | QTL:195584 | Milk myristic acid content | 22069139-22069143 | Holstein |
| 26 | Association | QTL:195585 | Milk C14 index | 22069139-22069143 | Holstein |
| 26 | Association | QTL:200646 | Milk myristoleic acid content | 22069139-22069143 | Holstein |
| 26 | Association | QTL:200647 | Milk C16 index | 22069139-22069143 | Holstein |
| 26 | Association | QTL:205897 | Milk palmitoleic acid content | 22069139-22069143 | Holstein |
| 26 | QTL | QTL:2573 | Milk protein yield | 14974129-27273325 |  |
| 26 | QTL | QTL:5022 | Veterinary treatments | 20730085-24646859 | Holstein |
| 26 | Association | QTL:130930 | Metabolic body weight | 22203964-22203968 | Angus |
| 26 | Association | QTL:52784 | Calving ease | 21932904-21932908 | Holstein |
| 26 | Association | QTL:52785 | Pregnancy rate | 21932904-21932908 | Holstein |
| 26 | Association | QTL:52786 | Foot angle | 21932904-21932908 | Holstein |
| 26 | Association | QTL:52787 | Feet and leg conformation | 21932904-21932908 | Holstein |
| 26 | Association | QTL:52788 | Milk fat percentage | 21932904-21932908 | Holstein |
| 26 | Association | QTL:52789 | Milk fat yield | 21932904-21932908 | Holstein |
| 26 | Association | QTL:52790 | Milk yield | 21932904-21932908 | Holstein |
| 26 | Association | QTL:52791 | Net merit | 21932904-21932908 | Holstein |
| 26 | Association | QTL:52792 | Length of productive life | 21932904-21932908 | Holstein |
| 26 | Association | QTL:52793 | Milk protein percentage | 21932904-21932908 | Holstein |
| 26 | Association | QTL:52794 | Milk protein yield | 21932904-21932908 | Holstein |
| 26 | Association | QTL:52795 | Rear leg placement - side view | 21932904-21932908 | Holstein |
| 26 | Association | QTL:52796 | Calving ease | 21932904-21932908 | Holstein |
| 26 | Association | QTL:52797 | Stillbirth | 21932904-21932908 | Holstein |
| 26 | Association | QTL:52798 | Strength | 21932904-21932908 | Holstein |
| 26 | Association | QTL:199395 | Milk capric acid content | 22086172-22086176 | Holstein |
| 26 | Association | QTL:199396 | Milk myristic acid content | 22086172-22086176 | Holstein |
| 26 | Association | QTL:199397 | Milk C14 index | 22086172-22086176 | Holstein |
| 26 | Association | QTL:204494 | Milk myristoleic acid content | 22086172-22086176 | Holstein |
| 26 | Association | QTL:204495 | Milk C16 index | 22086172-22086176 | Holstein |
| 26 | Association | QTL:210581 | Milk palmitoleic acid content | 22086172-22086176 | Holstein |
| 26 | Association | QTL:167381 | Milk C10 index | 22084475-22084479 | Holstein |
| 26 | Association | QTL:167416 | Milk C14 index | 22084475-22084479 | Holstein |
| 26 | Association | QTL:199398 | Milk capric acid content | 22084475-22084479 | Holstein |
| 26 | Association | QTL:199399 | Milk myristic acid content | 22084475-22084479 | Holstein |
| 26 | Association | QTL:199400 | Milk C14 index | 22084475-22084479 | Holstein |
| 26 | Association | QTL:204496 | Milk myristoleic acid content | 22084475-22084479 | Holstein |
| 26 | Association | QTL:204497 | Milk C16 index | 22084475-22084479 | Holstein |
| 26 | Association | QTL:210582 | Milk palmitoleic acid content | 22084475-22084479 | Holstein |
| 26 | Association | QTL:198903 | Milk capric acid content | 22095504-22095508 | Holstein |
| 26 | Association | QTL:198904 | Milk myristic acid content | 22095504-22095508 | Holstein |
| 26 | Association | QTL:198905 | Milk C14 index | 22095504-22095508 | Holstein |
| 26 | Association | QTL:203934 | Milk myristoleic acid content | 22095504-22095508 | Holstein |
| 26 | Association | QTL:203935 | Milk C16 index | 22095504-22095508 | Holstein |
| 26 | Association | QTL:210326 | Milk palmitoleic acid content | 22095504-22095508 | Holstein |
| 26 | Association | QTL:198906 | Milk capric acid content | 22096134-22096138 | Holstein |
| 26 | Association | QTL:198907 | Milk myristic acid content | 22096134-22096138 | Holstein |
| 26 | Association | QTL:198908 | Milk C14 index | 22096134-22096138 | Holstein |
| 26 | Association | QTL:203936 | Milk myristoleic acid content | 22096134-22096138 | Holstein |
| 26 | Association | QTL:203937 | Milk C16 index | 22096134-22096138 | Holstein |
| 26 | Association | QTL:210327 | Milk palmitoleic acid content | 22096134-22096138 | Holstein |
| 26 | Association | QTL:198909 | Milk capric acid content | 22096987-22096991 | Holstein |
| 26 | Association | QTL:198910 | Milk myristic acid content | 22096987-22096991 | Holstein |
| 26 | Association | QTL:198911 | Milk C14 index | 22096987-22096991 | Holstein |
| 26 | Association | QTL:203938 | Milk myristoleic acid content | 22096987-22096991 | Holstein |
| 26 | Association | QTL:203939 | Milk C16 index | 22096987-22096991 | Holstein |
| 26 | Association | QTL:210328 | Milk palmitoleic acid content | 22096987-22096991 | Holstein |
| 26 | Association | QTL:198912 | Milk capric acid content | 22098082-22098086 | Holstein |
| 26 | Association | QTL:198913 | Milk myristic acid content | 22098082-22098086 | Holstein |
| 26 | Association | QTL:198914 | Milk C14 index | 22098082-22098086 | Holstein |
| 26 | Association | QTL:203940 | Milk myristoleic acid content | 22098082-22098086 | Holstein |
| 26 | Association | QTL:203941 | Milk C16 index | 22098082-22098086 | Holstein |
| 26 | Association | QTL:210329 | Milk palmitoleic acid content | 22098082-22098086 | Holstein |
| 26 | Association | QTL:198915 | Milk capric acid content | 22099088-22099092 | Holstein |
| 26 | Association | QTL:198916 | Milk myristic acid content | 22099088-22099092 | Holstein |
| 26 | Association | QTL:198917 | Milk C14 index | 22099088-22099092 | Holstein |
| 26 | Association | QTL:203942 | Milk myristoleic acid content | 22099088-22099092 | Holstein |
| 26 | Association | QTL:203943 | Milk C16 index | 22099088-22099092 | Holstein |
| 26 | Association | QTL:210330 | Milk palmitoleic acid content | 22099088-22099092 | Holstein |
| 26 | Association | QTL:198351 | Milk capric acid content | 22108154-22108158 | Holstein |
| 26 | Association | QTL:198352 | Milk myristic acid content | 22108154-22108158 | Holstein |
| 26 | Association | QTL:198353 | Milk C14 index | 22108154-22108158 | Holstein |
| 26 | Association | QTL:203520 | Milk myristoleic acid content | 22108154-22108158 | Holstein |
| 26 | Association | QTL:203521 | Milk C16 index | 22108154-22108158 | Holstein |
| 26 | Association | QTL:209965 | Milk palmitoleic acid content | 22108154-22108158 | Holstein |
| 26 | Association | QTL:198918 | Milk capric acid content | 22111402-22111406 | Holstein |
| 26 | Association | QTL:198919 | Milk myristic acid content | 22111402-22111406 | Holstein |
| 26 | Association | QTL:198920 | Milk C14 index | 22111402-22111406 | Holstein |
| 26 | Association | QTL:203944 | Milk myristoleic acid content | 22111402-22111406 | Holstein |
| 26 | Association | QTL:203945 | Milk C16 index | 22111402-22111406 | Holstein |
| 26 | Association | QTL:210331 | Milk palmitoleic acid content | 22111402-22111406 | Holstein |
| 26 | Association | QTL:167382 | Milk C10 index | 22107109-22107113 | Holstein |
| 26 | Association | QTL:167417 | Milk C14 index | 22107109-22107113 | Holstein |
| 26 | Association | QTL:198921 | Milk capric acid content | 22107109-22107113 | Holstein |
| 26 | Association | QTL:198922 | Milk myristic acid content | 22107109-22107113 | Holstein |
| 26 | Association | QTL:198923 | Milk C14 index | 22107109-22107113 | Holstein |
| 26 | Association | QTL:203946 | Milk myristoleic acid content | 22107109-22107113 | Holstein |
| 26 | Association | QTL:203947 | Milk C16 index | 22107109-22107113 | Holstein |
| 26 | Association | QTL:210332 | Milk palmitoleic acid content | 22107109-22107113 | Holstein |
| 26 | Association | QTL:198924 | Milk capric acid content | 22105833-22105837 | Holstein |
| 26 | Association | QTL:198925 | Milk myristic acid content | 22105833-22105837 | Holstein |
| 26 | Association | QTL:198926 | Milk C14 index | 22105833-22105837 | Holstein |
| 26 | Association | QTL:203948 | Milk myristoleic acid content | 22105833-22105837 | Holstein |
| 26 | Association | QTL:203949 | Milk C16 index | 22105833-22105837 | Holstein |
| 26 | Association | QTL:210333 | Milk palmitoleic acid content | 22105833-22105837 | Holstein |
| 26 | Association | QTL:198243 | Milk capric acid content | 22118366-22118370 | Holstein |
| 26 | Association | QTL:198244 | Milk myristic acid content | 22118366-22118370 | Holstein |
| 26 | Association | QTL:198245 | Milk C14 index | 22118366-22118370 | Holstein |
| 26 | Association | QTL:203418 | Milk myristoleic acid content | 22118366-22118370 | Holstein |
| 26 | Association | QTL:203419 | Milk C16 index | 22118366-22118370 | Holstein |
| 26 | Association | QTL:209867 | Milk palmitoleic acid content | 22118366-22118370 | Holstein |
| 26 | Association | QTL:196097 | Milk capric acid content | 22125904-22125908 | Holstein |
| 26 | Association | QTL:196098 | Milk myristic acid content | 22125904-22125908 | Holstein |
| 26 | Association | QTL:196099 | Milk C14 index | 22125904-22125908 | Holstein |
| 26 | Association | QTL:201297 | Milk myristoleic acid content | 22125904-22125908 | Holstein |
| 26 | Association | QTL:201298 | Milk C16 index | 22125904-22125908 | Holstein |
| 26 | Association | QTL:206899 | Milk palmitoleic acid content | 22125904-22125908 | Holstein |
| 26 | Association | QTL:197582 | Milk capric acid content | 22130256-22130260 | Holstein |
| 26 | Association | QTL:197583 | Milk myristic acid content | 22130256-22130260 | Holstein |
| 26 | Association | QTL:197584 | Milk C14 index | 22130256-22130260 | Holstein |
| 26 | Association | QTL:202790 | Milk myristoleic acid content | 22130256-22130260 | Holstein |
| 26 | Association | QTL:202791 | Milk C16 index | 22130256-22130260 | Holstein |
| 26 | Association | QTL:209047 | Milk palmitoleic acid content | 22130256-22130260 | Holstein |
| 26 | Association | QTL:198962 | Milk capric acid content | 22132070-22132074 | Holstein |
| 26 | Association | QTL:198963 | Milk myristic acid content | 22132070-22132074 | Holstein |
| 26 | Association | QTL:198964 | Milk C14 index | 22132070-22132074 | Holstein |
| 26 | Association | QTL:203997 | Milk myristoleic acid content | 22132070-22132074 | Holstein |
| 26 | Association | QTL:203998 | Milk C16 index | 22132070-22132074 | Holstein |
| 26 | Association | QTL:210355 | Milk palmitoleic acid content | 22132070-22132074 | Holstein |
| 26 | Association | QTL:198965 | Milk capric acid content | 22129636-22129640 | Holstein |
| 26 | Association | QTL:198966 | Milk myristic acid content | 22129636-22129640 | Holstein |
| 26 | Association | QTL:198967 | Milk C14 index | 22129636-22129640 | Holstein |
| 26 | Association | QTL:203999 | Milk myristoleic acid content | 22129636-22129640 | Holstein |
| 26 | Association | QTL:204000 | Milk C16 index | 22129636-22129640 | Holstein |
| 26 | Association | QTL:210356 | Milk palmitoleic acid content | 22129636-22129640 | Holstein |
| 26 | Association | QTL:195732 | Milk capric acid content | 22134993-22134997 | Holstein |
| 26 | Association | QTL:195733 | Milk myristic acid content | 22134993-22134997 | Holstein |
| 26 | Association | QTL:195734 | Milk C14 index | 22134993-22134997 | Holstein |
| 26 | Association | QTL:200834 | Milk myristoleic acid content | 22134993-22134997 | Holstein |
| 26 | Association | QTL:200835 | Milk C16 index | 22134993-22134997 | Holstein |
| 26 | Association | QTL:206183 | Milk palmitoleic acid content | 22134993-22134997 | Holstein |
| 26 | Association | QTL:196411 | Milk capric acid content | 22133890-22133894 | Holstein |
| 26 | Association | QTL:196412 | Milk myristic acid content | 22133890-22133894 | Holstein |
| 26 | Association | QTL:196413 | Milk C14 index | 22133890-22133894 | Holstein |
| 26 | Association | QTL:201673 | Milk myristoleic acid content | 22133890-22133894 | Holstein |
| 26 | Association | QTL:201674 | Milk C16 index | 22133890-22133894 | Holstein |
| 26 | Association | QTL:207506 | Milk palmitoleic acid content | 22133890-22133894 | Holstein |
| 26 | Association | QTL:197809 | Milk capric acid content | 22138142-22138146 | Holstein |
| 26 | Association | QTL:197810 | Milk myristic acid content | 22138142-22138146 | Holstein |
| 26 | Association | QTL:197811 | Milk C14 index | 22138142-22138146 | Holstein |
| 26 | Association | QTL:203012 | Milk myristoleic acid content | 22138142-22138146 | Holstein |
| 26 | Association | QTL:203013 | Milk C16 index | 22138142-22138146 | Holstein |
| 26 | Association | QTL:209329 | Milk palmitoleic acid content | 22138142-22138146 | Holstein |
| 26 | Association | QTL:198927 | Milk capric acid content | 22137534-22137538 | Holstein |
| 26 | Association | QTL:198928 | Milk myristic acid content | 22137534-22137538 | Holstein |
| 26 | Association | QTL:198929 | Milk C14 index | 22137534-22137538 | Holstein |
| 26 | Association | QTL:203950 | Milk myristoleic acid content | 22137534-22137538 | Holstein |
| 26 | Association | QTL:203951 | Milk C16 index | 22137534-22137538 | Holstein |
| 26 | Association | QTL:210334 | Milk palmitoleic acid content | 22137534-22137538 | Holstein |
| 26 | Association | QTL:198959 | Milk capric acid content | 22133377-22133381 | Holstein |
| 26 | Association | QTL:198960 | Milk myristic acid content | 22133377-22133381 | Holstein |
| 26 | Association | QTL:198961 | Milk C14 index | 22133377-22133381 | Holstein |
| 26 | Association | QTL:203995 | Milk myristoleic acid content | 22133377-22133381 | Holstein |
| 26 | Association | QTL:203996 | Milk C16 index | 22133377-22133381 | Holstein |
| 26 | Association | QTL:210354 | Milk palmitoleic acid content | 22133377-22133381 | Holstein |
| 26 | Association | QTL:198968 | Milk capric acid content | 22141175-22141179 | Holstein |
| 26 | Association | QTL:198969 | Milk myristic acid content | 22141175-22141179 | Holstein |
| 26 | Association | QTL:198970 | Milk C14 index | 22141175-22141179 | Holstein |
| 26 | Association | QTL:204001 | Milk myristoleic acid content | 22141175-22141179 | Holstein |
| 26 | Association | QTL:204002 | Milk C16 index | 22141175-22141179 | Holstein |
| 26 | Association | QTL:210357 | Milk palmitoleic acid content | 22141175-22141179 | Holstein |
| 26 | Association | QTL:167383 | Milk C10 index | 22144979-22144983 | Holstein |
| 26 | Association | QTL:167418 | Milk C14 index | 22144979-22144983 | Holstein |
| 26 | Association | QTL:100440 | Milk fat yield | 22273384-22273388 | Ayrshire, Danish red, Swedish Red-and-White |
| 26 | Association | QTL:167384 | Milk C10 index | 22147549-22147553 | Holstein |
| 26 | Association | QTL:167419 | Milk C14 index | 22147549-22147553 | Holstein |
| 26 | Association | QTL:198971 | Milk capric acid content | 22147549-22147553 | Holstein |
| 26 | Association | QTL:198972 | Milk myristic acid content | 22147549-22147553 | Holstein |
| 26 | Association | QTL:198973 | Milk C14 index | 22147549-22147553 | Holstein |
| 26 | Association | QTL:204003 | Milk myristoleic acid content | 22147549-22147553 | Holstein |
| 26 | Association | QTL:204004 | Milk C16 index | 22147549-22147553 | Holstein |
| 26 | Association | QTL:210358 | Milk palmitoleic acid content | 22147549-22147553 | Holstein |
| 26 | Association | QTL:198974 | Milk capric acid content | 22150596-22150600 | Holstein |
| 26 | Association | QTL:198975 | Milk myristic acid content | 22150596-22150600 | Holstein |
| 26 | Association | QTL:198976 | Milk C14 index | 22150596-22150600 | Holstein |
| 26 | Association | QTL:204005 | Milk myristoleic acid content | 22150596-22150600 | Holstein |
| 26 | Association | QTL:204006 | Milk C16 index | 22150596-22150600 | Holstein |
| 26 | Association | QTL:210359 | Milk palmitoleic acid content | 22150596-22150600 | Holstein |
| 26 | Association | QTL:198977 | Milk capric acid content | 22151399-22151403 | Holstein |
| 26 | Association | QTL:198978 | Milk myristic acid content | 22151399-22151403 | Holstein |
| 26 | Association | QTL:198979 | Milk C14 index | 22151399-22151403 | Holstein |
| 26 | Association | QTL:204007 | Milk myristoleic acid content | 22151399-22151403 | Holstein |
| 26 | Association | QTL:204008 | Milk C16 index | 22151399-22151403 | Holstein |
| 26 | Association | QTL:210360 | Milk palmitoleic acid content | 22151399-22151403 | Holstein |
| 26 | Association | QTL:197478 | Milk capric acid content | 22159199-22159203 | Holstein |
| 26 | Association | QTL:197479 | Milk myristic acid content | 22159199-22159203 | Holstein |
| 26 | Association | QTL:197480 | Milk C14 index | 22159199-22159203 | Holstein |
| 26 | Association | QTL:202692 | Milk myristoleic acid content | 22159199-22159203 | Holstein |
| 26 | Association | QTL:202693 | Milk C16 index | 22159199-22159203 | Holstein |
| 26 | Association | QTL:208918 | Milk palmitoleic acid content | 22159199-22159203 | Holstein |
| 26 | Association | QTL:197961 | Milk capric acid content | 22154089-22154093 | Holstein |
| 26 | Association | QTL:197962 | Milk myristic acid content | 22154089-22154093 | Holstein |
| 26 | Association | QTL:197963 | Milk C14 index | 22154089-22154093 | Holstein |
| 26 | Association | QTL:203156 | Milk myristoleic acid content | 22154089-22154093 | Holstein |
| 26 | Association | QTL:203157 | Milk C16 index | 22154089-22154093 | Holstein |
| 26 | Association | QTL:209510 | Milk palmitoleic acid content | 22154089-22154093 | Holstein |
| 26 | Association | QTL:198980 | Milk capric acid content | 22160412-22160416 | Holstein |
| 26 | Association | QTL:198981 | Milk myristic acid content | 22160412-22160416 | Holstein |
| 26 | Association | QTL:198982 | Milk C14 index | 22160412-22160416 | Holstein |
| 26 | Association | QTL:204009 | Milk myristoleic acid content | 22160412-22160416 | Holstein |
| 26 | Association | QTL:204010 | Milk C16 index | 22160412-22160416 | Holstein |
| 26 | Association | QTL:210361 | Milk palmitoleic acid content | 22160412-22160416 | Holstein |
| 26 | Association | QTL:198983 | Milk capric acid content | 22160972-22160976 | Holstein |
| 26 | Association | QTL:198984 | Milk myristic acid content | 22160972-22160976 | Holstein |
| 26 | Association | QTL:198985 | Milk C14 index | 22160972-22160976 | Holstein |
| 26 | Association | QTL:204011 | Milk myristoleic acid content | 22160972-22160976 | Holstein |
| 26 | Association | QTL:204012 | Milk C16 index | 22160972-22160976 | Holstein |
| 26 | Association | QTL:210362 | Milk palmitoleic acid content | 22160972-22160976 | Holstein |
| 26 | Association | QTL:38047 | Age at first calving | 22002744-22002748 | Hanwoo |
| 26 | Association | QTL:32982 | Milk C14 index | 22010297-22010301 | Holstein |
| 26 | Association | QTL:62532 | Milk C14 index | 22010297-22010301 | Holstein |
| 26 | Association | QTL:62652 | Milk myristoleic acid content | 22010297-22010301 | Holstein |
| 26 | Association | QTL:62964 | Milk C14 index | 22010297-22010301 | Holstein |
| 26 | Association | QTL:63081 | Milk myristoleic acid content | 22010297-22010301 | Holstein |
| 26 | Association | QTL:167385 | Milk C10 index | 22165714-22165718 | Holstein |
| 26 | Association | QTL:167420 | Milk C14 index | 22165714-22165718 | Holstein |
| 26 | Association | QTL:197385 | Milk capric acid content | 22165714-22165718 | Holstein |
| 26 | Association | QTL:197386 | Milk myristic acid content | 22165714-22165718 | Holstein |
| 26 | Association | QTL:197387 | Milk C14 index | 22165714-22165718 | Holstein |
| 26 | Association | QTL:202596 | Milk myristoleic acid content | 22165714-22165718 | Holstein |
| 26 | Association | QTL:202597 | Milk C16 index | 22165714-22165718 | Holstein |
| 26 | Association | QTL:208797 | Milk palmitoleic acid content | 22165714-22165718 | Holstein |
| 26 | QTL | QTL:179041 | Bovine tuberculosis susceptibility | 22129636-22129640 | Holstein |
| 26 | Association | QTL:167386 | Milk C10 index | 22187704-22187708 | Holstein |
| 26 | Association | QTL:167421 | Milk C14 index | 22187704-22187708 | Holstein |
| 26 | Association | QTL:199056 | Milk capric acid content | 22187704-22187708 | Holstein |
| 26 | Association | QTL:199057 | Milk myristic acid content | 22187704-22187708 | Holstein |
| 26 | Association | QTL:199058 | Milk C14 index | 22187704-22187708 | Holstein |
| 26 | Association | QTL:204105 | Milk myristoleic acid content | 22187704-22187708 | Holstein |
| 26 | Association | QTL:204106 | Milk C16 index | 22187704-22187708 | Holstein |
| 26 | Association | QTL:210395 | Milk palmitoleic acid content | 22187704-22187708 | Holstein |
| 26 | Association | QTL:32864 | Milk C14 index | 22042818-22042822 | Holstein |
| 26 | Association | QTL:33494 | Milk myristoleic acid content | 22042818-22042822 | Holstein |
| 26 | Association | QTL:62487 | Milk C14 index | 22042818-22042822 | Holstein |
| 26 | Association | QTL:62610 | Milk myristoleic acid content | 22042818-22042822 | Holstein |
| 26 | Association | QTL:62895 | Milk C14 index | 22042818-22042822 | Holstein |
| 26 | Association | QTL:63021 | Milk myristoleic acid content | 22042818-22042822 | Holstein |
| 26 | Association | QTL:32865 | Milk C14 index | 22050193-22050197 | Holstein |
| 26 | Association | QTL:33495 | Milk myristoleic acid content | 22050193-22050197 | Holstein |
| 26 | Association | QTL:62488 | Milk C14 index | 22050193-22050197 | Holstein |
| 26 | Association | QTL:62611 | Milk myristoleic acid content | 22050193-22050197 | Holstein |
| 26 | Association | QTL:62896 | Milk C14 index | 22050193-22050197 | Holstein |
| 26 | Association | QTL:63022 | Milk myristoleic acid content | 22050193-22050197 | Holstein |
| 26 | Association | QTL:32866 | Milk C14 index | 22056641-22056645 | Holstein |
| 26 | Association | QTL:33496 | Milk myristoleic acid content | 22056641-22056645 | Holstein |
| 26 | Association | QTL:62489 | Milk C14 index | 22056641-22056645 | Holstein |
| 26 | Association | QTL:62612 | Milk myristoleic acid content | 22056641-22056645 | Holstein |
| 26 | Association | QTL:62897 | Milk C14 index | 22056641-22056645 | Holstein |
| 26 | Association | QTL:63023 | Milk myristoleic acid content | 22056641-22056645 | Holstein |
| 26 | Association | QTL:199059 | Milk capric acid content | 22212176-22212180 | Holstein |
| 26 | Association | QTL:199060 | Milk myristic acid content | 22212176-22212180 | Holstein |
| 26 | Association | QTL:199061 | Milk C14 index | 22212176-22212180 | Holstein |
| 26 | Association | QTL:204107 | Milk myristoleic acid content | 22212176-22212180 | Holstein |
| 26 | Association | QTL:204108 | Milk C16 index | 22212176-22212180 | Holstein |
| 26 | Association | QTL:210396 | Milk palmitoleic acid content | 22212176-22212180 | Holstein |
| 26 | Association | QTL:167387 | Milk C10 index | 22223476-22223480 | Holstein |
| 26 | Association | QTL:167422 | Milk C14 index | 22223476-22223480 | Holstein |
| 26 | Association | QTL:198284 | Milk capric acid content | 22223476-22223480 | Holstein |
| 26 | Association | QTL:198285 | Milk myristic acid content | 22223476-22223480 | Holstein |
| 26 | Association | QTL:198286 | Milk C14 index | 22223476-22223480 | Holstein |
| 26 | Association | QTL:203466 | Milk myristoleic acid content | 22223476-22223480 | Holstein |
| 26 | Association | QTL:203467 | Milk C16 index | 22223476-22223480 | Holstein |
| 26 | Association | QTL:209910 | Milk palmitoleic acid content | 22223476-22223480 | Holstein |
| 26 | Association | QTL:33034 | Milk C14 index | 22069139-22069143 | Holstein |
| 26 | Association | QTL:62479 | Milk C14 index | 22069139-22069143 | Holstein |
| 26 | Association | QTL:62634 | Milk myristoleic acid content | 22069139-22069143 | Holstein |
| 26 | Association | QTL:62945 | Milk C14 index | 22069139-22069143 | Holstein |
| 26 | Association | QTL:63112 | Milk myristoleic acid content | 22069139-22069143 | Holstein |
| 26 | Association | QTL:198031 | Milk capric acid content | 22227911-22227915 | Holstein |
| 26 | Association | QTL:198032 | Milk myristic acid content | 22227911-22227915 | Holstein |
| 26 | Association | QTL:198033 | Milk C14 index | 22227911-22227915 | Holstein |
| 26 | Association | QTL:203223 | Milk myristoleic acid content | 22227911-22227915 | Holstein |
| 26 | Association | QTL:203224 | Milk C16 index | 22227911-22227915 | Holstein |
| 26 | Association | QTL:209612 | Milk palmitoleic acid content | 22227911-22227915 | Holstein |
| 26 | Association | QTL:199062 | Milk capric acid content | 22238799-22238803 | Holstein |
| 26 | Association | QTL:199063 | Milk myristic acid content | 22238799-22238803 | Holstein |
| 26 | Association | QTL:199064 | Milk C14 index | 22238799-22238803 | Holstein |
| 26 | Association | QTL:204109 | Milk myristoleic acid content | 22238799-22238803 | Holstein |
| 26 | Association | QTL:204110 | Milk C16 index | 22238799-22238803 | Holstein |
| 26 | Association | QTL:210397 | Milk palmitoleic acid content | 22238799-22238803 | Holstein |
| 26 | Association | QTL:33036 | Milk C14 index | 22086172-22086176 | Holstein |
| 26 | Association | QTL:62467 | Milk C14 index | 22086172-22086176 | Holstein |
| 26 | Association | QTL:62641 | Milk myristoleic acid content | 22086172-22086176 | Holstein |
| 26 | Association | QTL:62934 | Milk C14 index | 22086172-22086176 | Holstein |
| 26 | Association | QTL:63117 | Milk myristoleic acid content | 22086172-22086176 | Holstein |
| 26 | Association | QTL:33035 | Milk C14 index | 22084475-22084479 | Holstein |
| 26 | Association | QTL:62480 | Milk C14 index | 22084475-22084479 | Holstein |
| 26 | Association | QTL:62635 | Milk myristoleic acid content | 22084475-22084479 | Holstein |
| 26 | Association | QTL:62946 | Milk C14 index | 22084475-22084479 | Holstein |
| 26 | Association | QTL:63113 | Milk myristoleic acid content | 22084475-22084479 | Holstein |
| 26 | Association | QTL:167389 | Milk C10 index | 22251249-22251253 | Holstein |
| 26 | Association | QTL:167424 | Milk C14 index | 22251249-22251253 | Holstein |
| 26 | Association | QTL:197591 | Milk capric acid content | 22251249-22251253 | Holstein |
| 26 | Association | QTL:197592 | Milk myristic acid content | 22251249-22251253 | Holstein |
| 26 | Association | QTL:197593 | Milk C14 index | 22251249-22251253 | Holstein |
| 26 | Association | QTL:202797 | Milk myristoleic acid content | 22251249-22251253 | Holstein |
| 26 | Association | QTL:202798 | Milk C16 index | 22251249-22251253 | Holstein |
| 26 | Association | QTL:209051 | Milk palmitoleic acid content | 22251249-22251253 | Holstein |
| 26 | Association | QTL:167361 | Milk myristoleic acid content | 22247162-22247166 | Holstein |
| 26 | Association | QTL:167388 | Milk C10 index | 22247162-22247166 | Holstein |
| 26 | Association | QTL:167423 | Milk C14 index | 22247162-22247166 | Holstein |
| 26 | Association | QTL:199065 | Milk capric acid content | 22247162-22247166 | Holstein |
| 26 | Association | QTL:199066 | Milk myristic acid content | 22247162-22247166 | Holstein |
| 26 | Association | QTL:199067 | Milk C14 index | 22247162-22247166 | Holstein |
| 26 | Association | QTL:204111 | Milk myristoleic acid content | 22247162-22247166 | Holstein |
| 26 | Association | QTL:204112 | Milk C16 index | 22247162-22247166 | Holstein |
| 26 | Association | QTL:210398 | Milk palmitoleic acid content | 22247162-22247166 | Holstein |
| 26 | Association | QTL:33037 | Milk C14 index | 22095504-22095508 | Holstein |
| 26 | Association | QTL:62468 | Milk C14 index | 22095504-22095508 | Holstein |
| 26 | Association | QTL:62642 | Milk myristoleic acid content | 22095504-22095508 | Holstein |
| 26 | Association | QTL:62935 | Milk C14 index | 22095504-22095508 | Holstein |
| 26 | Association | QTL:63118 | Milk myristoleic acid content | 22095504-22095508 | Holstein |
| 26 | Association | QTL:33038 | Milk C14 index | 22096134-22096138 | Holstein |
| 26 | Association | QTL:62469 | Milk C14 index | 22096134-22096138 | Holstein |
| 26 | Association | QTL:62643 | Milk myristoleic acid content | 22096134-22096138 | Holstein |
| 26 | Association | QTL:62936 | Milk C14 index | 22096134-22096138 | Holstein |
| 26 | Association | QTL:63119 | Milk myristoleic acid content | 22096134-22096138 | Holstein |
| 26 | Association | QTL:33039 | Milk C14 index | 22096987-22096991 | Holstein |
| 26 | Association | QTL:62470 | Milk C14 index | 22096987-22096991 | Holstein |
| 26 | Association | QTL:62644 | Milk myristoleic acid content | 22096987-22096991 | Holstein |
| 26 | Association | QTL:62937 | Milk C14 index | 22096987-22096991 | Holstein |
| 26 | Association | QTL:63120 | Milk myristoleic acid content | 22096987-22096991 | Holstein |
| 26 | Association | QTL:33040 | Milk C14 index | 22098082-22098086 | Holstein |
| 26 | Association | QTL:62471 | Milk C14 index | 22098082-22098086 | Holstein |
| 26 | Association | QTL:62645 | Milk myristoleic acid content | 22098082-22098086 | Holstein |
| 26 | Association | QTL:62938 | Milk C14 index | 22098082-22098086 | Holstein |
| 26 | Association | QTL:63121 | Milk myristoleic acid content | 22098082-22098086 | Holstein |
| 26 | Association | QTL:33041 | Milk C14 index | 22099088-22099092 | Holstein |
| 26 | Association | QTL:62472 | Milk C14 index | 22099088-22099092 | Holstein |
| 26 | Association | QTL:62646 | Milk myristoleic acid content | 22099088-22099092 | Holstein |
| 26 | Association | QTL:62939 | Milk C14 index | 22099088-22099092 | Holstein |
| 26 | Association | QTL:63122 | Milk myristoleic acid content | 22099088-22099092 | Holstein |
| 26 | Association | QTL:33043 | Milk C14 index | 22108154-22108158 | Holstein |
| 26 | Association | QTL:62474 | Milk C14 index | 22108154-22108158 | Holstein |
| 26 | Association | QTL:62648 | Milk myristoleic acid content | 22108154-22108158 | Holstein |
| 26 | Association | QTL:62941 | Milk C14 index | 22108154-22108158 | Holstein |
| 26 | Association | QTL:63124 | Milk myristoleic acid content | 22108154-22108158 | Holstein |
| 26 | Association | QTL:32521 | Milk myristoleic acid content | 22107109-22107113 |  |
| 26 | Association | QTL:32568 | Milk C14 index | 22107109-22107113 | Holstein |
| 26 | Association | QTL:33042 | Milk C14 index | 22105833-22105837 | Holstein |
| 26 | Association | QTL:62473 | Milk C14 index | 22105833-22105837 | Holstein |
| 26 | Association | QTL:62647 | Milk myristoleic acid content | 22105833-22105837 | Holstein |
| 26 | Association | QTL:62940 | Milk C14 index | 22105833-22105837 | Holstein |
| 26 | Association | QTL:63123 | Milk myristoleic acid content | 22105833-22105837 | Holstein |
| 26 | Association | QTL:33044 | Milk C14 index | 22118366-22118370 | Holstein |
| 26 | Association | QTL:62475 | Milk C14 index | 22118366-22118370 | Holstein |
| 26 | Association | QTL:62649 | Milk myristoleic acid content | 22118366-22118370 | Holstein |
| 26 | Association | QTL:62942 | Milk C14 index | 22118366-22118370 | Holstein |
| 26 | Association | QTL:63125 | Milk myristoleic acid content | 22118366-22118370 | Holstein |
| 26 | Association | QTL:33045 | Milk C14 index | 22125904-22125908 | Holstein |
| 26 | Association | QTL:62503 | Milk C14 index | 22125904-22125908 | Holstein |
| 26 | Association | QTL:62682 | Milk myristoleic acid content | 22125904-22125908 | Holstein |
| 26 | Association | QTL:62956 | Milk C14 index | 22125904-22125908 | Holstein |
| 26 | Association | QTL:63164 | Milk myristoleic acid content | 22125904-22125908 | Holstein |
| 26 | Association | QTL:33049 | Milk C14 index | 22134993-22134997 | Holstein |
| 26 | Association | QTL:62507 | Milk C14 index | 22134993-22134997 | Holstein |
| 26 | Association | QTL:62686 | Milk myristoleic acid content | 22134993-22134997 | Holstein |
| 26 | Association | QTL:62960 | Milk C14 index | 22134993-22134997 | Holstein |
| 26 | Association | QTL:63168 | Milk myristoleic acid content | 22134993-22134997 | Holstein |
| 26 | Association | QTL:33048 | Milk C14 index | 22133890-22133894 | Holstein |
| 26 | Association | QTL:62506 | Milk C14 index | 22133890-22133894 | Holstein |
| 26 | Association | QTL:62685 | Milk myristoleic acid content | 22133890-22133894 | Holstein |
| 26 | Association | QTL:62959 | Milk C14 index | 22133890-22133894 | Holstein |
| 26 | Association | QTL:63167 | Milk myristoleic acid content | 22133890-22133894 | Holstein |
| 26 | Association | QTL:33131 | Milk C14 index | 22133377-22133381 | Holstein |
| 26 | Association | QTL:62522 | Milk C14 index | 22133377-22133381 | Holstein |
| 26 | Association | QTL:62666 | Milk myristoleic acid content | 22133377-22133381 | Holstein |
| 26 | Association | QTL:62984 | Milk C14 index | 22133377-22133381 | Holstein |
| 26 | Association | QTL:63147 | Milk myristoleic acid content | 22133377-22133381 | Holstein |
| 26 | Association | QTL:33047 | Milk C14 index | 22132070-22132074 | Holstein |
| 26 | Association | QTL:62505 | Milk C14 index | 22132070-22132074 | Holstein |
| 26 | Association | QTL:62684 | Milk myristoleic acid content | 22132070-22132074 | Holstein |
| 26 | Association | QTL:62958 | Milk C14 index | 22132070-22132074 | Holstein |
| 26 | Association | QTL:63166 | Milk myristoleic acid content | 22132070-22132074 | Holstein |
| 26 | Association | QTL:33046 | Milk C14 index | 22129636-22129640 | Holstein |
| 26 | Association | QTL:62504 | Milk C14 index | 22129636-22129640 | Holstein |
| 26 | Association | QTL:62683 | Milk myristoleic acid content | 22129636-22129640 | Holstein |
| 26 | Association | QTL:62957 | Milk C14 index | 22129636-22129640 | Holstein |
| 26 | Association | QTL:63165 | Milk myristoleic acid content | 22129636-22129640 | Holstein |
| 26 | Association | QTL:33051 | Milk C14 index | 22138142-22138146 | Holstein |
| 26 | Association | QTL:62509 | Milk C14 index | 22138142-22138146 | Holstein |
| 26 | Association | QTL:62688 | Milk myristoleic acid content | 22138142-22138146 | Holstein |
| 26 | Association | QTL:62962 | Milk C14 index | 22138142-22138146 | Holstein |
| 26 | Association | QTL:63170 | Milk myristoleic acid content | 22138142-22138146 | Holstein |
| 26 | Association | QTL:33050 | Milk C14 index | 22137534-22137538 | Holstein |
| 26 | Association | QTL:62508 | Milk C14 index | 22137534-22137538 | Holstein |
| 26 | Association | QTL:62687 | Milk myristoleic acid content | 22137534-22137538 | Holstein |
| 26 | Association | QTL:62961 | Milk C14 index | 22137534-22137538 | Holstein |
| 26 | Association | QTL:63169 | Milk myristoleic acid content | 22137534-22137538 | Holstein |
| 26 | Association | QTL:32522 | Milk myristoleic acid content | 22144979-22144983 | Holstein |
| 26 | Association | QTL:32566 | Milk C14 index | 22144979-22144983 | Holstein |
| 26 | Association | QTL:33052 | Milk C14 index | 22144979-22144983 | Holstein |
| 26 | Association | QTL:33053 | Milk C14 index | 22147549-22147553 | Holstein |
| 26 | Association | QTL:62510 | Milk C14 index | 22147549-22147553 | Holstein |
| 26 | Association | QTL:62689 | Milk myristoleic acid content | 22147549-22147553 | Holstein |
| 26 | Association | QTL:62963 | Milk C14 index | 22147549-22147553 | Holstein |
| 26 | Association | QTL:63171 | Milk myristoleic acid content | 22147549-22147553 | Holstein |
| 26 | Association | QTL:33055 | Milk C14 index | 22154089-22154093 | Holstein |
| 26 | Association | QTL:62501 | Milk C14 index | 22154089-22154093 | Holstein |
| 26 | Association | QTL:62673 | Milk myristoleic acid content | 22154089-22154093 | Holstein |
| 26 | Association | QTL:62952 | Milk C14 index | 22154089-22154093 | Holstein |
| 26 | Association | QTL:63156 | Milk myristoleic acid content | 22154089-22154093 | Holstein |
| 26 | Association | QTL:33054 | Milk C14 index | 22151399-22151403 | Holstein |
| 26 | Association | QTL:62500 | Milk C14 index | 22151399-22151403 | Holstein |
| 26 | Association | QTL:62672 | Milk myristoleic acid content | 22151399-22151403 | Holstein |
| 26 | Association | QTL:62951 | Milk C14 index | 22151399-22151403 | Holstein |
| 26 | Association | QTL:63155 | Milk myristoleic acid content | 22151399-22151403 | Holstein |
| 26 | Association | QTL:33056 | Milk C14 index | 22160412-22160416 | Holstein |
| 26 | Association | QTL:62502 | Milk C14 index | 22160412-22160416 | Holstein |
| 26 | Association | QTL:62674 | Milk myristoleic acid content | 22160412-22160416 | Holstein |
| 26 | Association | QTL:62953 | Milk C14 index | 22160412-22160416 | Holstein |
| 26 | Association | QTL:63157 | Milk myristoleic acid content | 22160412-22160416 | Holstein |
| 26 | Association | QTL:33057 | Milk C14 index | 22160972-22160976 | Holstein |
| 26 | Association | QTL:62483 | Milk C14 index | 22160972-22160976 | Holstein |
| 26 | Association | QTL:62659 | Milk myristoleic acid content | 22160972-22160976 | Holstein |
| 26 | Association | QTL:62947 | Milk C14 index | 22160972-22160976 | Holstein |
| 26 | Association | QTL:63139 | Milk myristoleic acid content | 22160972-22160976 | Holstein |
| 26 | Association | QTL:33058 | Milk C14 index | 22165714-22165718 | Holstein |
| 26 | Association | QTL:62484 | Milk C14 index | 22165714-22165718 | Holstein |
| 26 | Association | QTL:62660 | Milk myristoleic acid content | 22165714-22165718 | Holstein |
| 26 | Association | QTL:62948 | Milk C14 index | 22165714-22165718 | Holstein |
| 26 | Association | QTL:63140 | Milk myristoleic acid content | 22165714-22165718 | Holstein |
| 26 | Association | QTL:105982 | Milk protein percentage | 22467949-22467953 | Holstein |
| 26 | Association | QTL:32523 | Milk myristoleic acid content | 22187704-22187708 | Holstein |
| 26 | Association | QTL:32565 | Milk C14 index | 22187704-22187708 | Holstein |
| 26 | Association | QTL:33059 | Milk C14 index | 22187704-22187708 | Holstein |
| 26 | Association | QTL:62485 | Milk C14 index | 22187704-22187708 | Holstein |
| 26 | Association | QTL:62661 | Milk myristoleic acid content | 22187704-22187708 | Holstein |
| 26 | Association | QTL:62949 | Milk C14 index | 22187704-22187708 | Holstein |
| 26 | Association | QTL:63141 | Milk myristoleic acid content | 22187704-22187708 | Holstein |
| 26 | Association | QTL:105983 | Milk protein percentage | 22486856-22486860 | Holstein |
| 26 | Association | QTL:33060 | Milk C14 index | 22212176-22212180 | Holstein |
| 26 | Association | QTL:62482 | Milk C14 index | 22212176-22212180 | Holstein |
| 26 | Association | QTL:62665 | Milk myristoleic acid content | 22212176-22212180 | Holstein |
| 26 | Association | QTL:62950 | Milk C14 index | 22212176-22212180 | Holstein |
| 26 | Association | QTL:63144 | Milk myristoleic acid content | 22212176-22212180 | Holstein |
| 26 | Association | QTL:32899 | Milk C14 index | 22223476-22223480 | Holstein |
| 26 | Association | QTL:33667 | Milk myristoleic acid content | 22223476-22223480 | Holstein |
| 26 | Association | QTL:62497 | Milk C14 index | 22223476-22223480 | Holstein |
| 26 | Association | QTL:62609 | Milk myristoleic acid content | 22223476-22223480 | Holstein |
| 26 | Association | QTL:62908 | Milk C14 index | 22223476-22223480 | Holstein |
| 26 | Association | QTL:63031 | Milk myristoleic acid content | 22223476-22223480 | Holstein |
| 26 | Association | QTL:125828 | Lean meat yield | 22512825-22512829 | Charolais, Angus |
| 26 | Association | QTL:125829 | Subcutaneous fat thickness | 22512825-22512829 | Charolais, Angus |
| 26 | Association | QTL:33061 | Milk C14 index | 22238799-22238803 | Holstein |
| 26 | Association | QTL:62477 | Milk C14 index | 22238799-22238803 | Holstein |
| 26 | Association | QTL:62656 | Milk myristoleic acid content | 22238799-22238803 | Holstein |
| 26 | Association | QTL:62943 | Milk C14 index | 22238799-22238803 | Holstein |
| 26 | Association | QTL:63134 | Milk myristoleic acid content | 22238799-22238803 | Holstein |
| 26 | Association | QTL:33062 | Milk C14 index | 22251249-22251253 | Holstein |
| 26 | Association | QTL:62455 | Milk C14 index | 22251249-22251253 | Holstein |
| 26 | Association | QTL:62614 | Milk myristoleic acid content | 22251249-22251253 | Holstein |
| 26 | Association | QTL:62928 | Milk C14 index | 22251249-22251253 | Holstein |
| 26 | Association | QTL:63089 | Milk myristoleic acid content | 22251249-22251253 | Holstein |
| 26 | Association | QTL:24985 | Milk fat yield | 22247162-22247166 | Holstein, Jersey |
| 26 | Association | QTL:32519 | Milk myristoleic acid content | 22247162-22247166 |  |
| 26 | Association | QTL:32567 | Milk C14 index | 22247162-22247166 | Holstein |
| 26 | Association | QTL:32842 | Milk C14 index | 22247162-22247166 | Holstein |
| 26 | Association | QTL:33327 | Milk myristoleic acid content | 22247162-22247166 | Holstein |
| 26 | Association | QTL:62521 | Milk C14 index | 22247162-22247166 | Holstein |
| 26 | Association | QTL:62650 | Milk myristoleic acid content | 22247162-22247166 | Holstein |
| 26 | Association | QTL:62894 | Milk C14 index | 22247162-22247166 | Holstein |
| 26 | Association | QTL:63024 | Milk myristoleic acid content | 22247162-22247166 | Holstein |
| 26 | Association | QTL:135435 | Heat tolerance | 22512517-22512521 | Holstein |
| 26 | Association | QTL:215393 | Milk fat percentage | 22634656-22634660 | Braunvieh, Holstein, Jersey, Normande, Norwegian red, Fleckvieh, MontbÃ©liarde, Australian Red |
| 26 | Association | QTL:105817 | Milk protein percentage | 22679135-22679139 | Holstein |
| 26 | Association | QTL:99552 | Milk fat yield | 22672901-22672905 | Ayrshire, Danish red, Swedish Red-and-White |
| 26 | Association | QTL:105634 | Milk protein percentage | 22776364-22776368 | Holstein |
| 26 | Association | QTL:152392 | Saturated fatty acid content | 22685386-22685390 | Angus |
| 26 | Association | QTL:105997 | Milk protein percentage | 22845206-22845210 | Holstein |
| 26 | Association | QTL:105998 | Milk protein percentage | 22863424-22863428 | Holstein |
| 26 | Association | QTL:105999 | Milk protein percentage | 22872032-22872036 | Holstein |
| 26 | Association | QTL:215536 | Milk protein percentage | 22868293-22868297 | Braunvieh, Holstein, Jersey, Normande, Norwegian red, Fleckvieh, MontbÃ©liarde, Australian Red |
| 26 | Association | QTL:100146 | Milk fat yield | 22871994-22871998 | Ayrshire, Danish red, Swedish Red-and-White |
| 26 | Association | QTL:106000 | Milk protein percentage | 22879406-22879410 | Holstein |
| 26 | Association | QTL:105552 | Milk protein percentage | 22891565-22891569 | Holstein |
| 26 | Association | QTL:106104 | Milk protein percentage | 22914023-22914027 | Holstein |
| 26 | Association | QTL:105611 | Milk protein percentage | 22919309-22919313 | Holstein |
| 26 | Association | QTL:105612 | Milk protein percentage | 22932886-22932890 | Holstein |
| 26 | Association | QTL:105613 | Milk protein percentage | 22972000-22972004 | Holstein |
| 26 | Association | QTL:105694 | Milk protein percentage | 22982845-22982849 | Holstein |
| 26 | Association | QTL:105932 | Milk protein percentage | 23006483-23006487 | Holstein |
| 26 | Association | QTL:105695 | Milk protein percentage | 23039522-23039526 | Holstein |
| 26 | Association | QTL:147667 | Milk lactose yield | 23080741-23080745 | Holstein, Jersey |
| 26 | Association | QTL:147677 | Milk protein percentage | 23080741-23080745 | Holstein, Jersey |
| 26 | Association | QTL:147684 | Milk fat percentage | 23080741-23080745 | Holstein, Jersey |
| 26 | Association | QTL:147690 | Milk yield | 23080741-23080745 | Holstein, Jersey |
| 26 | Association | QTL:147640 | Milk lactose yield | 23090594-23090598 | Holstein, Jersey |
| 26 | QTL | QTL:12210 | Palmitoleic acid content | 17651684-59845431 | Jersey, Limousin |
| 26 | Association | QTL:34915 | Milk C14 index | 22812858-22812862 | Jersey |
| 26 | Association | QTL:100875 | Milk fat yield | 23127610-23127614 | Ayrshire, Danish red, Swedish Red-and-White |
| 26 | Association | QTL:167362 | Milk myristoleic acid content | 23017986-23017990 | Holstein |
| 26 | Association | QTL:167390 | Milk C10 index | 23017986-23017990 | Holstein |
| 26 | Association | QTL:167425 | Milk C14 index | 23017986-23017990 | Holstein |
| 26 | Association | QTL:195807 | Milk capric acid content | 23017986-23017990 | Holstein |
| 26 | Association | QTL:195808 | Milk myristic acid content | 23017986-23017990 | Holstein |
| 26 | Association | QTL:195809 | Milk C14 index | 23017986-23017990 | Holstein |
| 26 | Association | QTL:200939 | Milk myristoleic acid content | 23017986-23017990 | Holstein |
| 26 | Association | QTL:200940 | Milk C16 index | 23017986-23017990 | Holstein |
| 26 | Association | QTL:206361 | Milk palmitoleic acid content | 23017986-23017990 | Holstein |
| 26 | Association | QTL:199873 | Milk C14 index | 23162508-23162548 | Holstein |
| 26 | Association | QTL:199872 | Milk C14 index | 23167636-23167676 | Holstein |
| 26 | Association | QTL:196878 | Milk C14 index | 23171164-23171204 | Holstein |
| 26 | Association | QTL:32518 | Milk myristoleic acid content | 23017986-23017990 |  |
| 26 | Association | QTL:32561 | Milk C14 index | 23017986-23017990 | Holstein |
| 26 | Association | QTL:199955 | Milk C14 index | 23188767-23188771 | Holstein |
| 26 | Association | QTL:204963 | Milk myristoleic acid content | 23188767-23188771 | Holstein |
| 26 | Association | QTL:204964 | Milk C16 index | 23188767-23188771 | Holstein |
| 26 | Association | QTL:211209 | Milk palmitoleic acid content | 23188767-23188771 | Holstein |
| 26 | Association | QTL:196541 | Milk C14 index | 23210092-23210096 | Holstein |
| 26 | Association | QTL:201828 | Milk myristoleic acid content | 23210092-23210096 | Holstein |
| 26 | Association | QTL:201829 | Milk C16 index | 23210092-23210096 | Holstein |
| 26 | Association | QTL:207766 | Milk palmitoleic acid content | 23210092-23210096 | Holstein |
| 26 | Association | QTL:199954 | Milk C14 index | 23207266-23207270 | Holstein |
| 26 | Association | QTL:204961 | Milk myristoleic acid content | 23207266-23207270 | Holstein |
| 26 | Association | QTL:204962 | Milk C16 index | 23207266-23207270 | Holstein |
| 26 | Association | QTL:211208 | Milk palmitoleic acid content | 23207266-23207270 | Holstein |
| 26 | Association | QTL:196482 | Milk C14 index | 23224056-23224060 | Holstein |
| 26 | Association | QTL:201762 | Milk myristoleic acid content | 23224056-23224060 | Holstein |
| 26 | Association | QTL:201763 | Milk C16 index | 23224056-23224060 | Holstein |
| 26 | Association | QTL:207671 | Milk palmitoleic acid content | 23224056-23224060 | Holstein |
| 26 | Association | QTL:199952 | Milk C14 index | 23232003-23232007 | Holstein |
| 26 | Association | QTL:204957 | Milk myristoleic acid content | 23232003-23232007 | Holstein |
| 26 | Association | QTL:204958 | Milk C16 index | 23232003-23232007 | Holstein |
| 26 | Association | QTL:211206 | Milk palmitoleic acid content | 23232003-23232007 | Holstein |
| 26 | Association | QTL:167426 | Milk C14 index | 23227628-23227632 | Holstein |
| 26 | Association | QTL:199953 | Milk C14 index | 23227628-23227632 | Holstein |
| 26 | Association | QTL:204959 | Milk myristoleic acid content | 23227628-23227632 | Holstein |
| 26 | Association | QTL:204960 | Milk C16 index | 23227628-23227632 | Holstein |
| 26 | Association | QTL:211207 | Milk palmitoleic acid content | 23227628-23227632 | Holstein |
| 26 | Association | QTL:100813 | Milk fat yield | 23189642-23189646 | Ayrshire, Danish red, Swedish Red-and-White |
| 26 | Association | QTL:100282 | Milk fat yield | 23206860-23206864 | Ayrshire, Danish red, Swedish Red-and-White |
| 26 | Association | QTL:100545 | Milk fat yield | 23206895-23206899 | Ayrshire, Danish red, Swedish Red-and-White |
| 26 | Association | QTL:99814 | Milk fat yield | 23268176-23268180 | Ayrshire, Danish red, Swedish Red-and-White |
| 26 | Association | QTL:99678 | Milk fat yield | 23288341-23288345 | Ayrshire, Danish red, Swedish Red-and-White |
| 26 | Association | QTL:99816 | Milk fat yield | 23290454-23290458 | Ayrshire, Danish red, Swedish Red-and-White |
| 26 | Association | QTL:99775 | Milk fat yield | 23288160-23288164 | Ayrshire, Danish red, Swedish Red-and-White |
| 26 | Association | QTL:33255 | Milk C14 index | 23188767-23188771 | Holstein |
| 26 | Association | QTL:62623 | Milk C14 index | 23188767-23188771 | Holstein |
| 26 | Association | QTL:63043 | Milk C14 index | 23188767-23188771 | Holstein |
| 26 | Association | QTL:63257 | Milk myristoleic acid content | 23188767-23188771 | Holstein |
| 26 | Association | QTL:33119 | Milk C14 index | 23207266-23207270 | Holstein |
| 26 | Association | QTL:62625 | Milk C14 index | 23207266-23207270 | Holstein |
| 26 | Association | QTL:63034 | Milk C14 index | 23207266-23207270 | Holstein |
| 26 | Association | QTL:63265 | Milk myristoleic acid content | 23207266-23207270 | Holstein |
| 26 | Association | QTL:32935 | Milk C14 index | 23210092-23210096 | Holstein |
| 26 | Association | QTL:33750 | Milk myristoleic acid content | 23210092-23210096 | Holstein |
| 26 | Association | QTL:62617 | Milk C14 index | 23210092-23210096 | Holstein |
| 26 | Association | QTL:62855 | Milk myristoleic acid content | 23210092-23210096 | Holstein |
| 26 | Association | QTL:62999 | Milk C14 index | 23210092-23210096 | Holstein |
| 26 | Association | QTL:63186 | Milk myristoleic acid content | 23210092-23210096 | Holstein |
| 26 | Association | QTL:32896 | Milk C14 index | 23224056-23224060 | Holstein |
| 26 | Association | QTL:33466 | Milk myristoleic acid content | 23224056-23224060 | Holstein |
| 26 | Association | QTL:62618 | Milk C14 index | 23224056-23224060 | Holstein |
| 26 | Association | QTL:62856 | Milk myristoleic acid content | 23224056-23224060 | Holstein |
| 26 | Association | QTL:62985 | Milk C14 index | 23224056-23224060 | Holstein |
| 26 | Association | QTL:63116 | Milk myristoleic acid content | 23224056-23224060 | Holstein |
| 26 | Association | QTL:33085 | Milk C14 index | 23227628-23227632 | Holstein |
| 26 | Association | QTL:33836 | Milk myristoleic acid content | 23227628-23227632 | Holstein |
| 26 | Association | QTL:34480 | Milk C14 index | 23227628-23227632 | Jersey |
| 26 | Association | QTL:62626 | Milk C14 index | 23227628-23227632 | Holstein |
| 26 | Association | QTL:63025 | Milk C14 index | 23227628-23227632 | Holstein |
| 26 | Association | QTL:63236 | Milk myristoleic acid content | 23227628-23227632 | Holstein |
| 26 | Association | QTL:32973 | Milk C14 index | 23232003-23232007 | Holstein |
| 26 | Association | QTL:33904 | Milk myristoleic acid content | 23232003-23232007 | Holstein |
| 26 | Association | QTL:62624 | Milk C14 index | 23232003-23232007 | Holstein |
| 26 | Association | QTL:63014 | Milk C14 index | 23232003-23232007 | Holstein |
| 26 | Association | QTL:63205 | Milk myristoleic acid content | 23232003-23232007 | Holstein |
| 26 | Association | QTL:34316 | Milk C14 index | 23240244-23240248 | Jersey |
| 26 | Association | QTL:34226 | Milk C14 index | 23248505-23248509 | Jersey |
| 26 | QTL | QTL:10192 | Milk fat yield | 7008275-42377141 | Holstein |
| 26 | QTL | QTL:2574 | Milk yield | 14974129-27273325 |  |
| 26 | QTL | QTL:1716 | Calving ease | 1859485-27273325 |  |
| 26 | QTL | QTL:11234 | Body weight | 19400477-34353648 | Angus |
| 26 | QTL | QTL:11233 | Body weight | 19400477-34353648 | Angus |
| 26 | QTL | QTL:11232 | Body weight | 19400477-27823507 | Angus |
| 26 | QTL | QTL:106481 | Pelvic area | 18289903-42765070 | Blonde d'aquitaine |
| 26 | Association | QTL:30700 | Inhibin level | 21621448-21621452 | Brahman |
| 26 | QTL | QTL:4398 | Residual feed intake | 21103423-24050828 |  |
| 26 | Association | QTL:66091 | Bovine viral diarrhea virus susceptibility | 22184138-22315134 |  |
| 26 | QTL | QTL:2598 | Milk fat percentage | 14974129-34353648 |  |
| 26 | QTL | QTL:3637 | Milk protein yield | 19400477-33842764 |  |
| 26 | QTL | QTL:12165 | Internal fat weight | 33842764-13204376 | Jersey, Limousin |
| 26 | Association | QTL:155062 | Milk palmitoleic acid content | 22125904-22125908 | Holstein |
| 26 | Association | QTL:154966 | Milk eicosapentaenoic acid content | 21490413-21490417 | Holstein |
| 26 | Association | QTL:155221 | Milk caproic acid content | 23252956-23252960 | Holstein |
| 26 | Association | QTL:155477 | Milk caprylic acid content | 23252956-23252960 | Holstein |
| 26 | QTL | QTL:5127 | Abomasum displacement | 20762834-52005332 | Holstein |
